# The Lipid Kinase Phosphatidylinositol-4 Kinase III Alpha Regulates the Phosphorylation Status of Hepatitis C Virus NS5A

**DOI:** 10.1371/journal.ppat.1003359

**Published:** 2013-05-09

**Authors:** Simon Reiss, Christian Harak, Inés Romero-Brey, Danijela Radujkovic, Rahel Klein, Alessia Ruggieri, Ilka Rebhan, Ralf Bartenschlager, Volker Lohmann

**Affiliations:** Department of Infectious Diseases, Molecular Virology, University of Heidelberg, Heidelberg, Germany; University of Washington, United States of America

## Abstract

The lipid kinase phosphatidylinositol 4-kinase III alpha (PI4KIIIα) is an essential host factor of hepatitis C virus (HCV) replication. PI4KIIIα catalyzes the synthesis of phosphatidylinositol 4-phosphate (PI4P) accumulating in HCV replicating cells due to enzyme activation resulting from its interaction with nonstructural protein 5A (NS5A). This study describes the interaction between PI4KIIIα and NS5A and its mechanistic role in viral RNA replication. We mapped the NS5A sequence involved in PI4KIIIα interaction to the carboxyterminal end of domain 1 and identified a highly conserved PI4KIIIα functional interaction site (PFIS) encompassing seven amino acids, which are essential for viral RNA replication. Mutations within this region were also impaired in NS5A-PI4KIIIα binding, reduced PI4P levels and altered the morphology of viral replication sites, reminiscent to the phenotype observed by silencing of PI4KIIIα. Interestingly, abrogation of RNA replication caused by mutations in the PFIS correlated with increased levels of hyperphosphorylated NS5A (p58), indicating that PI4KIIIα affects the phosphorylation status of NS5A. RNAi-mediated knockdown of PI4KIIIα or pharmacological ablation of kinase activity led to a relative increase of p58. In contrast, overexpression of enzymatically active PI4KIIIα increased relative abundance of basally phosphorylated NS5A (p56). PI4KIIIα therefore regulates the phosphorylation status of NS5A and viral RNA replication by favoring p56 or repressing p58 synthesis. Replication deficiencies of PFIS mutants in NS5A could not be rescued by increasing PI4P levels, but by supplying functional NS5A, supporting an essential role of PI4KIIIα in HCV replication regulating NS5A phosphorylation, thereby modulating the morphology of viral replication sites. In conclusion, we demonstrate that PI4KIIIα activity affects the NS5A phosphorylation status. Our results highlight the importance of PI4KIIIα in the morphogenesis of viral replication sites and its regulation by facilitating p56 synthesis.

## Author Summary

Hepatitis C virus (HCV) infections affect about 170 million people worldwide and often result in severe chronic liver disease. HCV is a positive-strand RNA virus inducing massive rearrangements of intracellular membranes to generate the sites of genome replication, designated the membranous web. The complex biogenesis of the membranous web is still poorly understood, but requires the concerted action of several viral nonstructural proteins and cellular factors. Recently, we and others identified the lipid kinase phosphatidylinositol-4 kinase III alpha (PI4KIIIα), catalyzing the synthesis of phosphatidylinositol 4-phosphate (PI4P), as an essential host factor involved in the formation of the membranous web. In this study, we characterized the virus-host interaction in greater detail using a genetic approach. We identified a highly conserved region in the viral phosphoprotein NS5A crucial for the interaction with PI4KIIIα. Surprisingly, we found that PI4KIIIα, despite being a lipid kinase, appeared to regulate the phosphorylation status of NS5A, thus contributing to viral replication. Our results furthermore suggest that the morphology of the membranous web is regulated by NS5A phosphorylation, providing novel insights into the complex regulation of viral RNA replication.

## Introduction

Worldwide about 170 million people are chronically infected with hepatitis C virus (HCV), a positive-strand RNA virus belonging to the *Flaviviridae* family, frequently leading to severe liver disease. The viral genome encompasses 9.6 kb and encodes mainly for a polyprotein of about 3,000 amino acids in length, flanked by nontranslated regions, which is cleaved into ten mature proteins by cellular and viral proteases (reviewed in [1], [2]): core, envelope glycoprotein 1 (E1) and E2, p7 and the six nonstructural (NS) proteins NS2, NS3, NS4A, NS4B, NS5A and NS5B. The structural proteins core, E1 and E2, which are the major constituents of the viral particle, p7, a presumed viroporin, and NS2, which is part of the protease mediating NS2/NS3 cleavage, are mainly involved in the generation of infectious virions, whereas NS3 to NS5B are required for RNA replication. NS3 comprises helicase and NTPase activities in the C-terminal part and an N-terminal protease, which is constitutively bound to its cofactor NS4A. NS4B plays a major role in inducing membrane alterations that are required for viral replication (reviewed in [3]). NS5A is a phosphoprotein consisting of three subdomains with functions in viral RNA replication and virus assembly (reviewed in [4]) and NS5B is the viral RNA-dependent RNA-polymerase (RdRP).

Viral RNA replication takes place in vesicular membrane alterations designated the membranous web (MW) [5], [6]. The morphology and biogenesis of the MW are still poorly understood, but it is believed that NS4B is the most important determinant, since sole expression of NS4B induces vesicular structures [6]. Models based on biochemical evidence and related viruses furthermore suggested that RNA synthesis takes place in membrane invaginations connected to the cytoplasm [7]–[9]. However, more recent results point to a far more complex morphology by showing that the MW mainly consists of double membrane vesicles (DMVs) and multimembrane vesicles (MMVs) [10], [11], probably including autophagosomes [10], [12], but it is currently not clear how these structures are topologically linked to RNA synthesis. The complexity of these membrane alterations, which are distinct from the vesicles induced by NS4B, suggests that the MW is generated by a concerted action of the nonstructural proteins and host factors. Indeed, NS5A and the cellular lipid kinase phosphatidylinositol 4-kinase type III alpha (PI4KIIIα) have recently been identified to contribute to the morphology and complexity of the MW [11].

PI4KIIIα (PIK4CA, PI4KA) is an ER-resident enzyme of 230–240 kD in size converting phosphatidylinositol to phosphatidylinositol 4-phosphate (PI4P). In mammalian cells, the family of PI4-kinases comprises two types with two isoforms each (PI4KIIα, PI4KIIβ, PI4KIIIα and PI4KIIIβ) differing in subcellular localization and being responsible for the synthesis of distinct PI4P pools (reviewed in [13]), in case of PI4KIIIα those in the ER, the plasma membrane [14] and parts of Golgi PI4P [15]. PI4P, besides being the precursor of other important phosphatidylinositides, is believed to play a role in trafficking by serving as a membrane code for vesicles and organelles and several proteins have been identified specifically binding to this lipid (reviewed in [16]). PI4KIIIα has been identified as an essential host factor of HCV RNA replication by a number of studies [17]–[23] arguing for a pivotal role in viral RNA synthesis. An involvement of PI4KIIIβ has been discussed as well, but might be restricted to genotype 1 and more pronounced for other steps of the viral replication cycle [20], [24]–[26]. Importantly, PI4KIIIβ and PI4P are also intimately linked to replication of enteroviruses [22], suggesting that dependence on PI metabolism and particularly PI4P is a common theme for many virus groups and opening the possibility to create broadly acting antivirals (reviewed in [27]). Some inhibitors with specificity towards PI4KIIIα [15], [28] and PI4KIIIβ [20] are available, however, since mice with conditional knockout of the PI4KIIIα gene developed lethal gastrointestinal disorders, severe side effects might be expected also for chemical inhibition of these enzymes *in vivo*, thereby restricting their use in humans [28].

The mechanistic role of PI4KIIIα in the HCV replication cycle has been addressed by several studies [11], [24], [29]. Silencing of PI4KIIIα results in a “clustered” distribution of nonstructural proteins in IF, accompanied by an altered ultrastructure of the MW that contains smaller DMVs and lacks MMVs [11]. These changes in viral replication sites have been attributed to elevated PI4P levels correlating with an altered localization of PI4P observed in the presence of HCV nonstructural proteins in cell culture and *in vivo*. PI4KIIIα directly interacts with NS5A and NS5B and *in vitro* assays suggested that lipid kinase activity is stimulated by NS5A [11], [29]. Recently, it was furthermore shown that HCV not only activates the kinase in cell culture, but also causes a depletion of PI4P in the plasma membrane, arguing for a general reorganization of cellular PI4P metabolism induced by the virus [15]. However, the molecular mechanism of PI4KIIIα activation and PI4P redistribution have not been clarified yet and it is unclear how these changes impact on MW morphology and what the precise role of NS5A is in this process.

NS5A consists of three domains (D1, 2 and 3) separated by two low complexity sequences (LCS I and LCS II) [30] and an N-terminal amphipathic helix essential for its membrane association [31]. D1 is capable of binding RNA and involved in replication [32], [33], whereas D2 is to the most part dispensable for replication [34]. D3 is important for the generation of infectious virus, probably due to an interaction with core that is regulated by NS5A phosphorylation [34]–[36]. NS5A can be found in two distinct phosphorylated forms: a basal (hypo-) and hyperphosphorylated state, designated according to their apparent molecular weight p56 and p58, respectively [37]. The phosphorylation status is directly or indirectly modulated by NS3, NS4A, NS4B and NS5B [38]–[41]. However, cellular kinases and mechanistic details regulating NS5A phosphorylation are still poorly defined. Basal phosphorylation seems to depend on kinases of the CMGC family (e.g. casein kinase (CK) II [35], [42]) and involves mainly sequences in NS5A D2 and D3 (reviewed in [4]). In contrast, p58 synthesis is mediated by the CKI protein kinase family, particularly CKIα [43], [44] and recently an additional role of Polo-like kinase 1 has been identified [45]. A cluster of serine residues encompassing amino acids (aa) 222–235 at the C-terminus of D1 and in LCS I has been shown to be involved in hyperphosphorylation of NS5A and in the regulation of RNA replication by adaptive mutations [40], [46]–[48]. Mutations in this region typically decrease p58/p56 ratio and increase RNA replication of HCV genotype 1 isolates, probably by modulating the interaction with the host factor hVAP-A [46], [48], [49]. However, a complete loss of hyperphosphorylation typically abrogates RNA replication indicating that low amount of p58 is essential for RNA replication [50], [51]. The fact that mutations causing reduced p58/p56 levels enhance replication of genotype 1 isolates [46], [48], [52], but in turn repress particle morphogenesis, raised the concept that p56 is mainly involved in RNA replication. This is corroborated by the finding that kinase inhibitors reducing NS5A hyperphosphorylation stimulate HCV replication [53]. In contrast, p58 might be a negative regulator of RNA replication and/or is required for assembly [35], [36], [54], [55].

In this study, we provide a detailed characterization of the mechanism of action of PI4KIIIα in HCV RNA replication. We identified a site in NS5A D1 that is involved in functional PI4KIIIα interaction and RNA replication. Mutations in this site phenocopied PI4KIIIα silencing, including abrogation of PI4P induction and alterations in MW morphology. Importantly the same mutations resulted in increased NS5A p58 levels and we show that enzymatic activity of PI4KIIIα plays a vital role in the modulation of NS5A phosphorylation. Transcomplementation studies suggest that alterations of NS5A phosphorylation status rather than elevation of PI4P abundance are the main determinant for efficient HCV RNA replication. These results reveal a complex mechanism of action of PI4KIIIα in HCV replication and provide novel insights into the biogenesis of the viral replication sites.

## Results

### A sequence at the C-terminus of NS5A D1 is involved in PI4KIIIα binding

**Figure 1:**
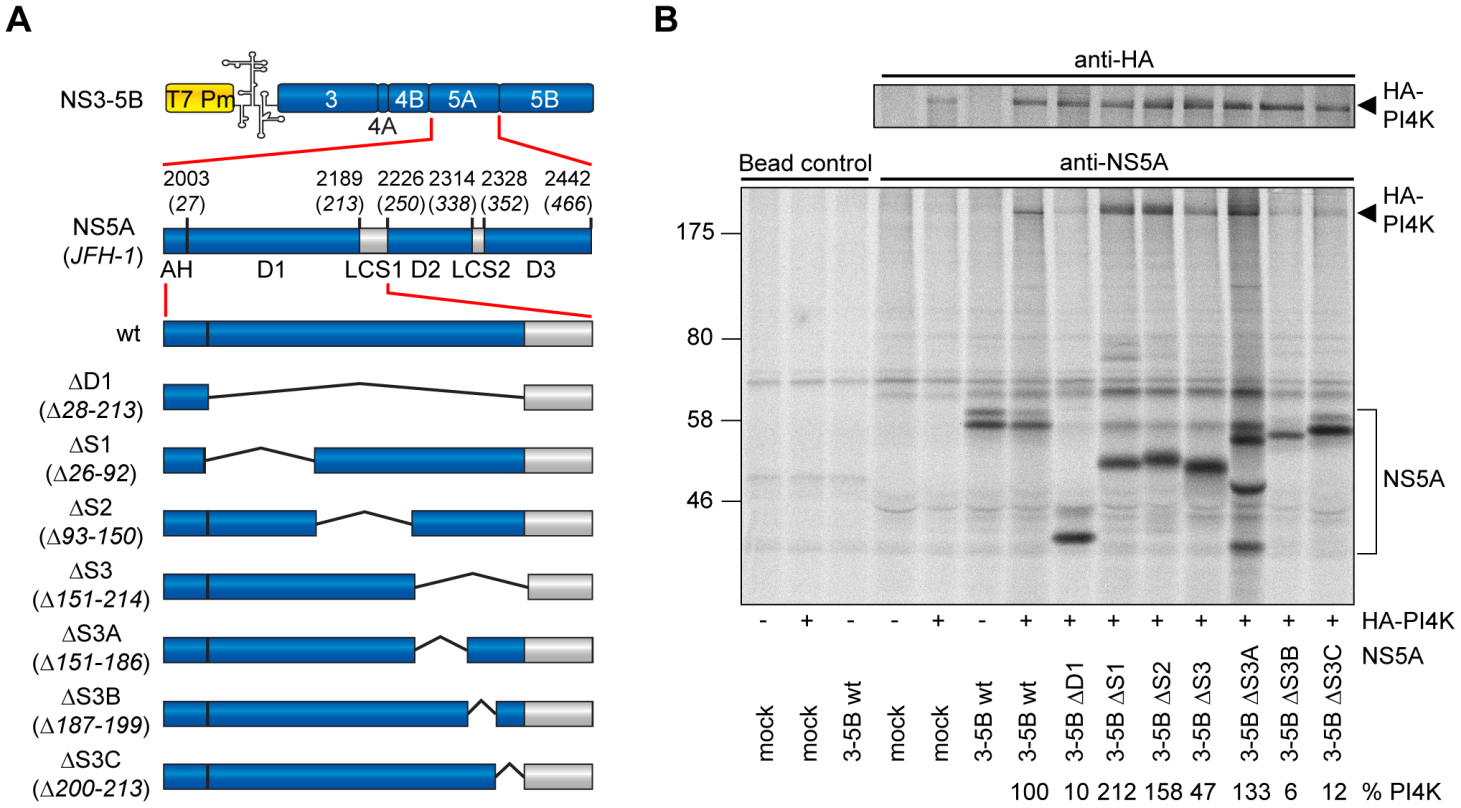
Mapping of the PI4KIIIα - interaction site within NS5A domain 1. A: Schematic representation of expression constructs with deletions in NS5A D1 used to identify the PI4KIIIα interaction site. HCV coding sequences are indicated by blue or grey boxes, deleted sequences by black lines. Numbers refer to amino acid positions within the HCV JFH-1 polyprotein, numbers in brackets to amino acid positions within NS5A. The EMCV-IRES is indicated by a schematic RNA secondary structure. T7 Pm, T7 promoter; AH, amphipathic helix; D1, 2, 3, subdomains of NS5A; LCS1, 2, low complexity sequences [30]. B: Huh7-Lunet T7 cells were transfected with plasmids encoding the NS3 to NS5B polyprotein of genotype 2a (JFH-1) with sub-deletions within NS5A domain 1 with or without HA-tagged PI4KIIIα (HA-PI4K), as indicated at the bottom. Newly synthesized proteins were radiolabeled and cell lysates subjected to immunoprecipitation using NS5A (lower panel) or HA-specific antibodies (upper panel). Samples were analyzed by SDS-PAGE and autoradiography and quantified by phosphoimaging. Numbers at the bottom indicate the coprecipitation efficiency of HA-PI4KIIIα with individual mutants compared to NS5A wt. Coprecipitation efficiency was normalized to the total amounts of HA-PI4K for each sample (upper panel). Note that data were not normalized to input NS5A levels due to a consistently high molar excess of NS5A compared to PI4KIIIα (data not shown). Mock: cells transfected with empty pTM vector.

**Figure 2:**
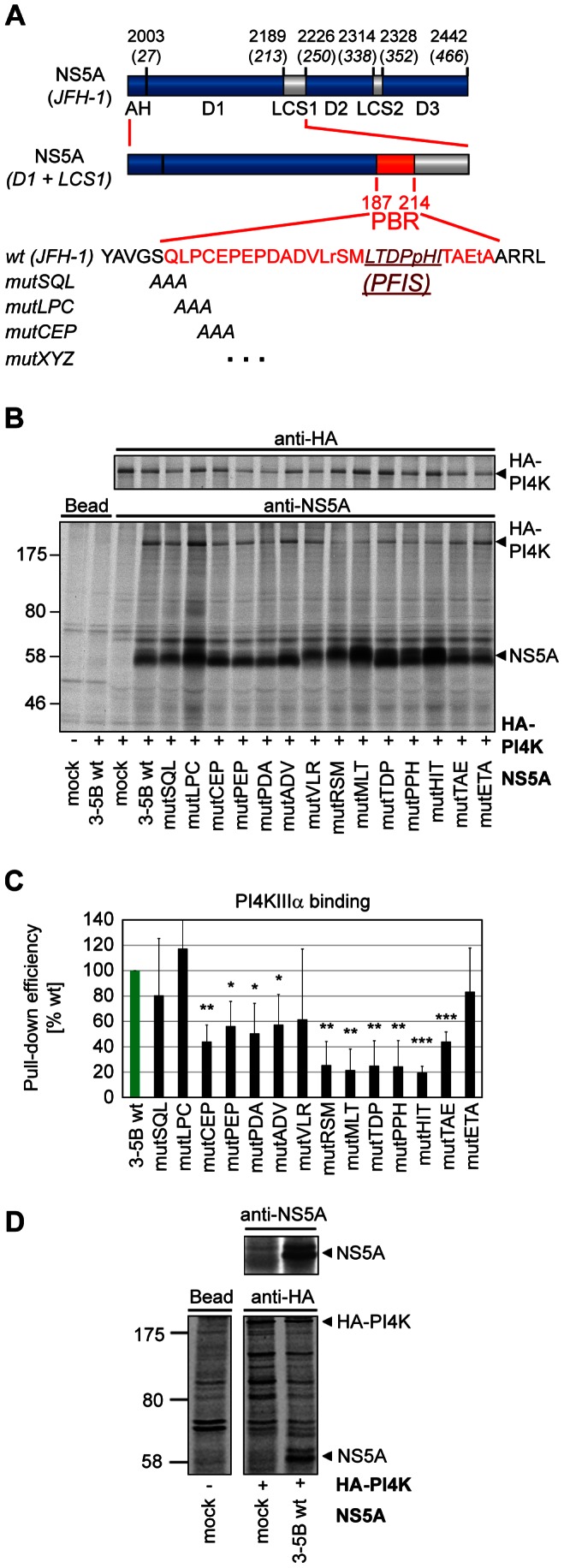
PI4KIIIα binding to NS5A triple alanine mutants of the PI4KIIIα binding region (PBR). A: Schematic representation of NS5A and NS5A D1-LCS1. The sequence encompassing deletions ΔS3B and ΔS3C (186–212) is named PI4KIIIα binding region (PBR) and is highlighted in red. PBR was subjected to closer analysis by generation of triple alanine substitutions. Mutants were designated according to the respective wt sequence as exemplified. The PI4KIIIα functional interaction site (PFIS) is indicated by a dark red line. Small letters indicate amino acids specifically found in the JFH-1 isolate. For details refer to the legend of Fig. 1A. B: Huh7-Lunet T7 cells were cotransfected with plasmids encoding the NS3 to NS5B polyprotein of genotype 2a (JFH-1) containing triple alanine mutations in NS5A domain 1 as indicated and HA-tagged PI4KIIIα (HA-PI4K). Newly synthesized proteins were radiolabeled and cell lysates subjected to immunoprecipitation using NS5A (lower panel) or HA-specific antibodies (upper panel). Samples were analyzed by SDS-PAGE and autoradiography. C: Quantitative analysis of PI4KIIIα pull-down. Experiments as shown in panel B were quantified by phosphoimaging. Coprecipitation efficiency was normalized to the total amounts of HA-PI4K and calculated relative to PI4KIIIα pull-down by NS5A wt. Note that data were not normalized to input NS5A levels due to a 20–100fold molar excess of NS5A compared to PI4KIIIα (data not shown). Error bars indicate mean values +/− SD of two independent experiments analyzed in duplicates. Significance was calculated by a paired t-test. *, p<0.05; **, p<0.01; ***, p<0.001. D: Huh7-Lunet T7 cells were transfected with plasmids encoding the NS3 to NS5B polyprotein of genotype 2a (JFH-1) and/or HA-tagged PI4KIIIα (HA-PI4K) or mock transfected as indicated. Newly synthesized proteins were radiolabeled and cell lysates subjected to immunoprecipitation using HA- (lower panel) or NS5A-specific antibodies (upper panel). Samples were analyzed by SDS-PAGE and autoradiography.

Recently, we found that PI4KIIIα directly interacts with HCV NS5A D1 but not with D2 or D3 [11]. To narrow down the NS5A binding region we designed several deletions within NS5A D1 in the context of the polyprotein NS3 to NS5B (NS3-5B) of genotype 2a (isolate JFH-1 [56]), all of them retaining the N-terminal amphipathic alpha-helix (Fig. 1A). We used a transient expression model based on plasmids encoding the HCV NS3 to NS5B polyprotein under transcriptional control of the T7-promoter, which were transfected into Huh7-Lunet cells constitutively expressing T7-RNA polymerase (Huh7-Lunet T7). This approach enabled us to examine effects of mutations on PI4KIIIα interaction independent of the requirement for functional NS5A for HCV RNA replication. Polyproteins bearing deletions within NS5A D1 were coexpressed with HA-tagged PI4KIIIα and subjected to immunoprecipitation using HA- and NS5A-specific antibodies (Fig. 1B). The amount of PI4KIIIα coprecipitating with NS5A (Fig. 1B lower panel, HA-PI4K) was quantified and normalized to PI4KIIIα input levels (Fig. 1B upper and lower panel, respectively) to determine the impact of deletions within NS5A D1 on the interaction of both proteins. None of the deletions completely abrogated NS5A- PI4KIIIα binding, indicating that determinants outside D1 might contribute to PI4KIIIα interaction. However, deletion of the entire D1 severely impaired PI4KIIIα binding, confirming our previous findings (Fig. 1B, [11]). A similar phenotype was observed upon deletion of the C-terminal part of NS5A D1 (ΔS3, Δ151–214aa), in contrast to deletions encompassing the N-terminus (ΔS1) or the center (ΔS2) of NS5A D1, which did not impair, but apparently increased PI4KIIIα binding, pointing to complex determinants contributing to the interaction of both proteins. To further narrow down the PI4KIIIα interaction site within NS5A we generated smaller deletions within aa151–214 of NS5A (ΔS3A, B and C, respectively) and found that removal of aa187–199 as well as 200–213 both reduced PI4KIIIα binding to about 10% of wt NS5A (wt). These results suggested that the very C-terminal 28aa of NS5A D1 contained sequence elements involved in the binding of PI4KIIIα, which we designated PI4KIIIα binding region (PBR, Fig. 2A).

Our next aim was to identify more subtle mutations within this region, allowing a detailed analysis of the functional role of the NS5A-PI4KIIIα interaction in HCV replication. Therefore, we generated overlapping triple alanine substitutions within the NS3-5B polyprotein (Fig. 2A) and first analyzed their impact on NS5A-PI4KIIIα binding (Fig. 2B, C, D). Interestingly, all mutations except mutSQL, LPC, VLR and ETA significantly reduced PI4KIIIα coprecipitation efficiency, supporting our assumption that this whole region was involved in PI4KIIIα binding. However, the effect of most NS5A triple alanine mutations was not very strong and reduced the efficiency of PI4KIIIα binding by about 50%. Only a set of mutations spanning a region of nine aa reduced PI4KIIIα coprecipitation to about 25% of wt NS5A (Fig. 2C, Table 1). We furthermore assessed the impact of the triple alanine mutations on NS5A coprecipitating with HA-PI4KIIIα (Fig. 2D and not shown). However, the efficiency of coprecipitation was very low and was only detectable after long overexposures of the gels (Fig. 2D and not shown), most likely due to a consistently high molar excess of NS5A to HA-PI4KIIIα in all experiments (20–100fold, data not shown). Interestingly, both phosphoisoforms were precipitated with similar efficiency in case of NS5A wt. However, the signal intensities were not high enough to allow a thorough quantitation of NS5A binding and p58/p56 ratios of the NS5A mutants to draw firm conclusions (data not shown).

**Table 1:** Summary of phenotypes observed for NS5A mutants and PI4KIIIα silencing.

	Replication^1^	PI4KIIIα binding^2^	MW cluster^3^	PI4P induction^4^	p58/p56 ratio^5^
**wt**	**+++**	**+++**	**−**	**+++**	**++**
*wt + shPI4KIIIα*	*−* §	*n.a.*	*++* *	*−*	*+++*
SQL	++	++	++	+	+
LPC	+/−	+++	++	+/−	+
CEP	+++	+	++	+	++
PEP	++	++	+++	+/−	+
PDA	+++	++	+++	+	+
ADV	+++	++	+	+	+
VLR	++	++	+/−	++	++
RSM	++	+	+/−	+	++
***MLT***	***−***	***+/−***	***++***	***−***	***+++***
***TDP***	***−***	***+/−***	***++***	***−***	***+++***
***PPH***	***−***	***+/−***	***+***	***−***	***+++***
***HIT***	***−***	***+/−***	***++***	***−***	***+++***
TAE	++	+	+/−	++	++
ETA	+++	++	−	+++	++

Silencing of shPI4KIIIα is given in italics.

PFIS mutants are given in bold italics.

**Figure 3:**
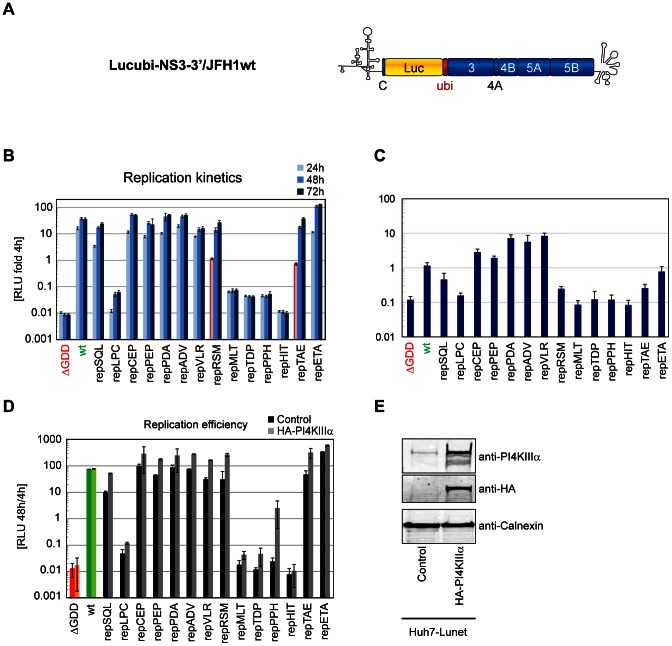
Impact of mutations within the PI4KIIIα binding region on HCV RNA replication and partial rescue by PI4KIIIα overexpression. A: Schematic representation of a monocistronic replicon used in this study. 5′ non-translated and 3′ non-translated regions are indicated by secondary structures. Luciferase (Luc) is connected by a cleavable ubiquitin (ubi) linker to the non-structural proteins NS3 to NS5B. C, core. B: Huh7-Lunet cells were transfected with luciferase reporter replicons bearing the indicated triple alanine substitutions. JFH-1 wt replicons (wt, green) and a mutant harboring a deletion within NS5B (ΔGDD, red) served as positive and negative controls, respectively. Replication efficiency is expressed as luciferase activity (RLU) at 24 h (light color), 48 h (medium color) and 72 h (dark color) relative to 4 h after transfection to normalize for transfection efficiency. 24 h values of mutants with delayed kinetics are highlighted by red lines. Error bars indicate mean +/− SD of a representative experiment (n = 3). C: Huh7-Lunet cells were transfected with luciferase reporter replicons as described in B. Total cellular RNA was extracted 4 h or 72 h after transfection. HCV RNA was quantified by quantitative RT-PCR and is shown as HCV copies at 72 h relative to 4 h after transfection. Error bars indicate mean +/− SD of a representative experiment (n = 6). D: Naïve Huh7-Lunet cells (control) or Huh7-Lunet cells stably overexpressing HA-tagged PI4KIIIα (HA-PI4KIIIα) were transfected with luciferase reporter replicons as described in B. Replication efficiency is expressed as luciferase activity (RLU) at 48 h relative to 4 h after transfection. Error bars indicate mean +/− SD of quadruplicate values of two independent experiments. E: Overexpression of HA-PI4KIIIα was confirmed by immunoblot of whole cell lysates of naïve Huh-7 Lunet cells (control) or HA-PI4KIIIα overexpressing cells (HA-PI4KIIIα) using antibodies directed against PI4KIIIα, HA-peptide or Calnexin.

1 according to data obtained from figure 3B; +++: wt type or higher; ++: 10–99% wt; +: 1–9% wt; +/−: residual low level replication; −: no replication.

2 according to data obtained from figure 2C; +++:wt or higher; ++: 50–99%wt; +: 26–49% wt; +/−: 20–25% wt; −: below 20% wt.

**Figure 4:**
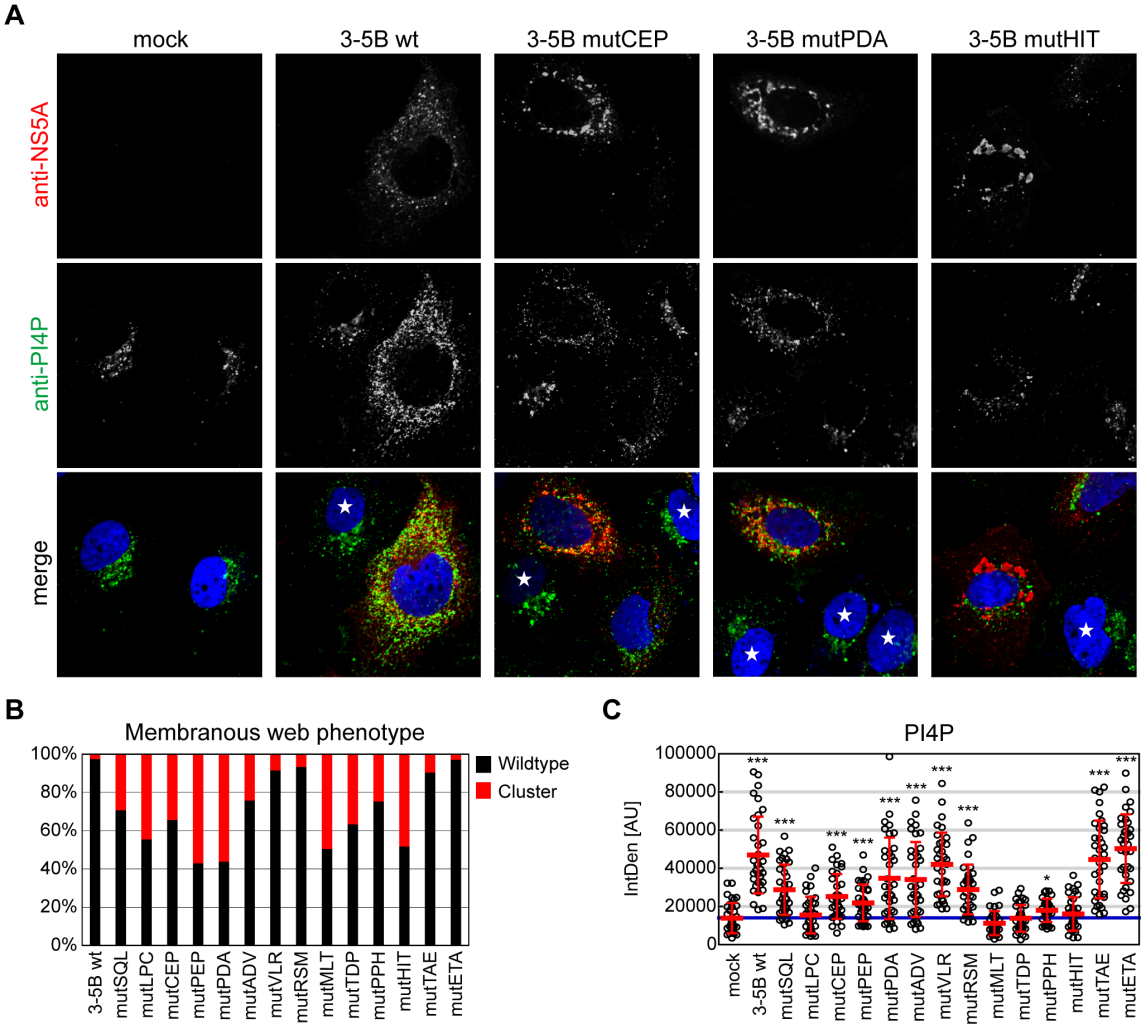
Impact of mutations within PI4KIIIα binding region on subcellular localization of NS5A and PI4P levels. A: Huh7-Lunet T7 cells were transfected with plasmids encoding the NS3 to NS5B polyprotein of genotype 2a (JFH-1) containing a wt sequence or representative triple alanine mutants as indicated or with empty plasmid (mock). 24 h post transfection NS5A (red) or PI4P (green), respectively, were detected with specific antibodies and nuclear DNA was stained with DAPI (blue). Note the punctuate NS5A staining pattern in 3-5B wt transfected cells compared to formation of NS5A “clusters” in cells expressing mutant polyproteins. Asterisks point to non-transfected cells. B: Quantitative analysis of NS5A staining patterns for wt and mutant NS3-5B polyproteins. 350 NS5A positive cells for each construct were counted and distinguished between wt (black) and clustered (red) structures by qualitative judgement, based on the representative examples for wildtype (panel A, second row) and cluster (panel A, row 3–5). C: Quantitation of intracellular PI4P levels by measuring PI4P fluorescence intensity using ImageJ analysis (IntDen read-out). Bars indicate the mean of arbitrary units (AU) +/− SD of 35 NS5A positive cells analyzed per condition. The blue line points the threshold which was set to the mean of PI4P IntDen values of untransfected cells (mock). Significance of increased PI4P levels was measured by a paired t-test and is indicated *, p<0.05; **, p<0.01; ***, p<0.001.

3 according to data obtained from figure 4B; +++:>50%; ++: 26–50%; +: 11–25%; +/−: >5–10%; −: <5% of cells with MW clusters visible in IF.

4 according to data obtained from figure 4C; +++:>4fold mock; ++: 3–4 fold mock; +: 2–3 fold mock; +/−: >1.5–2fold mock; −: 1–1.5fold mock.

5 according to data obtained from figure 6B; +++: 1 or higher; ++: 0.5–0.9; +: 0.1–0.4.

§ Data not shown.

* According to Reiss et al., CHM 2011.

In essence, none of the triple alanine mutations within the PBR of NS5A abrogated PI4KIIIα binding entirely, suggesting that the interaction between both proteins relies on multiple determinants.

### Analysis of RNA replication efficiency of triple alanine mutants in the PBR identifies a PI4KIIIα functional interaction site (PFIS)

Next, we quantified the impact of the triple alanine mutations on HCV RNA replication using monocistronic reporter replicons of the JFH-1 isolate (Fig. 3A) using luciferase reporter assays (Fig. 3B) and direct quantitation of viral genomes after transfection of replicon RNA (Fig. 3C, Fig. S1A). Both assays revealed very similar results, except that the dynamic range of the RNA detection was lower due to large amounts of input RNA still present at 72 hours after transfection (Fig. S1A). Surprisingly, only mutations showing a strong reduction in PI4KIIIα binding were entirely incapable of replication (repMLT, TDP, PPH and HIT, Fig. 3B, 3C). Based on their replication phenotype we assumed that the region covered by mutants mutMLT, TDP, PPH and HIT was most critical for functional PI4KIIIα interaction and therefore termed this sequence motif PI4KIIIα functional interaction site (PFIS, indicated in Fig. 2A). The fact that several mutant replicons were not impaired in RNA replication at all, despite significant reductions in PI4KIIIα binding efficiency (e.g. repCEP, PEP and PDA) suggested that a minimum threshold level of PI4KIIIα interaction of more than 25% of NS5A wt was necessary and sufficient for viral RNA replication (Table 1). However, the moderately delayed replication kinetics of the two mutants flanking the PFIS (RSM, TAE; Fig. 3B) compared to their strongly reduced interaction with PI4KIIIα binding (Fig. 2C, Table 1), suggested that the determinants of functional PI4KIIIα interaction were not entirely reflected by the binding efficiency measured by co-immunoprecipitation.

In addition to the PFIS mutants, repLPC also was severely impaired in replication, but still replicated at a very low level, indicated by the increase in luciferase activity at 48 h and 72 h after transfection. However, the neighbouring mutants repSQL and repCEP were not impaired in replication at all. In addition, the PI4KIIIα binding efficiencies of these three mutants with overlapping triple alanine mutations were variable with mutLPC showing no significant reduction in PI4KIIIα binding (Fig. 2C), arguing for a role of proline_188_ in the function of NS5A independent from PI4KIIIα interaction.

To support our assumption that replication defects in the PFIS region indeed were functionally linked to PI4KIIIα, we next analyzed whether overexpression of PI4KIIIα would rescue replication of the PFIS mutants. Therefore, we used Huh7-Lunet cells constitutively overexpressing HA-PI4KIIIα upon lentiviral transduction (Fig. 3E) and compared replication efficiency of the different mutants to naïve Huh7-Lunet cells (Fig. 3D, Fig. S1B, C). Interestingly, repPPH, carrying mutations in the center of the PFIS region and being replication deficient in naïve Huh7-Lunet cells, was strongly stimulated by overexpression of PI4KIIIα, suggesting that the phenotypes observed for this mutant were indeed functionally linked to PI4KIIIα (Fig. 3D, Fig. S1B). In addition, the delayed replication kinetics of mutants RSM and TAE, flanking the PFIS, were fully rescued by overexpression of PI4KIIIα and both mutants replicated to wildtype levels already at 24 hours after electroporation (Fig. S1C). In contrast, replication efficiency of mutant LPC was not significantly enhanced by PI4KIIIα overexpression, again suggesting that the replication defect of this mutant was independent of PI4KIIIα. PFIS mutants MLT, TDP and HIT were not rescued by PI4KIIIα. This might either be due to an insufficient PI4KIIIα overexpression level, a stronger impairment of functional PI4KIIIα interaction compared to mutant PPH and/or due to defects unrelated to NS5A- PI4KIIIα interactions, like NS5A RNA binding, dimerization, replicase interactions etc. However, mutants MLT, TDP and HIT exhibited almost identical phenotypes in all kinds of analyses throughout our study compared to mutant PPH (Table 1), which was partially rescued by overexpression of PI4KIIIα. Therefore, it seemed likely that all phenotypes of the PFIS mutants besides RNA replication were indeed primarily caused by defects in functional PI4KIIIα interaction.

Triple alanine mutations in a sequence motif designated PFIS, reduced PI4KIIIα binding to about 25% of wt NS5A and completely abolished RNA replication, arguing for a mechanistic role of NS5A-PI4KIIIα interaction in viral replication. A mechanistic link between replication defects caused by mutations in the PFIS and PI4KIIIα was furthermore supported by a strong enhancement of replication efficiency of one of the PFIS mutants upon PI4KIIIα overexpression.

### Impact of mutations in the PI4KIIIα interaction region on intracellular distribution of NS5A and on PI4P abundance

Previous studies identified strongly enhanced PI4P levels in HCV-positive cells due to an activation of PI4KIIIα by NS5A [11], [29]. Silencing of PI4KIIIα abolished induction of PI4P synthesis by HCV and abrogated RNA replication. These phenotypes coincided with a so called “clustered” intracellular accumulation of HCV nonstructural proteins in immunofluorescence and an altered architecture of HCV-induced membrane alterations, suggesting a fundamental role of PI4P in the formation of the HCV replication sites [11].

To test whether our triple alanine mutations would mimic some of these PI4KIIIα knockdown phenotypes, we expressed mutant NS3-5B polyproteins in Huh7-Lunet T7 cells and costained for NS5A and PI4P to judge the localization of NS5A and to quantify the levels of PI4P. We first quantified intracellular PI4P levels in cells expressing NS3-5B compared to mock transfected cells (Fig. 4A, middle panels, Fig. 4C, Table 1). PI4P primarily localized to the Golgi-apparatus in mock-transfected cells. Expression of NS3-5B wt resulted in strongly enhanced levels of PI4P in most cells, was widely spread in the cytoplasm and partially colocalized with NS5A, as expected (Fig. 4A, C; [11]). The degree of PI4P enhancement was 4.4-fold on average, but varied widely amongst individual cells in agreement with our previous analysis [11]. Most triple alanine mutants still gave rise to significantly increased PI4P levels compared to mock-transfected cells, but to lower levels than detected in wt polyprotein-expressing cells (Fig. 4A, C, Table 1). Importantly, PI4P levels in cells transfected with mutants in the PFIS (mutMLT, TDP, PPH, HIT) were not increased compared to mock-transfected cells. The same mutations completely abrogated RNA replication (Fig. 3B, C; Table 1), suggesting that increased intracellular PI4P levels might be critical for viral RNA replication, as indicated by previous studies [11]. However, the determinants for PI4KIIIα activation could not be directly correlated to the efficiency of NS5A binding, since on one hand, mutants like LPC, which were not impaired in PI4KIIIα binding but replication deficient did not induce PI4P synthesis, whereas on the other hand mutants with strongly reduced PI4KIIIα binding capability (e.g. RSM, Fig. 2C) activated PI4KIIIα. Therefore, the determinants of PI4KIIIα activation by NS5A appeared complex and required an interaction which was not directly reflected by the binding efficiency. In addition, viral RNA replication seemed to be compatible to a wide range of intracellular PI4P levels, indicated by the huge variations already observed upon expression of NS3-5B wt and by several mutants, which were not impaired in RNA replication despite a significantly reduced ability to induce PI4P (mutSQL, CEP, PEP, PDA, ADV). To confirm this result in a replication competent model, we analyzed PI4P induction by wt and two representative mutant replicons (repCEP and PDA), replicating to the same levels, despite significant differences in mean PI4P induction levels in NS3-5B expressing cells (4.4-fold vs. 2.3- and 2.7-fold, respectively). Interestingly, results obtained with the replicons were very similar to the expression of NS3-5B. Again, the level of induction was highly variable in individual cells, but the mean value remained significantly higher for wt compared to repCEP and repPDA (3.5-fold vs 2.4- and 2.8-fold, respectively, Fig. S3B, C), arguing for a wide range of intracellular PI4P levels being compatible with efficient HCV replication.

We next analyzed the impact of the triple alanine mutations on the intracellular distribution of NS5A. In cells expressing NS3-5B wt, NS5A appeared mainly in a dot-like, dispersed pattern (Fig. 4A, upper panel), reminiscent of the distribution in replicon cells (Fig. S3A, [5]). However, ca. 3% of cells overexpressing NS3-5B wt but none of the cells harboring the wt replicon gave rise to the clustered NS5A distribution. Expression of almost all mutant polyproteins, except mutETA, resulted in a higher number of cells exhibiting the clustered phenotype (Fig. 4A, upper panel, mutCEP, PDA and HIT, Fig. 4B), coinciding with reduced levels of PI4P induction (Fig. 4C, Table 1). Since the clustered phenotype was also observed upon silencing of PI4KIIIα [11], these results suggested that a clustered distribution of NS5A was indeed linked to reduced PI4P levels. The same clustered phenotype was found using antisera directed against NS3, NS4B and NS5B, and all these nonstructural proteins co-localized to NS5A, irrespective of a dot-like or clustered distribution (Fig. S2). Therefore, cluster formation seemed to rely mainly on a reorganization of membrane structures rather than on a general disturbance of nonstructural protein interactions, which might have resulted in a loss of co-localization. The proportion of cells with NS5A clusters varied from 7–57% for the mutants compared to 3% for the wt and several mutants showing predominantly the clustered phenotype replicated like wt (e.g. mutPDA, Table 1), suggesting that this phenotype was not necessarily associated with defects in RNA replication. Therefore, we analyzed the NS5A staining pattern of two of the replication competent triple alanine mutations (mutCEP, mutPDA) in replicon-containing cells and surprisingly found no NS5A clusters at all, as in case of the wt replicon cells (Fig. S3). Probably, protein expression levels in replicon cells were not high enough to induce clusters and/or clusters were not compatible with RNA replication. Hence, these data suggest that the formation of NS5A clusters is favored by high ectopic NS3-5B polyprotein expression combined with reduced levels of PI4P, thereby pointing to mutants with reduced capability to activate PI4KIIIα.

Collectively, our data demonstrate that mutations in the PBR region of NS5A resulted in a higher abundance of a clustered NS5A distribution, as observed for PI4KIIIα knockdown, but this phenotype was not correlated with RNA replication competence and was not observed in replicon cells, suggesting that protein overexpression and reduced PI4P induction both contributed to this phenotype. We furthermore found that most mutations in the PBR affected activation of PI4KIIIα, resulting in a less pronounced enhancement of intracellular PI4P pools. RNA replication was not affected over a wide range of PI4P induction levels. However, mutations of the PFIS strongly reducing PI4KIIIα binding and not giving rise to any induction of PI4P synthesis abolished RNA replication.

### Impact of triple alanine mutations in the PBR on MW morphology

**Figure 5:**
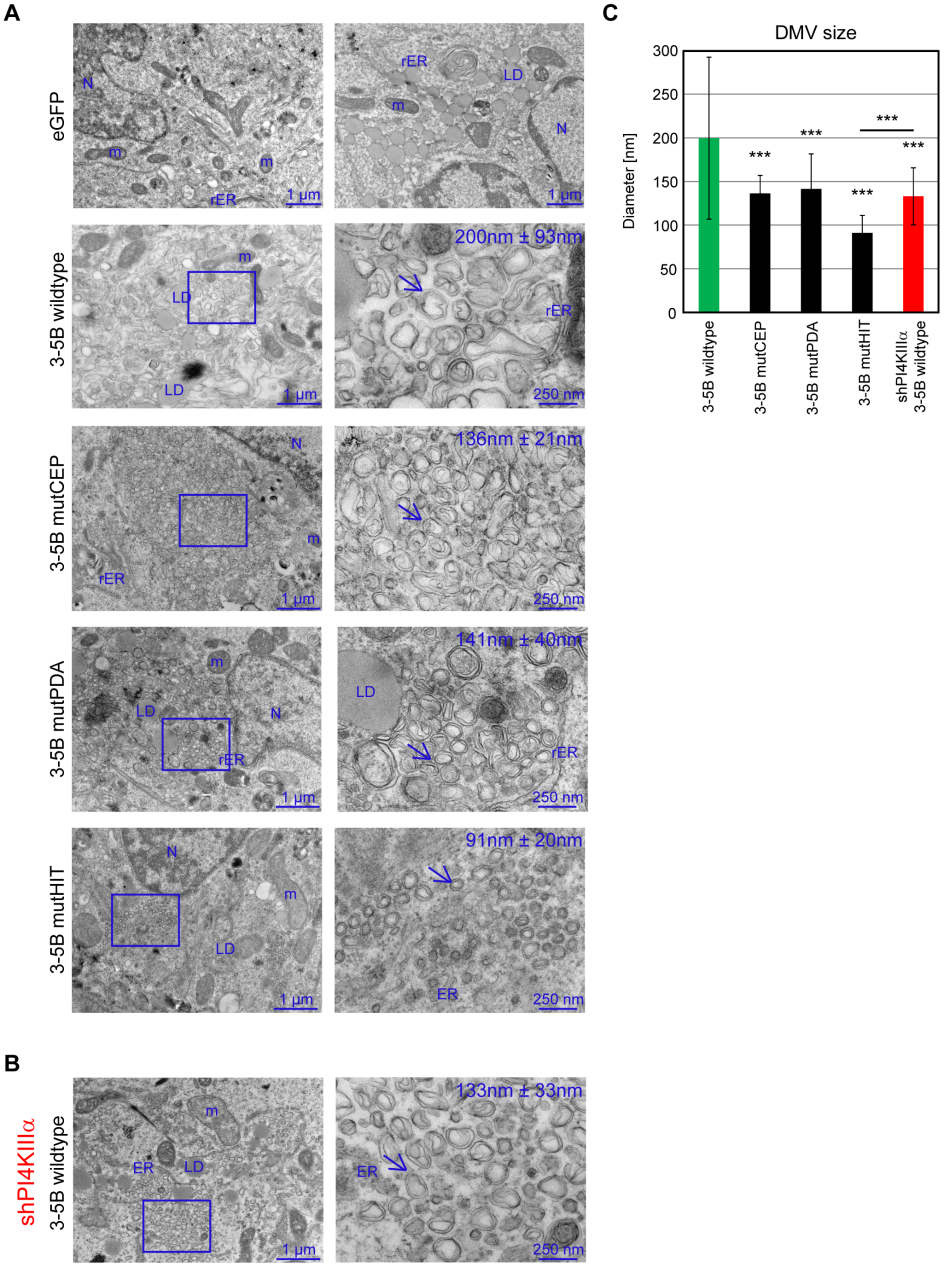
Triple alanine mutants induce ultrastructural changes similar to membranous web structures in PI4KIIIα knockdown cells. Huh7-Lunet T7 cells (A) or Huh7-Lunet T7 cells with stable PI4KIIIα knockdown (B) were transfected with pTM constructs expressing wt or mutant NS3 to NS5B polyproteins or eGFP. Cells were fixed and prepared for EM analysis 24 h post transfection. Consecutive enlargements of the boxed areas are shown from left to right. Note the heterogeneous membranous web (MW, yellow arrows) in cells expressing the wt polyprotein and the clusters of smaller double-membrane vesicles (DMVs) in shPI4KIIIα cells (B) and in cells expressing mutant polyproteins. Scale bars are given in the lower right of each panel. N, nucleus; LD, lipid droplet; rER, rough endoplasmic reticulum; m, mitochondrium. The number in the upper left of right panels shows the average diameter of 70 double-membranous vesicles (DMV) +/− SD measured for each condition. (C) Average diameter of DMVs detected in cells that had been transfected with constructs and conditions specified on the left and shown in panel A and B. Error bars indicate the mean +/− SD of seventy vesicles. Significance of differences in DMV sizes was measured by a paired t-test and is indicated ***, p<0.001.

Our previous study identified an altered morphology of MW structures upon PI4KIIIα knockdown, suggesting that PI4P was critically involved in web integrity [11]. Therefore, we next aimed to analyze MW morphology induced by triple alanine mutants of the PBR, giving rise to different levels of PI4P induction. Membrane alterations induced by expression of NS3-5B wt were heterogeneous, consisting of double membrane vesicles (DMVs) with an average diameter of 200 nm interspersed by multi-membrane vesicles (MMVs) (Fig. 5A, C [11]), Both vesicle types did not accumulate in distinct areas, but rather were dispersed throughout the cytoplasm. Very similar web structures were found in wt replicon cells, but with lower abundance, probably due to lower protein expression levels (Fig. S3D, upper panel, [11]). Silencing of PI4KIIIα resulted in more homogenous web structures, lacking MMVs, with DMVs of an average diameter of 133 nm (Fig. 5B, C), confirming our previous results [11].

For further ultrastructural analysis we chose one representative mutant in the PFIS (mutHIT) devoid of RNA replication and PI4P induction and two fully replication competent mutants with intermediate levels of PI4P induction (mutCEP and PDA). MW morphology induced by expression of NS3-5B mutHIT resembled the morphology obtained after expression of NS3-5B wt in PI4KIIIα-silenced cells: membrane alterations only contained DMVs, which were very homogeneous, but even smaller in size (91+/−20 nm, Fig. 5A, C), thus further confirming that the major phenotypes associated with this mutant indeed relied on its defects in PI4KIIIα interaction. MutCEP and mutPDA both induced web structures with an intermediate phenotype containing DMVs smaller than wt, but larger than mutHIT (136+/−21 and 142+/−40 nm, respectively) and MMVs. Expression of both mutants often resulted in vesicles accumulating in larger clusters like in shPI4KIIIα knockdown cells, in line with the high abundance of NS5A clusters detected in immunofluorescence (IF) (Fig. 4B). Membrane alterations induced by replicons harboring the CEP or PDA mutations were less abundant and more dispersed, again concordant with the IF phenotype, showing no NS5A clusters (Fig. S3A). In addition, the average diameter of the DMVs was similar to the one observed with wt replicon cells (Fig. S3D, E). This result suggests that DMVs have to adopt a minimal size to allow active RNA replication, since in case of the wt, average DMV diameter was identical for the replicon compared to the expression model.

In summary, mutations in the PFIS (e.g. mutHIT) resulted in phenotypes largely resembling and even more pronounced than knockdown of PI4KIIIα, with MW structures solely consisting of DMVs of reduced average diameter. Expression of replication-competent mutants causing a moderate reduction in PI4KIIIα activation (mutCEP and PDA) resulted in intermediate phenotypes. These results provided evidence for a critical involvement of functional NS5A-PI4KIIIα interaction in MW morphology.

### PI4KIIIα modulates NS5A phosphorylation status

**Figure 6:**
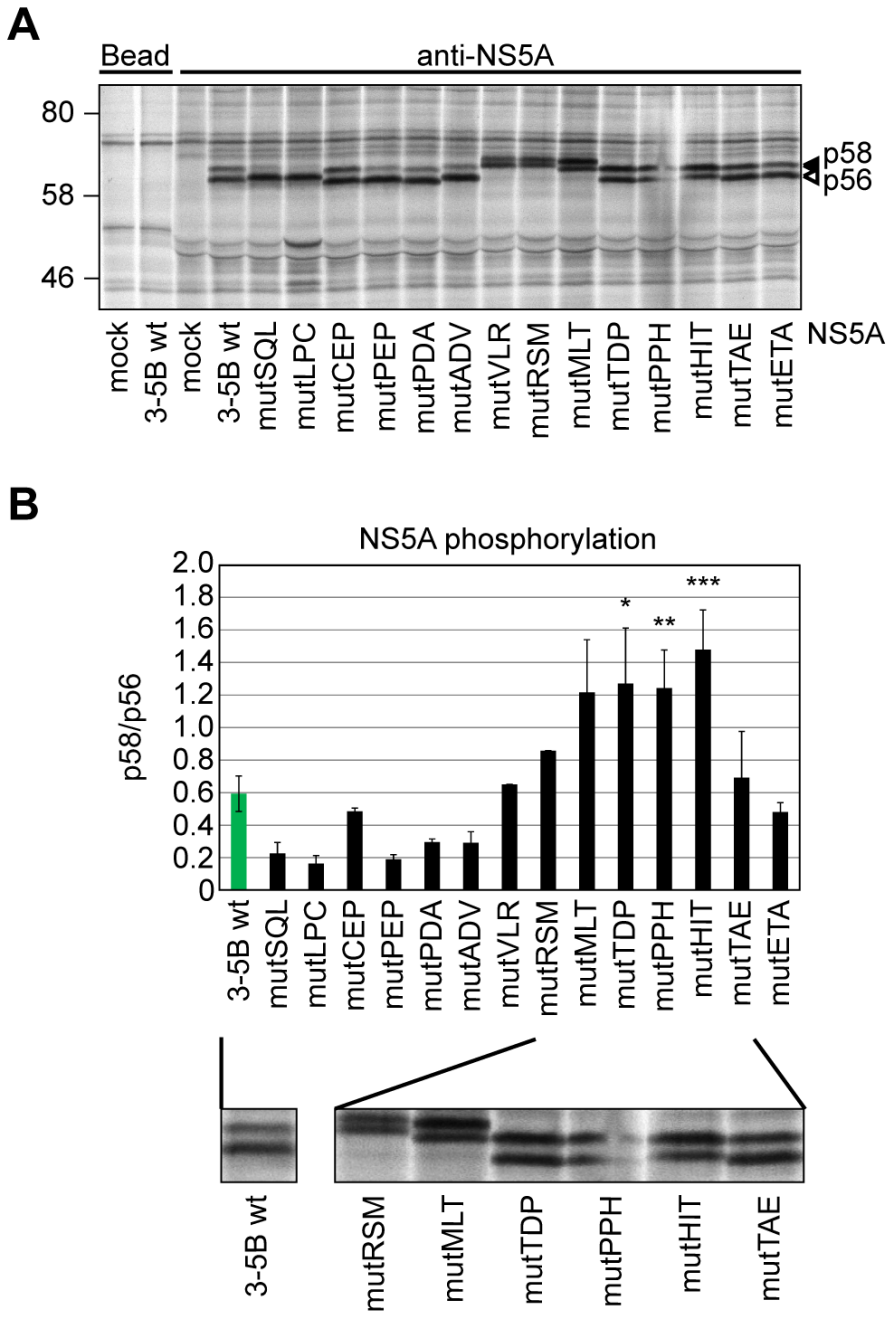
Reduced PI4KIIIα interaction and RNA replication deficiency correlate with relative increase of NS5A hyperphosphorylation. A: Huh7-Lunet T7 cells were transfected with plasmids encoding the NS3 to NS5B polyprotein of genotype 2a (JFH-1) containing a wt sequence or triple alanine mutants as indicated or with empty plasmid (mock). Newly synthesized proteins were radiolabeled and cell lysates subjected to immunoprecipitation using NS5A specific antibodies. Immunocomplexes were analyzed by SDS-PAGE and autoradiography. B: Quantitative analysis of the NS5A p58/p56 ratio. Bands corresponding to NS5A p58 and p56, respectively, as shown in panel A were individually quantified by phosphoimaging to obtain a p58/p56 ratio. Error bars indicate mean values +/− SD of two independent experiments analyzed in duplicates. Significance was compared to the wt polyprotein and calculated by a paired t-test. *, p<0.05; **, p<0.01; ***, p<0.001. A blow up of figure 6A focusing on the NS5A phosphorylation of PFIS mutants in comparison to the wildtype polyprotein is shown below.

NS5A exists in two phospho-isoforms, p56 (hypophosphorylated) and p58 (hyperphosphorylated), which can be distinguished by their electrophoretic mobility. The balance between the two different NS5A variants has been implicated in the regulation of the viral replication cycle, with p56 thought to favor RNA replication and p58 involved in assembly [46], [48], [55]. Since NS5A was interacting with PI4KIIIα and is critically involved in kinase activation, we wondered whether the phosphorylation state of NS5A might be involved in this process. Therefore, we analyzed NS5A containing triple alanine mutations in the PBR expressed in the context of the NS3-5B polyprotein for the ratio of p58/p56 (Fig. 6). Surprisingly, we found a huge variability of p58/p56 ratios among the NS5A variants, ranging from 0.2 to 1.5 (Fig. 6A, B and Table 1). The differences were indeed due to variable phosphorylation, because phosphatase treatment resulted in a reduced apparent molecular weight of all bands (Fig. S4), in contrast to a generally higher apparent molecular weight of mutants VLR, RSM and MLT, which was likely due to a change in protein conformation. NS5A wt typically gave rise to less p58 compared to p56 (roughly 40% to 60%) and had a p58/p56 ratio of 0.6, whereas most mutants with no (mutSQL, ETA, LPC) or a moderate impairment of PI4KIIIα binding (mutLPC, CEP, PEP, PDA, ADV) exhibited slightly reduced p58 levels resulting in a lower p58/p56 ratio (Fig. 6B, Fig. S3F). Importantly, all mutations in the PFIS resulting in an abrogation of RNA replication caused a strong enhancement of p58/p56 levels (Fig. 6A, B), arguing for an impact of PI4KIIIα on the phosphorylation state of NS5A. To our knowledge, these are the first reported mutations in NS5A causing an increase rather than a decrease in NS5A hyperphosphorylation. The strongly enhanced p58/p56 levels observed for the PFIS mutants furthermore suggested that impaired functional interaction of NS5A resulted in higher p58 levels, whereas a regular interaction of NS5A with PI4KIIIα might favor p56 synthesis.

**Figure 7:**
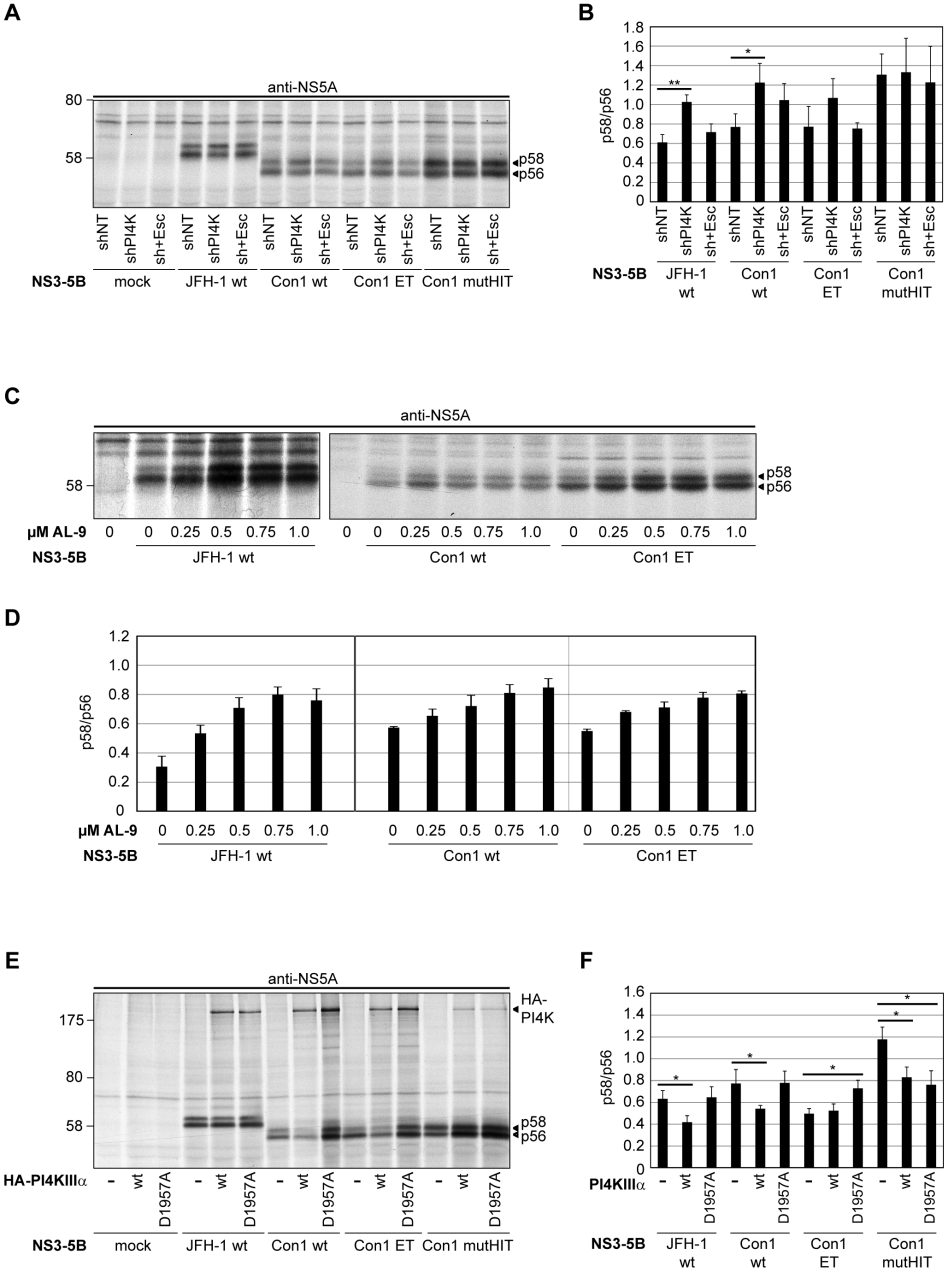
NS5A phosphorylation is influenced by PI4KIIIα enzymatic activity. A: Huh7-Lunet T7 cells expressing shRNA directed against PI4KIIIα (shPI4K) or a non-targeting control (shNT) were transfected with plasmids encoding the NS3 to NS5B polyprotein of HCV genotype 2a (JFH-1) or variants of genotype 1b (Con1 wt, ET, mutHIT). PI4KIIIα expression in shPI4K cells was reconstituted by expression of a knockdown-resistant escape variant of PI4KIIIα (sh+Esc) to exclude off-target effects. B: Quantitative analysis of the ratio of NS5A p58 and p56 obtained by phosphoimaging of experiments as shown in panel A. C: Naïve Huh7-Lunet T7 cells were transfected with plasmids encoding the NS3 to NS5B polyprotein of JFH-1 wt, Con1 wt or Con1 ET. Starting at 7 h post transfection, cells were incubated with indicated concentrations of AL-9. D: Quantitative analysis of the ratio of NS5A p58 and p56 obtained by phosphoimaging of experiments as shown in panel C. E: Huh7-Lunet T7 cells were cotransfected with plasmids encoding the NS3 to NS5B polyprotein of HCV genotype 2a (JFH-1) or variants of genotype 1b (Con1 wt, ET, mutHIT) and empty constructs (−) or plasmids encoding HA-tagged wt (wt) or inactive mutant (D1957A) PI4KIIIα. F: Quantitative analysis of the ratio of NS5A p58 and p56 obtained by phosphoimaging of experiments as shown in panel E. A, C, E: Newly synthesized proteins were radiolabeled and cell lysates subjected to immunoprecipitations using NS5A specific antibodies. Immunocomplexes were analyzed by 10% SDS-PAGE and autoradiography. B, D, F: Data represent mean values +/− SD from 2 independent experiments analyzed in duplicates. Significances were calculated by paired t-tests. *, p<0.05; **, p<0.01; ***, p<0.001.

To analyze whether the changes in NS5A phosphorylation observed for the PFIS mutants were directly associated with PI4KIIIα, we addressed the impact of PI4KIIIα knockdown and overexpression on NS5A phosphorylation (Fig. 7). We used cells with stable knockdown of PI4KIIIα by constitutive expression of sh-RNA (shPI4K); cells expressing a non-targeting control (shNT) served as negative control. For further control we restored PI4KIIIα expression in shPI4K cells by transduction with a variant of the PI4KIIIα gene resistant to knockdown due to silent mutations in the shPI4K binding site (sh+Esc). Efficient knockdown and reconstitution was confirmed by western blot for PI4KIIIα and by functional replication assays using subgenomic reporter replicons (data not shown).

Indeed, silencing of PI4KIIIα expression resulted in a significant increase in the p58/p56 ratio compared to shNT cells (Fig. 7A, B, JFH-1 wt). Importantly, the wt phenotype was restored by expression of the knockdown-resistant PI4KIIIα gene, strongly arguing for a specific impact of PI4KIIIα on the regulation of NS5A phosphorylation. The same result was obtained for two variants of the genotype 1b isolate Con1: Con1 wt, barely replicating in cell culture and Con1 ET, harboring cell culture adaptive mutations in NS3 and NS4B [52], demonstrating that modulation of the NS5A phosphorylation status by PI4KIIIα is a general feature of different HCV genotypes. We furthermore generated and tested the HIT triple alanine mutant in the PFIS of the Con1 isolate to confirm the general importance of this motif (Fig. 7A, B, Con1 mutHIT). The HIT mutation in Con1 resulted in a significantly increased p58/p56 ratio and was associated with a strongly decreased binding to PI4KIIIα. This phenotype was similar to the one found for JFH-1 (Fig. 7E, and data not shown; note the low amount of HA-PI4K coprecipitating with Con1 mutHIT), supporting the contribution of the PFIS to NS5A-PI4KIIIα binding. In contrast to JFH-1, silencing of PI4KIIIα had no impact on the p58/p56 ratio of Con1 mutHIT, which was in line with our conclusion that the increase in p58 levels of this mutant NS5A was caused by its reduced binding to PI4KIIIα.

To further confirm the role of PI4KIIIα on the regulation of NS5A phosphorylation, we tested the impact of a recently identified specific inhibitor of PI4KIIIα, AL-9 [15], on the NS5A phosphorylation status. We found very similar inhibitory concentrations of AL-9 on replication of Con1 and JFH-1 based replicons as reported [15] (IC50 ca. 0.1 and 0.5 µM, respectively). AL-9 treatment generally enhanced the abundance of NS5A, which was probably due to subtle effects of PI4KIIIα on NS5A stability, which we also observed in other experiments (e.g. Fig. 7E, compare the impact of PI4KIIIα wt overexpression on NS5A abundance to mock and inactive mutant D1957A for Con1 wt and ET). Importantly, we also found a dose-dependent increase in the p58/p56 ratios of NS5A from JFH-1, Con1 wt and Con1 ET upon AL-9 treatment (Fig. 7C, D), as expected from the knockdown experiments, thereby providing more evidence for the involvement of PI4KIIIα in regulating the phosphorylation state of NS5A. We could furthermore rule out that AL-9 had an impact on binding of NS5A to PI4KIIIα (Fig. S5), thereby supporting our assumption that indeed inhibition of the enzymatic activity of PI4KIIIα by AL-9 resulted in an increased p58/p56 ratio.

The silencing experiments and the phenotype of the PFIS mutants suggested that PI4KIIIα somehow facilitated the synthesis of p56 or suppressed the synthesis of p58. Overexpression of PI4KIIIα should therefore result in a lowering of p58/p56 ratios and we tested this hypothesis by ectopic expression of PI4KIIIα (Fig. 7E, F). In parallel to the wt gene we expressed an inactive mutant of PI4KIIIα (D1957A, [11]) to address whether kinase activity was involved in the regulation of NS5A phosphorylation. Ectopic expression of PI4KIIIα wt, but not of the D1957A mutant, indeed resulted in reduced p58/p56 ratios for JFH-1 wt and Con1 wt (Fig. 7E, F), arguing for a role of enzymatically active PI4KIIIα in NS5A phosphorylation. P58/p56 ratios were also reduced in case of Con1 mutHIT coexpressed with PI4KIIIα wt and, surprisingly, also with mutant D1957A (Fig. 7E, F). Overexpression of PI4KIIIα might therefore compensate the binding defects of the PFIS mutants to some extent, thereby reducing p58/p56 ratios, but probably also triggering changes in NS5A phosphorylation by different mechanisms as compared to NS5A wt, e.g. by preventing the access of cellular kinases involved in p58 synthesis. In case of Con1 ET, ectopic expression of PI4KIIIα wt had no impact on p58/p56 ratios, which might be due to the generally low p58/p56 ratio of NS5A Con1 ET in this experiment, consistent with a recent report [48]. Similar effects were found for some JFH-1 PBR mutants with low p58/p56 ratios (Fig. S6). In this case, p58/p56 ratios were slightly increased upon ectopic expression of PI4KIIIα for some mutants (SQL, LPC, PEP), whereas the high p58/p56 ratios of mutants in the PFIS (MLT, TDP, PPH and HIT) were reduced, as for NS5A wt (Fig. S6 compared to Fig. 6). The reduction of p58/p56 ratios of PFIS mutants by PI4KIIIα overexpression might also explain the partial rescue of RNA replication upon PI4KIIIα overexpression in case of mutant PPH (Fig. 3D).

In summary, mutants of the PFIS impaired in PI4KIIIα binding exhibited increased ratios of p58/56. The same phenotype was observed upon knockdown or pharmacological inhibition of PI4KIIIα, whereas overexpression of enzymatically active PI4KIIIα resulted in decreased p58/56 ratios. Thus, PI4KIIIα appears to affect NS5A phosphorylation either by facilitating p56 synthesis or by blocking NS5A hyperphosphorylation.

### Replication of PFIS mutants can be rescued by transcomplementation

Triple alanine mutations in the PFIS impaired NS5A-PI4KIIIα binding and completely abolished HCV RNA replication. Abrogation of RNA replication was associated with a loss of PI4P induction and increased p58/p56 ratios. The same phenotypes were observed upon PI4KIIIα knockdown or inhibition of PI4KIIIα activity by AL-9, suggesting that replication deficiency of PFIS mutants was indeed mechanistically linked to PI4KIIIα activity (Table 1). This assumption was furthermore supported by the partial rescue of RNA replication of mutant PPH upon PI4KIIIα overexpression (Fig. 3D). However, it was not clear whether induction of PI4P synthesis or regulation of NS5A phosphorylation were the key function of PI4KIIIα required for HCV replication. To get a hand on mutants with probably more distinct phenotypes we generated point mutations affecting serine and threonine residues within or surrounding the PFIS, which could be potential phosphorylation sites involved in the altered p58/p56 ratios (Fig. S7 A–D). Among those only T2185A (T210A in NS5A, affecting the threonine in mutHIT) was impaired in RNA replication, correlating with a slight reduction of PI4P induction and an increase in p58/p56 ratio. We furthermore assessed whether a phosphomimetic mutation at this site would rescue PI4KIIIα interaction and RNA replication of mutHIT, in case this threonine was phosphorylated *in vivo*. Therefore, we replaced threonine or alanine at position 210 by glutamic acid in the wildtype sequence and in mutHIT, respectively (Fig. S7E–G, mutHIE and mutAAE, respectively). However, both variants strongly interfered with PI4KIIIα binding and completely abrogated RNA replication, arguing against a phosphorylation event at this site.

**Figure 8:**
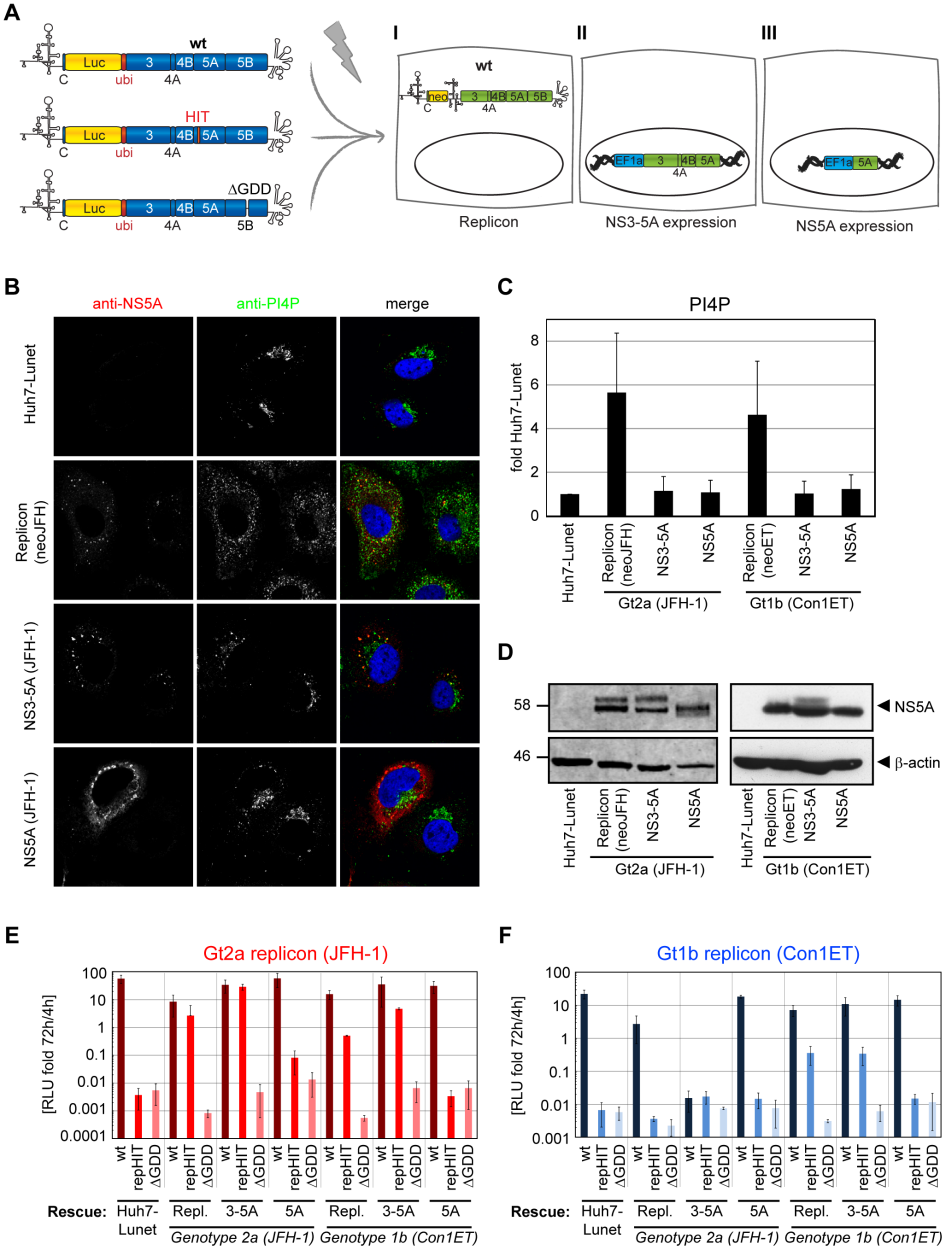
NS5A but not PI4P can transcomplement replication-deficient replicons with NS5A mutations affecting PI4KIIIα interaction. A: Experimental setup of transcomplementation experiments: Huh7-Lunet cells bearing either a persistent HCV replicon (I) or constitutively expressing HCV NS3 to NS5A (II) or NS5A (III), respectively, are transfected with luciferase reporter replicons harboring the HIT triple alanine mutation to analyze for conditions rescuing RNA replication. Wt replicons and a replication deficient NS5B mutant (ΔGDD) are used for positive and negative control, respectively. B. Huh7-Lunet cells with persistent replicons (neoJFH) or constitutive expression protein of genotype 2a NS3-5A or NS5A, respectively, were subjected to immunofluorescence analysis. NS5A (red) or PI4P (green), respectively, were detected with specific antibodies and DAPI was used to stain nuclei (blue). C. Quantitation of intracellular PI4P levels by measuring PI4P fluorescence intensity using ImageJ analysis (IntDen read-out) on cells as shown in panel B and on equivalent cell lines harboring genotype 1b (Con1) replicons and proteins. PI4P levels were normalized to the mean value of naïve Huh7-Lunet cells. Data represent mean +/− SD of 35 NS5A positive cells analyzed per condition. D. Detection of NS5A and β-actin in Huh7-Lunet cells with persistent replicons or constitutive expression of NS3-5A or NS5A of genotype (Gt) 1b and 2a, respectively, by western-blot. Equal amounts of NS5A were loaded to judge variations in NS5A phosphorylation. Differences in β-actin, therefore, reflect varying NS5A expression levels. E, F: Wt (dark color), repHIT (medium color) and ΔGDD reporter replicons (light color) of either genotype 2a (panel E, JFH-1, red) or genotype 1b (panel F, Con1ET, blue) were transfected into Huh7-Lunet cell lines harboring a persistent replicon (Repl.) or constitutively expressing NS3-5A (3-5A) or NS5A (5A) of genotype 1b or 2a, as indicated. RNA replication of replicons was determined by measuring luciferase activity in cell lysates 72 h post transfection relative to 4 h to normalize for transfection efficiency. Diagrams show mean values +/−SD of at least two experiments performed in duplicates.

Since alteration of NS5A phosphorylation and PI4P induction seemed to be intimately linked in case of the PFIS mutants, we next tried to dissect the requirements for both parameters by using transcomplementation assays. Previous studies have shown that replication-deficient mutants of NS5A, in particular those with defects in phosphorylation, can be rescued by expression of a wt protein in trans [50], [51], [57]. Minimal requirement for transcomplementation was the expression of NS5A in the context of a NS3-5A polyprotein, which was necessary and sufficient for NS5A hyperphosphorylation. In contrast, the sole expression of NS5A was not capable of rescuing deficient mutants [50], most likely due to aberrant phosphorylation, indicated by the lack of p58 [38]. Therefore, we generated six Huh7-Lunet derivatives, either containing a subgenomic replicon or constitutively expressing NS3-5A or NS5A, each based on JFH-1 or Con1 ET, respectively, to analyze which of these settings was capable of rescuing replication of a representative PFIS mutant (HIT) in the context of JFH-1 and Con1 ET reporter replicons (Fig. 8A). Wt replicons of both genotypes as well as a NS5B mutant (ΔGDD), which cannot be complemented in trans [50], were included as positive and negative controls, respectively. We first analyzed each rescue setting for induction of PI4P synthesis and NS5A phosphorylation in the absence of the transfected mutant replicons (Fig. 8B–D). Both replicon cell lines contained functional NS5A by definition, although in case of Con1 ET only p56 was clearly detectable (Fig. 8D). Both cell lines expressing NS3-5A contained two NS5A species of the expected sizes, in contrast to the cells expressing NS5A only (Fig. 8D). The replicon cell lines also contained elevated levels of PI4P (Fig. 8B, C). Surprisingly, neither expression of NS3-5A nor of NS5A increased intracellular PI4P abundance (Fig. 8B, C), despite similar NS5A levels compared to replicon cells (Fig. 8D). The lack of PI4P induction in cells expressing NS3-5A suggested that activation of PI4KIIIα requires not only NS5A, but also NS5B, which has been shown to interact with this lipid kinase as well [11].

Next, we addressed the rescue profile of the mutant replicons in the various transcomplementation settings (Fig. 8E, F). Successful rescue was judged by comparison with the ΔGDD mutant, which does not replicate and cannot be complemented in trans [50]. RepHIT JFH-1 (Fig. 8E) and repHIT Con1 ET (Fig. 8F) did not replicate in naïve Huh7-Lunet cells nor in cells expressing only NS5A, providing neither properly phosphorylated NS5A nor PI4P. Replication of repHIT JFH-1 was rescued by expression of NS3-5A and replicons of both genotypes (Fig. 8E), suggesting that the defect caused by the mutant was complemented by providing NS5A rather than by high PI4P levels, which were not provided by expression of NS3-5A (Fig. 8C). In contrast, replication of mutant repHIT Con1 ET was only rescued in cells harboring a replicon or NS3-5A of the same genotype (Fig. 8F). Expression of JFH-1 NS3-5A was even inhibitory for replication of Con1 ET wt for unknown reasons, whereas JFH-1 replicon cells supported Con1 ET wt replication, but did not rescue Con1 ET repHIT (Fig. 8F). The latter result clearly demonstrated that increased PI4P levels provided by the JFH-1 replicon cells were not sufficient to compensate for the defects caused by mutations in the PFIS and strongly argued for the necessity to provide wt NS5A, probably due to the aberrant phosphorylation induced by PFIS mutations. However, we cannot rule out the possibility that mutHIT causes additional defects in NS5A, which might require complementation by functional NS5A of the same genotype.

**Figure 9:**
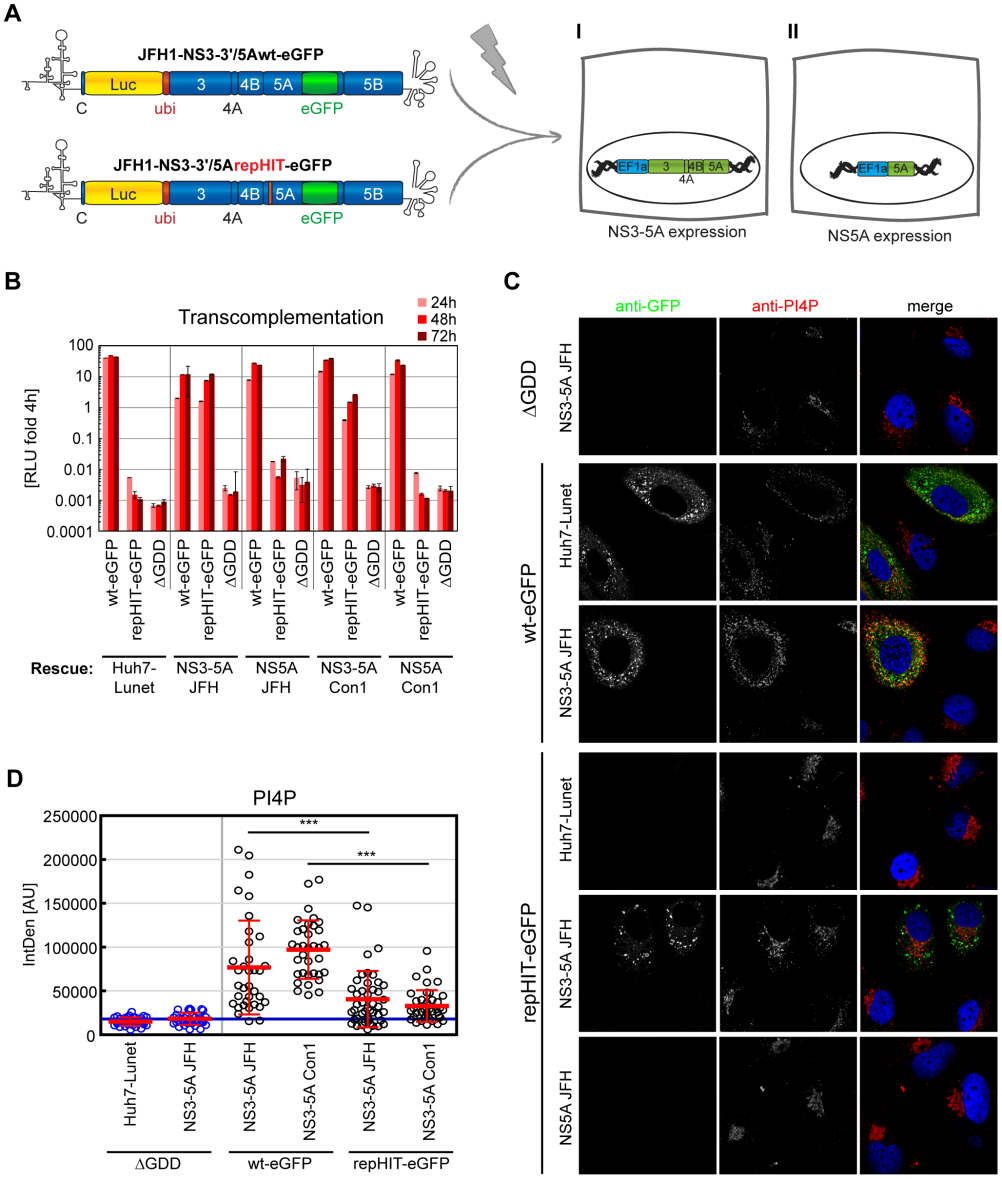
New PI4P pools emerge as a consequence of HCV RNA replication. A: Scheme of the transcomplementation experiments shown in panel B–D: Huh7-Lunet cells constitutively expressing HCV NS3 to NS5A (I) or NS5A (II), respectively, were transfected with reporter replicons containing luciferase and eGFP genes as indicated to analyze for conditions rescuing RNA replication. B: Wiltype (wt-eGFP), repHIT (repHIT-eGFP) and ΔGDD reporter replicons of genotype 2a (JFH-1) were transfected into Huh7-Lunet cell lines constitutively expressing NS3-5A or NS5A of genotype 1b or 2a, as indicated. RNA replication of replicons was determined by measuring luciferase activity in cell lysates at 24 h, 48 h and 72 h post transfection relative to 4 h to normalize for transfection efficiency. Diagrams show mean values +/−SD of a representative of two experiments performed in duplicates. C. Immunofluorescence analysis of the experiment shown in panel B at 48 h post electroporation. GFP (green) or PI4P (red), respectively, was detected with specific antibodies and DAPI was used to stain nuclei (blue). D. Quantitation of intracellular PI4P levels by measuring PI4P fluorescence intensity using ImageJ analysis (IntDen read-out) on cells as shown in panel C. Data represent mean arbitrary units (AU) +/− SD of 35 GFP positive cells analyzed per condition. In case of ΔGDD, cells were randomly chosen due to the lack of GFP signals. Significance was calculated by a paired students t-test. ***, p<0.001.

We next analyzed the impact of restored replication upon transcomplementation on intracellular PI4P levels. We used wt and HIT-mutant replicons with an eGFP inserted in NS5A [58] to unequivocally detect cells with active replication of the mutant replicon. This experiment was focused on JFH-1 replicons due to the limited replication and transcomplementation efficiency of Con1 ET and only included rescue conditions not inducing PI4P synthesis (NS5A and NS3-5A of Con1 and JFH-1, Fig. 9A). The transcomplementation pattern of JFH-1 repHIT-eGFP was identical to the non-GFP tagged variant (Fig. 9B): Replication of repHIT was restored by expression of NS3-5A, but not NS5A, of Con1 and JFH-1. Quantification of PI4P in NS5A-positive cells revealed a strong, but variable, induction of PI4P for the JFH-1 wt-eGFP replicon (Fig. 9C, D), which was about 6-fold on average (Fig. S8A) and very similar to the JFH-1 wt replicon (Fig. S3B, C) and to expression of NS3-5B wt (Fig. 4C, Table 1). Overall, the quantity of wt NS5A-eGFP correlated significantly with the amount of PI4P in individual cells (Fig. S8B, blue dots), in line with the assumption that NS5A and NS5B activate PI4KIIIα, thus giving rise to elevated levels of PI4P. In contrast, PI4P induction was much weaker upon transcomplementation of JFH-1 repHIT-eGFP (Fig. 9C, D), only 2–3 fold on average (Fig. S8A), although replication levels of the wt-eGFP replicon were identical to repHIT-eGFP in cells expressing NS3-5A JFH, as judged by luciferase counts (Fig. 9B). Many cells with bona fide HCV replication even had no detectable induction of PI4P synthesis (Fig. 9C, D, repHIT-eGFP), suggesting that strongly elevated levels of PI4P are no prerequisite of HCV replication. However, local changes in PI4P levels at the replication sites would clearly be below the detection limit of the IF based quantitation. Therefore, we cannot exclude and it even seems likely that a local elevation of PI4P levels at the replication sites is critical for viral replication, since active replication always generated conditions capable of activating PI4KIIIα. This was indicated by a clear induction of PI4P in some of the cells containing a repHIT-eGFP replicon (Fig. 9D). The amount of NS5A-eGFP and PI4P did not correlate significantly with PI4P in this case (Fig. S8B), most likely due to the fact that PI4KIIIα was activated only by wt NS5A, provided by expression of NS3-5A, but not by the mutant NS5AGFP-mutHIT, which we quantified in our analysis.

In summary, our transcomplementation analysis revealed that activation of PI4KIIIα required both NS5A and NS5B. Conditions capable of providing properly phosphorylated NS5A rescued replication of a PFIS mutant. However, elevated PI4P levels were not sufficient for successful transcomplementation and no prerequisite for rescue of a replicon with mutations in the PFIS. Still, our results indicated that active HCV replication generated conditions capable of activating PI4KIIIα. Therefore, modulation of NS5A phosphorylation as well as of PI4P metabolism by PI4KIIIα seem to be intimately linked processes and might both be required for HCV replication.

## Discussion

PI4KIIIα has been identified by multiple studies as a key host factor of HCV RNA replication [17]–[23]. Previous results revealed first insights into the potential mechanism of action by showing that PI4KIIIα interacted with NS5A [11], [29], [59], activating lipid kinase activity and resulting in increased PI4P levels with altered distribution [11], [15], [29], [60]. In this study, we identified a region at the very C-terminus of NS5A D1 involved in PI4KIIIα binding (designated PBR). Within this region, we found a site encompassing 7–9 aa (PFIS) important for functional PI4KIIIα interaction. Importantly, mutations within the PFIS caused the same phenotypes observed upon silencing or chemical inhibition of PI4KIIIα: loss of RNA replication, changes in the morphology of the replication sites [11], [15], [18] and, surprisingly, increased NS5A p58/p56 ratios, suggesting that PI4KIIIα is involved in the regulation of NS5A phosphorylation.

### The PFIS is a highly conserved sequence at the C-terminus of NS5A D1

**Figure 10:**
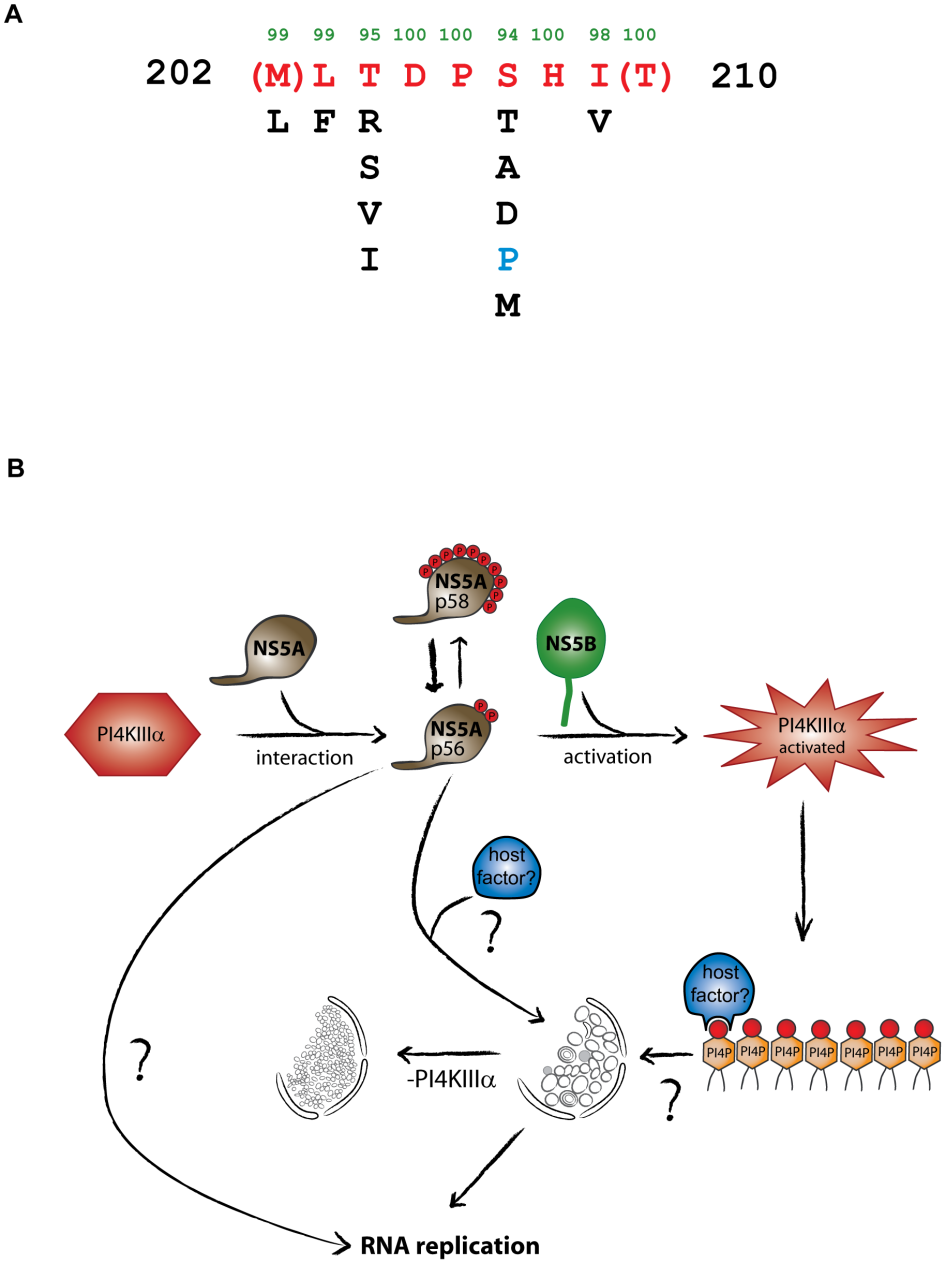
Model of the interplay between NS5A and PI4KIIIα. A: Consensus sequence (red) of the PI4KIIIα interaction site (PFIS) derived from 672 NS5A sequences of all genotypes in the Los Alamos HCV database (http://hcv.lanl.gov). Green numbers in the top line refer to the degree of conservation (rounded). Numbers on the left and right refer to the positions of the flanking amino acids within NS5A. Variations from the consensus are listed according to their frequency. A proline found in the JFH-1 PFIS is marked in blue. Variants found only once are not shown. B: NS5A (light brown) interacts with PI4KIIIα (red). This interaction regulates NS5A phosphorylation status directly or indirectly. Active kinase promotes NS5A p56 formation, a fraction of which is hyperphosphorylated resulting in p58. P56 might positively influence viral RNA replication either directly or by affecting the morphology of the replication sites, for which additional host factors are probably required. PI4KIIIα interaction with NS5A and NS5B is required to trigger lipid kinase activity. This leads to formation of new PI4P pools, presumably involved in membranous web morphology.

Our initial deletion analysis identified aa 187–213 of NS5A to be involved in PI4KIIIα binding (PBR), which could be narrowed down to a sequence of 7–9 aa (PFIS) crucial for PI4KIIIα interaction (Fig. 10A). The PFIS encompasses the very C-terminus of NS5A D1 (aa 202–210) and is highly conserved among all HCV genotypes, in line with the essential function of PI4KIIIα in HCV replication and with previous mapping studies [11], [61]. No function has been assigned yet to this region [4] and, unfortunately, this motif is not included in published crystal structures of NS5A D1 [33], [62]. However, a very recent study suggests that the PFIS partially overlaps with a region adopting an α-helical structure, which might be induced upon interaction with other proteins [63]. It also seems likely that the interaction of NS5A with PI4KIIIα is not restricted to the PFIS since almost all mutations in the PBR affected binding to PI4KIIIα to various extents and NS5A lacking the entire PBR or even D1 retained some PI4KIIIα binding (Fig. 1, ΔS3 and ΔD1). Our results furthermore strongly indicate that the interaction with NS5B is also essential for functional PI4KIIIα interaction, since expression of an NS3 to NS5A polyprotein was not capable of activating PI4KIIIα. Still, according to our data, the PFIS is important for PI4KIIIα binding and indispensable for the activation of the lipid kinase activity. All triple alanine mutations in this region had almost identical phenotypes, very similar to PI4KIIIα knockdown (Table 1), correlated with a strong impairment in PI4KIIIα binding and blocked HCV replication. This correlation argued for a replication defect mediated by interference with functional NS5A-PI4KIIIα interaction, which was furthermore supported by the partial rescue of RNA replication of PFIS mutant PPH upon PI4KIIIα overexpression, probably compensating the reduced binding efficiency. Replication defects of other PFIS mutants (MLT, TDP and HIT) were not compensated by PI4KIIIα overexpression, suggesting that the functional interaction of these NS5A mutants with PI4KIIIα was more severely impaired as for mutant PPH. Indeed, the PPH mutant was slightly less impaired in PI4P induction (Fig. 4C), induced MW clusters in a lower number of cells (Fig. 4B) and had a slightly reduced p58/p56 ratio (Fig. 6) compared to the other replication dead PFIS mutants. Alternatively, mutants MLT, TDP and HIT could require higher PI4KIIIα expression levels to compensate the functional interaction defects, which cannot be achieved by our lentiviral transduction system. We furthermore cannot rule out that mutations in the highly conserved PFIS motif might affect other important functions of NS5A independent from PI4KIIIα, like RNA binding, dimerization etc. and therefore cannot be rescued solely by PI4KIIIα overexpression.

M202A and T210A conferred intermediate phenotypes to the adjacent triple alanine mutants (RSM and TAE, respectively, Table 1) and therefore seem important, however not essential for the NS5A-PI4KIIIα interaction. Other mutations outside the PFIS were less consistent in their phenotypes by interfering with PI4KIIIα binding and PI4P synthesis only slightly, but not affecting RNA replication or p58/p56 ratios (e.g. mutants CEP, PEP and PDA; Table 1). This limited correlation argues for a complex interaction and suggests some functional flexibility until the defects fall below a threshold, as in case of the PFIS mutants. This flexibility was also indicated by the lack of strong phenotypes in case of the single mutants within the PFIS, despite the high degree of conservation at these positions (T204A and T210A, Fig. S7, Fig. 10A). Interestingly, alterations of D205 have even been identified as replication enhancing adaptive mutations in Con1 [46], [52], although this residue is invariant in natural isolates.

### Possible mechanisms governing NS5A phosphorylation by PI4KIIIα

The most striking and surprising phenotype observed for the PFIS mutants was the increase in p58/p56 ratios. Impairment of hyperphosphorylation is a much more common phenotype, which has been described upon sole expression of NS5A [38], [39], [50], drug treatment [43], [53], [64] and for many mutants [46], [48], [50]. Loss of hyperphosphorylation is often associated with abrogation of replication by general disturbance of the NS5A structure, as observed for mutations in the N-terminal amphipathic helix [31], [65] or in NS3, 4A and 4B [37], [38], [66]. To our knowledge, triple alanine mutants of the PFIS are the first NS5A variants reported with increased p58 levels as compared to wt NS5A. This phenotype strongly argues against a general impairment of NS5A structure of the PFIS mutants and for a specific effect mediated by PI4KIIIα. Importantly, the same phenotype was observed upon knockdown of PI4KIIIα, whereas overexpression of an active PI4KIIIα resulted in a reduced p58/p56 ratio. Collectively, these data clearly point to a specific regulation of NS5A phosphorylation by PI4KIIIα in a way favoring p56 synthesis or suppressing hyperphosphorylation, congruent with the essential role of PI4KIIIα in HCV RNA replication. Several mechanisms could be envisaged mediating this phenotype.

First, NS5A phosphorylation might be altered upon changes in the lipid environment of the viral replication sites induced by the activation of PI4KIIIα, thereby sequestering p56 and preventing p58 synthesis. This scenario seems rather unlikely, since the majority of viral nonstructural proteins (>95%) is not shielded by the MW, as judged by the accessibility to proteases [7], [8]. In addition, p58 synthesis starts immediately after polyprotein processing and is completed within 20–60 minutes [40], [67], which might not be compatible with the time lines required for sequestration in membrane rearrangements. However, a concise analysis of the kinetics of p56 and p58 synthesis of different virus isolates in presence and absence of PI4KIIIα will be required to shed light on this important issue. It will also be very interesting to analyze whether p58/p56 ratios differ in total cellular lysates versus protease resistant fractions for wildtype and PFIS mutants, although these experiments are technically challenging due to the low amounts of protease resistant nonstructural proteins.

Second, enhanced PI4P levels might recruit kinases or phosphatases involved in the regulation of p58/p56 ratios. Up to now only a few proteins have been found to specifically bind PI4P (reviewed in [16]), which might be involved in the recruitment of enzymes modulating NS5A phosphorylation. Among those, oxysterol-binding protein 1 (OSBP) and ceramide transfer protein (CERT) have already been shown to be involved in the secretion of virions [68], [69]. However, recent proteomic data on the composition of the HCV replication sites neither identified significant accumulations of any of these proteins nor kinases or phosphatases which might be involved in regulation of NS5A phosphorylation [70]. Still, a more comprehensive proteomic study involving conditions of PI4KIIIα knockdown might help to identify host factors involved in NS5A phosphorylation recruited by PI4P or by PI4KIIIα.

Third, PI4KIIIα might shield NS5A from interaction with kinases such as CKIα or Plk1 [44], [45], thereby preventing hyperphosphorylation. Interestingly, the PFIS motif (aa 202–210) identified in our study to be essential for PI4KIIIα interaction is adjacent to the serine cluster critical for p58 synthesis (aa 222–235). Therefore, binding of PI4KIIIα to the PFIS might passively prevent the access of enzymes promoting hyperphosphorylation and/or block the phosphorylation sites, thereby favoring p56 synthesis. However, this hypothesis is not in line with the requirement for PI4KIIIα enzymatic activity to reduce p58/p56 ratios in case of JFH-1 and Con1 wt.

Fourth, PI4KIIIα might directly phosphorylate NS5A, thereby blocking the interaction with CKIα/Plk1 and preventing hyperphosphorylation. Although no protein kinase activity has been demonstrated for PI4KIIIα yet, the closely related isoform PI4KIIIβ has been shown to autophosphorylate *in vitro*, thereby inhibiting lipid kinase activity [71]. A more distinctly related class of PI kinases, phosphoinositide 3-kinases (PI3K), also contain lipid kinase as well as protein kinase activities, which are both essential for their function [72], [73], and share structural similarities to protein kinases [74]. Interestingly, phosphatidylinositol 3-kinase-related kinases (PIKKs) like mTOR comprise a related family of Ser/Thr kinases without lipid kinase activity [75]. All PI3Ks, PIKKs, PI4KIIIα and PI4KIIIβ are sensitive to wortmannin [13], [76], arguing for some structural relatedness in the active center and leaving the possibility that PI4KIIIα might give rise to protein phosphorylation as well. Preliminary data using PI4KIIIα from commercial sources (Invitrogen, Millipore) and proteins purified by ourselves indeed revealed some protein kinase activity *in vitro* (D. Radujkovic, V. Lohmann, unpublished data), but these preparations were not of sufficient purity to draw firm conclusions, as judged by the presence of contaminating protein kinase activity also in preparations of inactive kinase mutants. Therefore, a comprehensive analysis of PI4KIIIα protein kinase activity *in vitro* including inactive mutants and a thorough analysis of substrate requirements are needed to verify a direct role of PI4KIIIα in NS5A phosphorylation.

### Impact of PI4KIIIα on NS5A stability

Several experimental results pointed to a role of PI4KIIIα in regulating the abundance of NS5A, irrespective of the phosphorylation state (Fig. 7). First, AL-9 treatment generally and dose dependently enhanced the abundance of NS5A (Con1 and JFH-1). Second, overexpression of PI4KIIIα wt reproducibly reduced the abundance of NS5A (Con1), whereas an inactive PI4KIIIα mutant rather seemed to increase the apparent expression levels of NS5A. Third, NS5A PFIS mutants, devoid of functional PI4KIIIα interaction, seemed to be expressed to higher levels than the wildtype counterparts (preferentially Con1). These results indicate that active PI4KIIIα probably reduces the stability of NS5A, which might be important for the regulation of RNA replication. We have not yet analyzed, whether PI4KIIIα also affects the abundance of other nonstructural proteins, but this important question will be addressed in future experiments. However, it is currently unlikely that the impact of PI4KIIIα on NS5A stability/abundance is the main role of this host factor in regulating HCV RNA synthesis, since this phenotype was less pronounced for JFH-1 NS5A (apart from AL-9 treatment) and not found at all upon silencing of PI4KIIIα. Still, a regulatory role of PI4KIIIα in the half-life and turnover of HCV nonstructural proteins and/or replication complexes is an interesting hypothesis, which needs to addressed in more detail in subsequent studies.

### Regulation of PI4KIIIα lipid kinase activity

Previous studies have shown that NS5A was critically involved in the activation of PI4KIIIα [11], [29], which was confirmed by the failure of the PFIS mutants to give rise to PI4P induction. Interestingly, an additional mutant (LPC), which was severely impaired in RNA replication, most likely by a mechanism independent of PI4KIIIα, failed to activate PI4KIIIα, suggesting that activation of PI4KIIIα might be disturbed by a number of pleiotropic mutations interfering with the overall integrity of NS5A. Our new data furthermore indicate that expression of NS5A or NS3-5A is not sufficient for PI4KIIIα activation (Fig. 8C), suggesting that a complex of NS5A and NS5B is involved in the regulation of PI4KIIIα. This is in line with previous data identifying an interaction of PI4KIIIα with both viral proteins [11], but in striking contrast to results showing PI4P induction and redistribution by the sole expression of NS5A [11], [29]. Here, we used lentiviral transduction to reach physiological expression levels comparable to replicon cells, whereas former studies relied on T7-based expression [11] or tet-inducible expression in osteosarcoma cells [29], giving rise to much higher protein abundance, which might explain this discrepancy. Although NS5A was shown to be sufficient for activation of PI4KIIIα *in vitro* [11], [29], we found no evidence for a stronger activation of PI4KIIIα *in vitro* by simultaneous addition of NS5A and NS5B purified from *E. coli* (D. Radujkovic, V. Lohmann, unpublished data). This observation points to a complex regulation, probably depending on the phosphorylation state of NS5A or on the presence of additional host factors like protein kinase D, which activates the related PI4KIIIβ by phosphorylation [77]. Indeed, protein kinases seem to be physically associated even with purified NS5A [47], [78], which could be involved in the activation of PI4KIIIα. Anyhow, a thorough analysis *in vitro* and in cell culture will be required to unravel the intricate determinants of PI4KIIIα activation by HCV and the role of NS5A and NS5B in this process.

### Enhanced global PI4P abundance is not essential for HCV RNA replication

Generally, little is known about the regulation of PI4KIIIα [13], but virus-induced increases in PI4P levels are not unique to HCV [27]. Enteroviruses activate PI4KIIIβ by a yet to be defined mechanism, generating a binding platform for the viral polymerase important for RNA replication [22]. In case of HCV, a more recent study indicates that HCV not only activates PI4KIIIα but also prevents transport of PI4P to the plasma membrane by an unknown mechanism and both mechanisms seem to contribute to the intracellular accumulation of PI4P [15]. However, our novel data suggest that the gross changes in PI4P levels are not essential for HCV RNA replication but might rather be a consequence of viral replication/protein expression. This assumption is based on the huge variations in PI4P levels observed in HCV positive cells, particularly since some of the mutants gave rise to significantly reduced PI4P levels but were not impaired in RNA replication at all (e.g. mutCEP and PDA, Table 1). Furthermore, the transcomplementation analysis revealed a number of cells with detectable rescue of HCV replication but lacking detectable alterations in intracellular PI4P levels and substantial colocalization of PI4P and NS5A (Fig. 9C, D). Finally, elevated levels of PI4P alone were not capable of rescuing the defects of the PFIS mutants, as demonstrated by the lack of replication of Con1 repHIT in JFH-1 wt replicon cells, although this phenotype might be due to additional defects in NS5A caused by the HIT mutations. However, previous data demonstrated that only 20% of intracellular PI4P colocalized with NS5A [11] and that expression of the PI4P phosphatase Sac1 had only a limited impact on HCV replication [22], [24]. Collectively, these results argue against a distinct role of globally enhanced intracellular PI4P concentrations in viral RNA replication. We therefore speculate that activation of PI4KIIIα by HCV might be required to generate locally enhanced PI4P amounts at the site of replication, resulting in global disturbance of PI4P metabolism as a collateral effect. Alternatively, the new PI4P pools might be required at a later step of the viral replication cycle like assembly or release of virions, as suggested by involvement of PI4P binding proteins CERT and OSBP in HCV secretion [68], [69]. Enhancement of intracellular and depletion of plasma membrane PI4P pools might furthermore impact on signaling events and/or contribute to viral pathogenesis *in vivo*. However, these important questions can only be addressed in adequate animal models, which will hopefully be developed in the future.

### Impact of PI4KIIIα on MW formation

Accumulating evidence suggests that PI4KIIIα is important for the generation of the MW. Silencing and inhibition of PI4KIIIα was associated with clustered web structures in IF [11], [15], [18], which contained smaller DMVs and lacked MMVs [11]. These phenotypes have been attributed to reduced PI4P levels in absence of PI4KIIIα activity. Clustered distribution of NS5A in IF as well as more distinct accumulation of vesicles with smaller DMVs was also observed upon expression of the mutant NS3-5B proteins, confirming that these phenotypes are linked to PI4KIIIα. However, mutHIT, being devoid of PI4KIIIα activation, induced even smaller DMVs than silencing of PI4KIIIα, although PI4P levels were comparable and not significantly different for both conditions (Table 1). These data argue for PI4KIIIα-mediated mechanisms modulating the morphology of the HCV replication sites independent from PI4P and probably mediated by NS5A phosphorylation. Interestingly, a very recent study demonstrated that ectopic expression of NS5A induced the formation of vesicles containing several lipid bilayers and occasionally vesicles containing a pair of membranes morphologically identical to DMVs, whereas sole expression of NS3/4A, NS4B and NS5B generated only single membrane vesicles, emphasizing the role of NS5A as a key regulator of MW morphogenesis [79]. Importantly, expression of NS3-5A did not significantly induce PI4P synthesis in our study, but generated DMVs and some more tubular double membrane structures with an average diameter similar to NS3-5B wt [79] and larger than mutHIT or silencing of PI4KIIIα. It is therefore tempting to speculate that changes of NS5A phosphorylation rather than PI4P induced by PI4KIIIα are involved in modulation of the MW, since mutHIT also generated a stronger change in p58/p56 ratio than PI4KIIIα silencing (1.5 vs. 1.0 respectively, compared to 0.6 for wt). The small DMV size and the lack of MMVs observed upon silencing of PI4KIIIα and expression of mutHIT might therefore at least in part be due to higher p58/p56 ratios associated with these conditions (Table 1), probably mediated by a host factor. Interestingly, hVAP-A, a cellular protein involved in vesicle trafficking and essential for HCV replication [80], [81], has been shown to bind p56 and not p58 [49] and thereby represents a promising candidate factor for phosphorylation dependent regulation of MW morphology, particularly since hVAP-A is enriched in viral replication sites [81]. However, a more detailed ultrastructural and biochemical analysis of PI4P induction and NS5A phosphorylation will be required to further dissect the contribution of both factors to the composition of HCV-induced membrane alterations and to clarify the role of other cellular proteins.

### Model of PI4KIIIα function in HCV RNA replication

Our novel finding of PI4KIIIα modulating NS5A phosphorylation adds an additional layer of complexity to the mechanism of action of this essential host factor of HCV RNA replication. However, we favor a model in which interaction of PI4KIIIα with NS5A p56 triggers a phosphorylation event preventing further hyperphosphorylation (Fig. 10B). P56 or a distinct ratio of p58/p56 is then involved in the morphogenesis of the MW, probably by recruiting a host factor like hVAP-A. In addition, p58/p56 ratios might also have a more direct impact on viral replication, e.g. by regulating RNA synthesis. Interaction of PI4KIIIα with NS5A in concerted action with NS5B furthermore activates the lipid kinase, resulting in increased intracellular PI4P levels. Activation of PI4KIIIα might again be supported by a host factor, e.g. a protein kinase, recruited by NS5A or NS5B. Locally increased PI4P levels might be involved in MW morphology as well, but our data argue against a vital role of globally increased PI4P concentrations. In contrast, we speculate that the activation of the lipid kinase is rather a consequence of the interaction with the viral proteins and might impact on viral pathogenesis.

Conclusively, our study provides important novel insights into the role of PI4KIIIα in HCV RNA replication. Still, we are far from a comprehensive view on the entire mechanism of action, in particular regarding the role of NS5B in its activation, the determinants of NS5A phosphorylation and further host factors involved. However, unraveling the complexities of HCV-PI4KIIIα interactions will be of central importance for our understanding of the viral replication machinery and might also help to shed light on the distinct cellular functions of PI4KIIIα, which are barely understood.

## Materials and Methods

### Cell lines

The Huh-7 cell clone Huh7-Lunet, highly permissive for HCV RNA replication [82] was used for electroporation assays. Huh7 cell lines bearing subgenomic replicons of either JFH-1 isolate [56] or adapted Con1 ET [7] have been described recently. Huh7-Lunet T7 cells [83] were used for transient expression of plasmids coding for HCV proteins analyzed in immunofluorescence and immunoprecipitation assays. Huh7-Lunet cells served for the generation of cell lines stably overexpressing HCV proteins. Following cell lines have been created by lentiviral transduction as described elsewhere [83] using lentiviral pWPI plasmid vectors under selection of blasticidin or zeocin: Huh7-Lunet NS3-5A-JFH1, Huh7-Lunet NS3-5A-Con1ET, Huh7-Lunet NS5A-JFH1 and Huh7-Lunet NS5A-Con1ET were used in transcomplementation assays. Huh7-Lunet T7 cells stably overexpressing either wildtype PI4KIIIα or inactive D1957A mutant were established by lentiviral transduction using pWPI-HA-PI4KIIIα or pWPI-HA-PI4KIIIαD1957A, respectively [11]. Cells with stable knockdown of PI4KIIIα (shPI4KIIIα) or non-targeting control cells (shNT) were prepared according to a recently published protocol [84]. The shRNA targeting sequences were 5′-CAG TGG AAG GAC AAC GTG-3′ (PI4KIIIα) and 5′-TCT CGC TTG GGC GAG AGT AAG-3′ (NT). Huh7-Lunet T7 cells with stable PI4KIIIα knockdown expressing an shRNA escape variant of PI4KIIIα (si+esc) have been generated by lentiviral transduction using pWPI HA-PI4KIIIα-shEsc.

### Plasmid constructs

The lentiviral vector pWPI-BLR [83] has been used for cloning of following plasmids for the generation of stable cell lines under blasticidin selection: pWPI-NS3-5AJFH1, pWPI-NS5AJFH1, pWPI-NS3-5ACon1ET and pWPI-NS5ACon1ET. The gene encoding the >230 kDa full-length PI4KIIIα isoform 2 was originally obtained from Kazusa DNA Research Institute, Chiba, Japan (product ID FXC00322, corresponding to GenBank accession number AB384703, numberings refer to this GeneBank ID). For construction of pWPI-PI4KIIIα-shEsc, a synthetic gene fragment was obtained from GeneArt (Regensburg, Germany), which comprised the region targeted by the different shRNAs and exhibited silent mutations in shRNA-targeting sequences. The silent escape mutations were based on the following nucleotide exchanges: g4644a, g4650a, c4653t, c4656t, g4659c, g5085a, c5088t, c5091a, c5097t, c4437g, t4441a, c4442g, t4443c, g4446a, a4449g, c4452g. Detailed cloning protocols for lentiviral constructs can be obtained upon request.

All amino acid and nucleotide numbers refer to the position of the corresponding amino acid in the complete HCV genomes or in the NS5A protein of JFH1 and Con1 (GenBank accession no. AB047639 and AJ238799, respectively). PTM vectors allowing expression of the HCV nonstructural proteins NS3 to 5B or individual HCV NS proteins as well as the HA-tagged PI4KIIIα have been described recently [11], [83]. PTMNS3-5B served as vector plasmid for internal NS5A deletions as well as for triple alanine NS5A mutations. PTMNS3-5B/NS5AΔD1 was described recently [83]. Following subdeletions of NS5A within the context of the polyprotein were generated by three-fragment ligation by using the BstXI/SpeI digested PCR fragments and vector fragments obtained by restriction with BstXI and SpeI or SpeI and RsrII. Overlap-PCR were performed using primers F_MfeI and R_RsrII_JFH as well as primers indicated in table S1 spanning the crossover sites. PTM NS3-5B/5AΔS1 contains an in frame deletion of aa2002–2068 established by overlap-PCR using primers F_del5AD1a and R_del5AD1a. PTM NS3-5B/5AΔS2 contains an in frame deletion of aa2069–2126 established by overlap-PCR primers F_del5AD1b and R_del5AD1b. PTM NS3-5B/5AΔS3 contains an in frame deletion of aa2127–2190 within NS5A domain I established by overlap-PCR using primers F_BssHII and R_SacI as well as the primers F2_Del_alles_small and R124_Del_alles spanning the crossover site. Plasmid was generated by three-fragment ligation by using the BssHII/SacI digested PCR fragment and vector fragments obtained by restriction with BssHII and SpeI or SpeI and SacI. Further subdeletions ΔS3A (Δaa2127–2162), ΔS3B (Δaa2163–2175) and ΔS3C (Δaa2176–2189) were established by overlap-PCR using the primers F_BssHII and R_SacI as well as the primers for the according single crossover sites which can be found in table S1. Plasmids were generated by three-fragment ligation by using the BstXI/RsrII digested PCR fragment and vector fragments obtained by restriction with BstXI and SpeI or SpeI and RsrII. Triple alanine mutants pTMNS3-5B/NS5AmutXXX (XXX refers to SQL, LPC, CEP, PEP, PDA, ADV, VLR, RSM, MLT, TDP, PPH, HIT, TAE, ETA) as well as pTMNS3-5B containing single amino acids substitutions in NS5A (S201A, T204A, T210A and T213A) were created by overlap-PCR using the primers F_BssHII and R_SacI as well as the primers for the according single crossover sites which can be found in table S1. Plasmids were generated by three-fragment ligation by using the BssHII/SacI digested PCR fragment and vector fragments obtained by restriction with BssHII and SpeI or SpeI and SacI. According plasmid constructs of genotype 1b (Con1 or Con1ET) containing the triple alanine mutation mutHIT have been created as well by overlap-PCR and fragment substitutions: pTMNS3-5B/NS5AmutHIT Con1/Con1ET were created by overlap-PCR using the primers S/1B/6803 and A/1B/7263 as well as the primers for the according single crossover sites which can be found in table S1. Plasmids were generated by fragment ligation by using the EcoRI/XhoI digested PCR fragment and vector fragment (either from pTMNS3-5B Con1 or Con1ET, respectively) obtained by restriction with EcoRI and XhoI.

Subgenomic reporter replicons have been have been described elsewhere: the monocistronic genotype 2a (JFH-1) reporter pFKI_389_-Lucubi-NS3-3′/JFH1wt_δg [85]; the bicistronic adapted genotype 1b (Con1ET) reporter pFKI_341_-Luc-EI/NS3-3′/Con1ET_δg [52]; the luciferase reporter replicon of genotype 2a with an additional eGFP insertion in NS5A domain III pFKI_389_-Luc-NS3-3′/δg/JFH1-5A-eGFP [58]. Triple alanine mutations within pFKI_389_-Lucubi-NS3-3′/JFH1mutXXX_δg monocistronic replicons were created by replacing the NsiI/HindIII fragment of pFKI_389_-Lucubi-NS3-3′/JFH1wt_δg with the NsiI/HindIII fragment of the according pTMNS3-5B/NS5AmutXXX plasmid. PFKI_341_-PILuc-NS3-3′ET/NS5AmutHIT_δg bicistronic replicon was created by replacing the MluI/XhoI fragment of PFKI_341_-PILuc-NS3-3′ET_δg with the MluI/XhoI fragment of the pTMNS3-5B/NS5AmutHIT Con1ET plasmid. The triple alanine mutation mutHIT within the reporter replicon pFKI_389_-Luc-NS3-3′/δg/JFH1-NS5AmutHIT-eGFP containing an eGFP insertion in NS5A domain III was cloned by three fragment ligation by using the AgeI/SacI vector fragment of pFKI_389_-Luc-NS3-3′/δg/JFH1-5A-eGFP and fragments obtained from AgeI/NsiI cleavage of pFKI_389_-Luc-NS3-3′/δg/JFH1-5A-eGFP or NsiI/SacI cleavage of pTMNS3-5B. All PCR-derived sequences were confirmed by sequencing (GATC, Konstanz, Germany).

### Transient HCV replication and trans-complementation assay

Transient HCV RNA replication assays were performed as described previously [86]. In brief, replicon encoding plasmid DNA harboring hepatitis delta virus ribozymes were restricted with MluI (JFH1) or Spe I (Con1) prior to *in vitro* transcription. Ten µg of run-off transcripts were used for electroporation of 4×10^6^ Huh7-Lunet cells or Huh7-Lunet cells overexpressing PI4KIIIα, that were resuspended in 12 ml culture. Two ml aliquots were seeded per well of a 6-well plate and replication was determined by measuring luciferase activity in case of genomes containing the luciferase reporter gene at 4 h, 24 h, 48 h and 72 h post electroporation. Since luciferase activity measurable 4 h post transfection is derived from transfected input RNA, these values were used to normalize for transfection efficiency. For transcomplementation assays, Huh7-Lunet cells containing a stably selected subgenomic replicon or stably overexpressing NS3 to 5B or NS5A were transfected with 10 µg of replicon RNA that contain a luciferase reporter gene and are derived from pFKI plasmid DNA. Electroporated cells were seeded as described above and values obtained 4 h post electroporation were used to determine the transfection efficiency.

To assess replication efficiency of different replicon constructs by direct measurement of viral RNA, 0.25 µg of replicon RNA was electroporated into Huh7-Lunet cells and HCV RNA was quantified by RT-PCR as described recently [87].In brief, viral RNA was isolated from transfected cells using the Nucleo Spin RNAII kit (Macherey-Nagel, Düren, Germany) as recommended by the manufacturer. 200 ng of RNA sample was used for quantitative RT-PCR analysis using an ABI PRISM 7000 sequence detector system (Applied Biosystems, Foster City, CA). HCV-specific RT-PCRs were conducted in triplicates with the One Step RT-PCR kit (QIAGEN, Hilden, Germany) using the following JFH1-specific probe (TIB Molbiol, Berlin, Germany) and primers (MWG-Biotech, Martinsried, Germany): A-195, 5′-6-carboxyfluorescein-AAA GGA CCC AGT CTT CCC GGC AAT T-tetrachloro-6-carboxyfluorescein-3′; S-146, 5′-TCT GCG GAA CCG GTG AGT A-3′; and A-219, 5′-GGG CAT AGA GTG GGT TTA TCC A-3′. The amount of HCV RNA was calculated by comparison to serially diluted in vitro transcripts.

### Immunofluorescence analysis and PI4P quantitation

For overexpression of HCV proteins, Huh7-Lunet T7 cells were transfected with Effectene (Qiagen, Hilden Germany) transfection reagent according to manufacturer's instructions and fixed 24 h post-transfection. HCV replicons were transfected by electroporation into Huh7-Lunet cells and fixed after 48 hours. Immunofluorescence protocol was performed as described elsewhere [11]. In brief: Cells were fixed in 4% PFA for 20 min and permeabilized with 50 µg/ml Digitonin for another 15 min. Primary antibodies were incubated in 3% BSA for 1 h at RT. NS5A was detected by using either NS5A-specific monoclonal mouse antibody (9E10, generous gift from Charles M Rice) with a final concentration of 3 µg/ml or using a polyclonal NS5A rabbit antiserum (#4952, [83]) at a dilution of 1∶300. NS3, NS4B and NS5B were detected by polyclonal antisera previously described [83]. PI4P was stained using monoclonal mouse IgM anti-P4P antibody (Echelon, Z-P004) with a final concentration of 5 µg/ml. eGFP signals were enhanced by a polyclonal rabbit anti-GFP antibody (Abcam, ab290) with a dilution of 1∶200. Alexa 488 or 546 conjugated secondary antibodies (Invitrogen, Molecular Probes) were incubated in 3% BSA for 45 min at RT with a dilution of 1∶1000. Nuclei were stained using DAPI for 1 min. at a dilution of 1∶4000. Cells were mounted with Fluoromount G (Southern Biotechnology Associates, Birmingham, USA) and pictures were acquired with a Nikon C1Si spectral imaging confocal laser scanning system on a Nikon Ti fully automated inverted microscope equipped with 60× objective. For PI4P quantitation, a 40× objective was used and z projections of confocal z stacks were generated with the “sum slices” option of ImageJ. After thresholding of the signal intensity of PI4P staining, PI4P amount of 35 different cells was determined by defining cell areas and taking the IntDen-value obtained with the “analyze particles” functions of Image J. Quantitation of membranous web phenotypes was based on a qualitative visual judgment of 350 randomly chosen NS5A positive cells, which were either assigned to the wt or cluster phenotype, based on the representative images shown in Fig. 4A. The percentage relies on the relative number of cells grouped into the wt or cluster phenotype for each construct.

### Electron microscopy

For overexpression of HCV proteins, Huh7-Lunet T7 cells were transfected with TransIT-LT1 (Mirus, Madison USA) transfection reagent according to manufacturer's instructions and fixed 24 hours post transfection. HCV replicons were transfected by electroporation into Huh7-Lunet cells and fixed after 48 hours. For fixing, cells were washed 3 times with 1× PBS and fixed for 30 min with pre-warmed 2.5% glutaraldehyde in 50 mM sodium cacodylate buffer (pH 7.2) containing 1 M KCl, 0.1 M MgCl_2_, 0.1 M CaCl_2_ and 2% sucrose. Cells were washed thoroughly 5 times with 50 mM cacodylate buffer and post-fixed on ice in the dark with 2% OsO_4_ in 50 mM cacodylate buffer for 40 min. Cells were washed with H_2_O overnight, treated with 0.5% uranyl acetate in H_2_O for 30 min, rinsed thoroughly with H_2_O and dehydrated in a graded ethanol series at RT (40%, 50%, 60%, 70% and 80%) for 5 min each and 95% and 100% for 20 min each. Cells were immersed in 100% propilene oxid and immediately embedded in an Araldite-Epon mixture (Araldite 502/Embed 812 Kit; Electron Microscopy Sciences). After polymerization at 60°C for 2 days coverslips were removed and the embedded cell monolayers were sectioned using a Leica Ultracut UCT microtome and a diamond knife. Sections with a thickness of 65 nm were counter-stained with 3% uranyl acetate in 70% methanol for 5 min and 2% lead citrate in H_2_O for 2 min, and examined with the transmission electron microscope Philips CM120 TEM (Biotwin, 120 kV).

### Metabolic radiolabeling of proteins, AL-9 treatment, immunoprecipitation and Western blotting

For metabolic labeling a total of 4×10^5^ Huh7-Lunet cells constitutively expressing the T7 RNA-polymerase (Huh7-Lunet T7) were seeded in each well of a 6-well cell culture plate in complete DMEM. One day later, cells were transfected with pTM vectors [88] allowing protein expression under transcriptional control of the T7 promoter. 2 µg of pTM vectors supporting expression of the HCV nonstructural proteins NS3 to NS5B with deletions of NS5A subdomains or mutations within NS5A were cotransfected with 2 µg of either pTM HA-PI4KIIIα or an empty pTM vector (mock) by using Lipofectamine2000 (Invitrogen) according to the instructions of the manufacturer. After 7 h, cells were washed with methionine/cysteine-free medium and incubated in this medium for 1 h. For radiolabeling cells were incubated for 16 h in 1 ml methionine/cysteine-free medium, supplemented with 10 mM glutamine, 10 mM Hepes, and 100 µCi/ml of Express Protein labeling mix (Perkin Elmer, Boston). In assays using AL-9 (generous gift from R. De Francesco, P. Neddermann and F. Peri, Milan, Italy), the drug was diluted in DMSO to indicated concentrations and supplemented to the methionine/cysteine-free medium and incubated overnight. Cells were lysed by incubation of the cell pellets in NPB (50 mM Tris-Cl [pH 7.5], 150 mM NaCl, 1% Nonidet P-40, 1% sodium deoxycholate, 0.1% SDS, protease inhibitors) for 1 h on ice. Lysates were cleared by centrifugation at 14,000 g for 10 min at 4°C and used for immunoprecipitation with antibodies of the following specificities: NS5A of genotype 1a (H77) cross-reacting with Con1 and JFH-1 (sheep polyclonal, a generous gift of M. Harris, Leeds university, U.K.) (Macdonald et al., 2003); 7 µg of anti-HA tag (mouse H3663, Sigma). After 3 h incubation at 4°C immunocomplexes were captured by using protein-G-sepharose beads (Sigma) for an additional 3 h incubation at 4°C. Where indicated, complexes were treated after washing in buffer 3 (NEB) with 1 U CIP (NEB, #M0290S) for 1 h at 30°C. Immunocomplexes were dissolved in protein sample buffer, separated by 10% polyacrylamide-SDS gel electrophoresis and detected by autoradiography. Proteins were quantified by phosphoimaging using the Quantity One software (Bio-Rad, Munich). HA-PI4KIIIα binding to NS5A was determined by normalizing the amount of HA-PI4KIIIα co-precipitating with NS5A to the total amount of HA-PI4KIIIα determined by direct IP using anti-HA antibodies. This option was chosen since quantitation of total PI4KIIIα input levels by western blot was not reproducible due to variable and inconsistent transfer rates of the 240 kDa PI4KIIIα band. Data were furthermore not normalized to input NS5A levels due to a consistent 20–100fold molar excess of NS5A compared to HA-PI4KIIIα (data not shown).

For western blotting 1/10 cells of a T25 cell culture flask were denatured and heated in 2× Laemmli-buffer and loaded onto an 8% polyacrylamide-SDS gel. After separation and transfer to a PVDF membrane, immunoblotting was performed detecting NS5A using a monoclonal mouse antibody (9E10) at a concentration of 0.1 µg/ml and β-actin with a monoclonal mouse antibody (Sigma, A5441) and a dilution of 0.5 µg/ml. LI-COR secondary antibodies conjugated to IRDye were used 1∶10000. Bound antibodies were detected with the LI-COR Infrared Imaging System.

### Statistical analyses

General statistical analyses as indicated in the corresponding figures were performed using Microsoft Excel software. Correlations of experimental data were obtained by linear regression analysis using GraphPad Prism software.

## Supporting Information

Figure S1 **Impact of triple alanine mutations on HCV RNA replication.** A: Huh7-Lunet cells were transfected with luciferase reporter replicons bearing the indicated triple alanine substitutions. JFH-1 wt replicons (wt) and a mutant harboring a deletion within NS5B (ΔGDD) served as positive and negative controls, respectively. Total cellular RNA was extracted 4 h (light blue) or 72 h (dark blue) after transfection. HCV RNA was quantified by quantitative RT-PCR and is depicted as non-normalized HCV RNA copies per µg of total cellular RNA at the respective time points. B: Naïve Huh7-Lunet cells (control) or Huh7-Lunet cells stably overexpressing HA-tagged PI4KIIIα (HA-PI4KIIIα) were transfected with luciferase reporter replicons as described in the legend of Fig. 3D. Depicted are the normalized ratios of luciferase activity 48 h after transfection in HA-PI4KIIIα overexpressing cells compared to naïve Huh7-Lunet cells. C: Replication efficiency at 24 h relative to 4 h after transfection of a subset of triple alanine mutants in naïve Huh7-Lunet cells (Control, black bars) or in Huh7-Lunet cells overexpressing HA-PI4KIIIα (grey bars) as shown in Fig. 3D. JFH-1 wt replicons (wt, green bars) and a mutant harboring a deletion within NS5B (ΔGDD, red bars) served as positive and negative controls, respectively.
(TIF)

Figure S2 **Subcellular localization of NS3, NS4B and NS5B relative to NS5A.** Huh7-Lunet T7 cells with stable knockdown of PI4KIIIα (shPI4KIIIα) or control cells (shNT) were transfected with plasmids encoding the NS3 to NS5B polyprotein of genotype 2a (JFH-1) containing a wt sequence or the NS5A triple alanine mutant mutHIT or with empty plasmid (mock). 24 h post transfection NS5A (red) and an additional nonstructural protein (A: NS3, B: NS4B or C: NS5B, green), were detected with specific antibodies and nuclear DNA was stained with DAPI (blue). Note the punctuate staining pattern of NS3, NS4B, NS5A and NS5B in 3-5B wt transfected cells compared to formation of “clusters” in cells with stable knockdown of PI4KIIIα or expressing mutant polyproteins and the consistent colocalization of all nonstructural proteins for each experimental condition.
(TIF)

Figure S3 **Characterization of NS5A mutants not impaired in RNA replication.** Huh7-Lunet cells were transfected with subgenomic JFH-1 reporter replicons of either wt (wt), containing a deletion within NS5B (ΔGDD) or bearing indicated triple alanine substitutions. A: 48 h post transfection NS5A (red) or PI4P (green), respectively, was detected with specific antibodies and DAPI was used to stain nuclei (blue). B: Quantitative analysis of PI4P fluorescence intensity of cells as shown in panel A by ImageJ analysis (IntDen read-out). Error bars indicate the mean +/− SD of 50 NS5A positive cells analyzed per condition. Significance of increased PI4P levels relative to mock was measured by a paired t-test and is indicated. ***, p<0.001. The blue line points to the mean of PI4P IntDen values of untransfected cells (mock). C: Mean values and standard deviations of PI4P quantitation obtained from data of figure S3B, relative to mock transfected cells. D: Cells were fixed and prepared for EM analysis 48 h post transfection. Consecutive enlargements of the boxed areas are shown from left to right. For details see legend to fig. 4. E: Average diameter of DMVs detected in the indicated replicon cells 48 h after transfection of replicon RNA. Error bars indicate the mean +/− SD of seventy vesicles. Significance of differences in DMV sizes was measured by a paired t-test and is indicated ***, p<0.001. F: Proteins were radiolabeled 48 h after transfection and cell lysates were subjected to immunoprecipitation using NS5A-specific antibodies. Samples were analyzed by SDS-PAGE and autoradiography.
(TIF)

Figure S4 **Phosphatase treatment of triple alanine mutants.** Huh7-Lunet T7 cells were transfected with plasmids encoding the NS3 to NS5B polyprotein of genotype 2a (JFH-1) containing a wt sequence or triple alanine mutations as indicated or with empty plasmid (mock). Newly synthesized proteins were radiolabeled and cell lysates subjected to immunoprecipitations using NS5A-specific antibodies. After immunoprecipation samples were treated with calf intestine phosphatase (CIP) or mock treated as indicated and analyzed by SDS-PAGE and autoradiography.
(TIF)

Figure S5 **Specific inhibition of PI4KIIIα activity by AL-9 does not affect NS5A- PI4KIIIα binding.** A: Huh7-Lunet T7 cells were transfected with plasmids encoding the NS3 to NS5B polyprotein of genotype 2a (JFH-1) and HA-tagged PI4KIIIα (PI4KIIIα). Starting at 7 h post transfection, cells were incubated with 5 µM of AL-9 or DMSO. Newly synthesized proteins were radiolabeled and cell lysates subjected to immunoprecipitation using NS5A or HA-specific antibodies. B: Quantitative analysis of PI4KIIIα pull-down efficiency. Experiments as shown in panel A were quantified by phosphoimaging. Coprecipitation efficiency was normalized to the total amounts of HA-PI4KIIIα and calculated relative to PI4KIIIα pull-down by NS5A. Error bars indicate mean values +/− SD of two independent experiments analyzed in duplicates.
(TIF)

Figure S6 **PI4KIIIα overexpression modulates phosphorylation of NS5A mutants.** A: Huh7-Lunet T7 cells were cotransfected with plasmids encoding the NS3 to NS5B polyprotein of genotype 2a (JFH-1) containing triple alanine mutations in NS5A domain I as indicated and HA-tagged PI4KIIIα (HA-PI4K). Newly synthesized proteins were radiolabeled and cell lysates subjected to immunoprecipitations using NS5A-specific antibodies. Samples were analyzed by 10% SDS-PAGE and autoradiography. B: Quantitative analysis of the NS5A p58/p56 ratio. Bands corresponding to NS5A p58 and p56, respectively, as shown in panel A were individually quantified by phosphoimaging to obtain a p58/p56 ratio. Error bars indicate mean values +/− SD of two independent experiments analyzed in duplicates. Significances were compared to the wt polyprotein and calculated by paired t-tests.*, p<0.05; **, p<0.01; ***, p<0.001. n.d. not determined due to insufficient resolution of p56 and p58.
(TIF)

Figure S7 **Analysis of potential NS5A phosphorylation sites within the PI4KIIIα binding motif.** A: Scheme depicting possible NS5A phosphorylation sites within the PI4KIIIα binding motif that were individually mutated to alanine within the context of subgenomic replicons or NS3 to NS5B polyprotein expression plasmids (pTM). Numbers refer to amino acids of NS5A of the JFH-1 isolate. For details refer to figure 1A. B. Huh7-Lunet cells were transfected with luciferase reporter replicons containing wt or mutant sequences as indicated. RNA replication of replicons was determined by measuring luciferase activity in cell lysates at 24 h, 48 h and 72 h post transfection relative to 4 h to normalize for transfection efficiency. Diagrams show mean values +/− SD from three independent experiments. C. Huh7-Lunet T7 cells were transfected with plasmids encoding the NS3 to NS5B polyprotein of genotype 2a (JFH-1) containing a wt sequence or the indicated point mutants or with empty vector (mock). Cells were fixed 24 h post transfection and subjected to immunofluorescence analysis using PI4P and NS5A specific antibodies. PI4P fluorescence intensity was quantified in NS5A positive cells or randomly chosen cells (mock) by ImageJ analysis. PI4P levels were normalized to non-transfected cells (mock, grey bar). Data represent mean values +/− SEM of thirty analyzed cells per condition. **, p<0.01. D. Huh7-Lunet T7 cells were transfected with the same set of plasmids as in panel C. Newly synthesized proteins were radiolabeled and cell lysates subjected to immunoprecipitation using NS5A (lower panel) or HA-specific antibodies (upper panel). Samples were analyzed by SDS-PAGE and autoradiography. The ratio between p58 NS5A and p56 NS5A was obtained by quantitative analysis of scanned autoradiographs. Data represent mean +/− SD from two independent experiments. E: Huh7-Lunet T7 cells were cotransfected with plasmids encoding HA-tagged PI4KIIIα (HA-PI4K) and the NS3 to NS5B polyprotein of genotype 2a (JFH-1) containing either the wt sequence of NS5A, the triple alanine mutation mutHIT or the phospho-mimetic mutation T210E in the context of either wt NS5A (mutHIE) or of the triple alanine mutant mutHIT (mutAAE). Newly synthesized proteins were radiolabeled and cell lysates subjected to immunoprecipitation using NS5A (lower panel) or HA-specific antibodies (upper panel). Samples were analyzed by SDS-PAGE and autoradiography. F: Quantitative analysis of PI4KIIIα pull-down efficiency. Experiments as shown in panel E were quantified by phosphoimaging. Coprecipitation efficiency was normalized to the total amounts of HA-PI4KIIIα and calculated relative to HA-PI4KIIIα pull-down by NS5A wt. G: Luciferase reporter replicons as depicted in Fig. 3A carrying the indicated mutations in NS5A were transfected into Huh7-Lunet cells. Replication efficiency is expressed as luciferase activity (RLU) 48 h relative to 4 h after transfection. JFH-1 wt replicons (wt, green) and a mutant harboring a deletion within NS5B (ΔGDD, red) served as positive and negative controls, respectively.
(TIF)

Figure S8 **PI4P levels induced by rescued mutHIT replicon do not correlate with replication.** A: Mean values and standard deviations of PI4P quantitation obtained from data of figure 8D, relative to Huh7-Lunet cells transfected with the negative control ΔGDD replicon. B: Correlation of the replication measured by the fluorescence intensity of eGFP tagged replicons and the quantitation of the PI4P fluorescence intensity of corresponding cells expressing NS3-5A of JFH-1 isolate. Quantitations of fluorescence intensities are obtained using ImageJ analysis (IntDen read-out) and indicated as arbitrary units (AU). Blue dots show the correlation of a wt eGFP replicon and red diamonds represent values of the rescued eGFP-tagged mutHIT replicon. Best fits are shown as blue and red lines, respectively. Pearson's correlation coefficient (R^2^) and corresponding p-value is given for each panel. n.s., not significant.
(TIF)

Table S1 Primer sequences used for cloning.
(DOC)

## References

[1] Bartenschlager R, Cosset FL, Lohmann V (2010) Hepatitis C virus replication cycle. J Hepatol 53: 583–585. 20579761 10.1016/j.jhep.2010.04.015

[2] Moradpour D, Penin F, Rice CM (2007) Replication of hepatitis C virus. Nat Rev Microbiol 5: 453–463. 17487147 10.1038/nrmicro1645

[3] Gouttenoire J, Penin F, Moradpour D (2010) Hepatitis C virus nonstructural protein 4B: a journey into unexplored territory. Rev Med Virol 20: 117–129. 20069613 10.1002/rmv.640

[4] Huang Y, Staschke K, De Francesco R, Tan SL (2007) Phosphorylation of hepatitis C virus NS5A nonstructural protein: a new paradigm for phosphorylation-dependent viral RNA replication? Virology 364: 1–9. 17400273 10.1016/j.virol.2007.01.042

[5] Gosert R, Egger D, Lohmann V, Bartenschlager R, Blum HE, et al (2003) Identification of the hepatitis C virus RNA replication complex in huh-7 cells harboring subgenomic replicons. J Virol 77: 5487–5492. 12692249 10.1128/JVI.77.9.5487-5492.2003 PMC153965

[6] Egger D, Wolk B, Gosert R, Bianchi L, Blum HE, et al (2002) Expression of hepatitis C virus proteins induces distinct membrane alterations including a candidate viral replication complex. J Virol 76: 5974–5984. 12021330 10.1128/JVI.76.12.5974-5984.2002 PMC136238

[7] Quinkert D, Bartenschlager R, Lohmann V (2005) Quantitative analysis of the hepatitis C virus replication complex. J Virol 79: 13594–13605. 16227280 10.1128/JVI.79.21.13594-13605.2005 PMC1262582

[8] Miyanari Y, Hijikata M, Yamaji M, Hosaka M, Takahashi H, et al (2003) Hepatitis C virus non-structural proteins in the probable membranous compartment function in viral genome replication. J Biol Chem 278: 50301–50308. 12963739 10.1074/jbc.M305684200

[9] Welsch S, Miller S, Romero-Brey I, Merz A, Bleck CK, et al (2009) Composition and three-dimensional architecture of the dengue virus replication and assembly sites. Cell Host Microbe 5: 365–375. 19380115 10.1016/j.chom.2009.03.007 PMC7103389

[10] Ferraris P, Blanchard E, Roingeard P (2010) Ultrastructural and biochemical analyses of hepatitis C virus-associated host cell membranes. J Gen Virol 91: 2230–2237. 20484561 10.1099/vir.0.022186-0

[11] Reiss S, Rebhan I, Backes P, Romero-Brey I, Erfle H, et al (2011) Recruitment and activation of a lipid kinase by hepatitis C virus NS5A is essential for integrity of the membranous replication compartment. Cell Host Microbe 9: 32–45. 21238945 10.1016/j.chom.2010.12.002 PMC3433060

[12] Sir D, Kuo CF, Tian Y, Liu HM, Huang EJ, et al (2012) Replication of hepatitis C virus RNA on autophagosomal membranes. J Biol Chem 287: 18036–18043. 22496373 10.1074/jbc.M111.320085 PMC3365724

[13] Balla A, Balla T (2006) Phosphatidylinositol 4-kinases: old enzymes with emerging functions. Trends Cell Biol 16: 351–361. 16793271 10.1016/j.tcb.2006.05.003

[14] Balla A, Tuymetova G, Tsiomenko A, Varnai P, Balla T (2005) A plasma membrane pool of phosphatidylinositol 4-phosphate is generated by phosphatidylinositol 4-kinase type-III alpha: studies with the PH domains of the oxysterol binding protein and FAPP1. Mol Biol Cell 16: 1282–1295. 15635101 10.1091/mbc.E04-07-0578 PMC551492

[15] Bianco A, Reghellin V, Donnici L, Fenu S, Alvarez R, et al (2012) Metabolism of phosphatidylinositol 4-kinase IIIalpha-dependent PI4P Is subverted by HCV and is targeted by a 4-anilino quinazoline with antiviral activity. PLoS Pathog 8: e1002576. 22412376 10.1371/journal.ppat.1002576 PMC3297592

[16] D'Angelo G, Vicinanza M, Di Campli A, De Matteis MA (2008) The multiple roles of PtdIns(4)P – not just the precursor of PtdIns(4,5)P2. J Cell Sci 121: 1955–1963. 18525025 10.1242/jcs.023630

[17] Vaillancourt FH, Pilote L, Cartier M, Lippens J, Liuzzi M, et al (2009) Identification of a lipid kinase as a host factor involved in hepatitis C virus RNA replication. Virology 387: 5–10. 19304308 10.1016/j.virol.2009.02.039

[18] Tai AW, Benita Y, Peng LF, Kim SS, Sakamoto N, et al (2009) A functional genomic screen identifies cellular cofactors of hepatitis C virus replication. Cell Host Microbe 5: 298–307. 19286138 10.1016/j.chom.2009.02.001 PMC2756022

[19] Berger KL, Cooper JD, Heaton NS, Yoon R, Oakland TE, et al (2009) Roles for endocytic trafficking and phosphatidylinositol 4-kinase III alpha in hepatitis C virus replication. Proc Natl Acad Sci U S A 106: 7577–7582. 19376974 10.1073/pnas.0902693106 PMC2678598

[20] Borawski J, Troke P, Puyang X, Gibaja V, Zhao S, et al (2009) Class III phosphatidylinositol 4-kinase alpha and beta are novel host factor regulators of hepatitis C virus replication. J Virol 83: 10058–10074. 19605471 10.1128/JVI.02418-08 PMC2748049

[21] Trotard M, Lepere-Douard C, Regeard M, Piquet-Pellorce C, Lavillette D, et al (2009) Kinases required in hepatitis C virus entry and replication highlighted by small interference RNA screening. FASEB J 23: 3780–9. 19608626 10.1096/fj.09-131920

[22] Hsu NY, Ilnytska O, Belov G, Santiana M, Chen YH, et al (2010) Viral reorganization of the secretory pathway generates distinct organelles for RNA replication. Cell 141: 799–811. 20510927 10.1016/j.cell.2010.03.050 PMC2982146

[23] Li Q, Brass AL, Ng A, Hu Z, Xavier RJ, et al (2009) A genome-wide genetic screen for host factors required for hepatitis C virus propagation. Proc Natl Acad Sci U S A 106: 16410–16415. 19717417 10.1073/pnas.0907439106 PMC2752535

[24] Zhang L, Hong Z, Lin W, Shao RX, Goto K, et al (2012) ARF1 and GBF1 generate a PI4P-enriched environment supportive of hepatitis C virus replication. PLoS One 7: e32135. 22359663 10.1371/journal.pone.0032135 PMC3281116

[25] Bishe B, Syed GH, Field SJ, Siddiqui A (2012) Role of Phosphatidylinositol 4-Phosphate (PI4P) and Its Binding Protein GOLPH3 in Hepatitis C Virus Secretion. J Biol Chem 287: 27637–27647. 22745132 10.1074/jbc.M112.346569 PMC3431621

[26] Trotard M, Lepere-Douard C, Regeard M, Piquet-Pellorce C, Lavillette D, et al (2009) Kinases required in hepatitis C virus entry and replication highlighted by small interference RNA screening. FASEB J 23: 3780–3789. 19608626 10.1096/fj.09-131920

[27] Altan-Bonnet N, Balla T (2012) Phosphatidylinositol 4-kinases: hostages harnessed to build panviral replication platforms. Trends Biochem Sci 37: 293–302. 22633842 10.1016/j.tibs.2012.03.004 PMC3389303

[28] Vaillancourt FH, Brault M, Pilote L, Uyttersprot N, Gaillard ET, et al (2012) Evaluation of phosphatidylinositol-4-kinase IIIalpha as a hepatitis C virus drug target. J Virol 86: 11595–607. 22896614 10.1128/JVI.01320-12 PMC3486294

[29] Berger KL, Kelly SM, Jordan TX, Tartell MA, Randall G (2011) Hepatitis C virus stimulates the phosphatidylinositol 4-kinase III alpha-dependent phosphatidylinositol 4-phosphate production that is essential for its replication. J Virol 85: 8870–8883. 21697487 10.1128/JVI.00059-11 PMC3165839

[30] Tellinghuisen TL, Marcotrigiano J, Gorbalenya AE, Rice CM (2004) The NS5A protein of hepatitis C virus is a zinc metalloprotein. J Biol Chem 279: 48576–48587. 15339921 10.1074/jbc.M407787200

[31] Penin F, Brass V, Appel N, Ramboarina S, Montserret R, et al (2004) Structure and function of the membrane anchor domain of hepatitis C virus nonstructural protein 5A. J Biol Chem 279: 40835–40843. 15247283 10.1074/jbc.M404761200

[32] Huang L, Hwang J, Sharma SD, Hargittai MR, Chen Y, et al (2005) Hepatitis C Virus Nonstructural Protein 5A (NS5A) Is an RNA-binding Protein. J Biol Chem 280: 36417–36428. 16126720 10.1074/jbc.M508175200

[33] Tellinghuisen TL, Marcotrigiano J, Rice CM (2005) Structure of the zinc-binding domain of an essential component of the hepatitis C virus replicase. Nature 435: 374–379. 15902263 10.1038/nature03580 PMC1440517

[34] Appel N, Zayas M, Miller S, Krijnse-Locker J, Schaller T, et al (2008) Essential role of domain III of nonstructural protein 5A for hepatitis C virus infectious particle assembly. PLoS Pathog 4: e1000035. 18369481 10.1371/journal.ppat.1000035 PMC2268006

[35] Tellinghuisen TL, Foss KL, Treadaway J (2008) Regulation of hepatitis C virion production via phosphorylation of the NS5A protein. PLoS Pathog 4: e1000032. 18369478 10.1371/journal.ppat.1000032 PMC2265800

[36] Masaki T, Suzuki R, Murakami K, Aizaki H, Ishii K, et al (2008) Interaction of hepatitis C virus nonstructural protein 5A with core protein is critical for the production of infectious virus particles. J Virol 82: 7964–7976. 18524832 10.1128/JVI.00826-08 PMC2519576

[37] Kaneko T, Tanji Y, Satoh S, Hijikata M, Asabe S, et al (1994) Production of two phosphoproteins from the NS5A region of the hepatitis C viral genome. Biochem Biophys Res Commun 205: 320–326. 7999043 10.1006/bbrc.1994.2667

[38] Koch JO, Bartenschlager R (1999) Modulation of hepatitis C virus NS5A hyperphosphorylation by nonstructural proteins NS3, NS4A, and NS4B. J Virol 73: 7138–7146. 10438800 10.1128/jvi.73.9.7138-7146.1999 PMC104237

[39] Neddermann P, Clementi A, De Francesco R (1999) Hyperphosphorylation of the hepatitis C virus NS5A protein requires an active NS3 protease, NS4A, NS4B, and NS5A encoded on the same polyprotein. Journal of Virology 73: 9984–9991. 10559312 10.1128/jvi.73.12.9984-9991.1999 PMC113049

[40] Tanji Y, Kaneko T, Satoh S, Shimotohno K (1995) Phosphorylation of hepatitis C virus-encoded nonstructural protein NS5A. J Virol 69: 3980–3986. 7769656 10.1128/jvi.69.7.3980-3986.1995 PMC189129

[41] McCormick CJ, Brown D, Griffin S, Challinor L, Rowlands DJ, et al (2006) A link between translation of the hepatitis C virus polyprotein and polymerase function; possible consequences for hyperphosphorylation of NS5A. J Gen Virol 87: 93–102. 16361421 10.1099/vir.0.81180-0

[42] Kim J, Lee D, Choe J (1999) Hepatitis C virus NS5A protein is phosphorylated by casein kinase II. Biochem Biophys Res Commun 257: 777–781. 10208859 10.1006/bbrc.1999.0460

[43] Quintavalle M, Sambucini S, Summa E, Orsatti L, Talamo F, et al (2006) Hepatitis C virus NS5A is a direct substrate of CKI-alpha , a cellular kinase identified by inhibitor affinity chromatography using specific NS5A hyperphosphorylation inhibitors. J Biol Chem 282: 5536–44. 17166835 10.1074/jbc.M610486200

[44] Quintavalle M, Sambucini S, Di Pietro C, De Francesco R, Neddermann P (2006) The alpha isoform of protein kinase CKI is responsible for hepatitis C virus NS5A hyperphosphorylation. J Virol 80: 11305–11312. 16943283 10.1128/JVI.01465-06 PMC1642135

[45] Chen YC, Su WC, Huang JY, Chao TC, Jeng KS, et al (2010) Polo-like kinase 1 is involved in hepatitis C virus replication by hyperphosphorylating NS5A. J Virol 84: 7983–7993. 20534861 10.1128/JVI.00068-10 PMC2916529

[46] Blight KJ, Kolykhalov AA, Rice CM (2000) Efficient initiation of HCV RNA replication in cell culture. Science 290: 1972–1974. 11110665 10.1126/science.290.5498.1972

[47] Katze MG, Kwieciszewski B, Goodlett DR, Blakely CM, Neddermann P, et al (2000) Ser(2194) is a highly conserved major phosphorylation site of the hepatitis C virus nonstructural protein NS5A. Virology 278: 501–513. 11118372 10.1006/viro.2000.0662

[48] Appel N, Pietschmann T, Bartenschlager R (2005) Mutational analysis of hepatitis C virus nonstructural protein 5A: potential role of differential phosphorylation in RNA replication and identification of a genetically flexible domain. J Virol 79: 3187–3194. 15709040 10.1128/JVI.79.5.3187-3194.2005 PMC548472

[49] Evans MJ, Rice CM, Goff SP (2004) Phosphorylation of hepatitis C virus nonstructural protein 5A modulates its protein interactions and viral RNA replication. Proc Natl Acad Sci U S A 101: 13038–13043. 15326295 10.1073/pnas.0405152101 PMC516513

[50] Appel N, Herian U, Bartenschlager R (2005) Efficient rescue of hepatitis C virus RNA replication by trans-complementation with nonstructural protein 5A. J Virol 79: 896–909. 15613318 10.1128/JVI.79.2.896-909.2005 PMC538567

[51] Fridell RA, Qiu D, Valera L, Wang C, Rose RE, et al (2011) Distinct functions of NS5A in hepatitis C virus RNA replication uncovered by studies with the NS5A inhibitor BMS-790052. J Virol 85: 7312–7320. 21593143 10.1128/JVI.00253-11 PMC3126594

[52] Lohmann V, Hoffmann S, Herian U, Penin F, Bartenschlager R (2003) Viral and cellular determinants of hepatitis C virus RNA replication in cell culture. J Virol 77: 3007–3019. 12584326 10.1128/JVI.77.5.3007-3019.2003 PMC149776

[53] Neddermann P, Quintavalle M, Di Pietro C, Clementi A, Cerretani M, et al (2004) Reduction of hepatitis C virus NS5A hyperphosphorylation by selective inhibition of cellular kinases activates viral RNA replication in cell culture. J Virol 78: 13306–13314. 15542681 10.1128/JVI.78.23.13306-13314.2004 PMC524975

[54] Miyanari Y, Atsuzawa K, Usuda N, Watashi K, Hishiki T, et al (2007) The lipid droplet is an important organelle for hepatitis C virus production. Nat Cell Biol 9: 1089–1097. 17721513 10.1038/ncb1631

[55] Pietschmann T, Zayas M, Meuleman P, Long G, Appel N, et al (2009) Production of infectious genotype 1b virus particles in cell culture and impairment by replication enhancing mutations. PLoS Pathog 5: e1000475. 19521536 10.1371/journal.ppat.1000475 PMC2691593

[56] Kato T, Date T, Miyamoto M, Furusaka A, Tokushige K, et al (2003) Efficient replication of the genotype 2a hepatitis C virus subgenomic replicon. Gastroenterology 125: 1808–1817. 14724833 10.1053/j.gastro.2003.09.023

[57] Jones DM, Patel AH, Targett-Adams P, McLauchlan J (2009) The hepatitis C virus NS4B protein can trans-complement viral RNA replication and modulates production of infectious virus. J Virol 83: 2163–2177. 19073716 10.1128/JVI.01885-08 PMC2643717

[58] Schaller T, Appel N, Koutsoudakis G, Kallis S, Lohmann V, et al (2007) Analysis of hepatitis C virus superinfection exclusion by using novel fluorochrome gene-tagged viral genomes. J Virol 81: 4591–4603. 17301154 10.1128/JVI.02144-06 PMC1900174

[59] Ahn J, Chung KS, Kim DU, Won M, Kim L, et al (2004) Systematic identification of hepatocellular proteins interacting with NS5A of the hepatitis C virus. J Biochem Mol Biol 37: 741–748. 15607035 10.5483/bmbrep.2004.37.6.741

[60] Tai AW, Salloum S (2011) The role of the phosphatidylinositol 4-kinase PI4KA in hepatitis C virus-induced host membrane rearrangement. PLoS One 6: e26300. 22022594 10.1371/journal.pone.0026300 PMC3192179

[61] Lim YS, Hwang SB (2011) Hepatitis C Virus NS5A protein interacts with phosphatidylinositol 4-kinase type III{alpha} and regulates viral propagation. J Biol Chem 286: 11290–8. 21297162 10.1074/jbc.M110.194472 PMC3064185

[62] Love RA, Brodsky O, Hickey MJ, Wells PA, Cronin CN (2009) Crystal structure of a novel dimeric form of NS5A domain I protein from hepatitis C virus. J Virol 83: 4395–4403. 19244328 10.1128/JVI.02352-08 PMC2668466

[63] Feuerstein S, Solyom Z, Aladag A, Favier A, Schwarten M, et al (2012) Transient structure and SH3 interaction sites in an intrinsically disordered fragment of the hepatitis C virus protein NS5A. J Mol Biol 420: 310–323. 22543239 10.1016/j.jmb.2012.04.023

[64] Qiu D, Lemm JA, O'Boyle DR, Sun JH, Nower PT, et al (2011) The effects of NS5A inhibitors on NS5A phosphorylation, polyprotein processing and localization. J Gen Virol 92: 2502–2511. 21795470 10.1099/vir.0.034801-0

[65] Penin F, Dubuisson J, Rey FA, Moradpour D, Pawlotsky JM (2004) Structural biology of hepatitis C virus. Hepatology 39: 5–19. 14752815 10.1002/hep.20032

[66] Lindenbach BD, Pragai BM, Montserret R, Beran RK, Pyle AM, et al (2007) The C terminus of hepatitis C virus NS4A encodes an electrostatic switch that regulates NS5A hyperphosphorylation and viral replication. J Virol 81: 8905–8918. 17581983 10.1128/JVI.00937-07 PMC1951449

[67] Pietschmann T, Lohmann V, Rutter G, Kurpanek K, Bartenschlager R (2001) Characterization of cell lines carrying self-replicating hepatitis C virus RNAs. J Virol 75: 1252–1264. 11152498 10.1128/JVI.75.3.1252-1264.2001 PMC114031

[68] Amako Y, Syed GH, Siddiqui A (2011) Protein kinase D negatively regulates hepatitis C virus secretion through phosphorylation of oxysterol-binding protein and ceramide transfer protein. J Biol Chem 286: 11265–11274. 21285358 10.1074/jbc.M110.182097 PMC3064182

[69] Amako Y, Sarkeshik A, Hotta H, Yates J III, Siddiqui A (2009) Role of Oxysterol Binding Protein in Hepatitis C Virus infection. J Virol 83: 9237–46. 19570870 10.1128/JVI.00958-09 PMC2738263

[70] Macpherson JI, Sidders B, Wieland S, Zhong J, Targett-Adams P, et al (2011) An integrated transcriptomic and meta-analysis of hepatoma cells reveals factors that influence susceptibility to HCV infection. PLoS One 6: e25584. 22046242 10.1371/journal.pone.0025584 PMC3201949

[71] Zhao XH, Bondeva T, Balla T (2000) Characterization of recombinant phosphatidylinositol 4-kinase beta reveals auto- and heterophosphorylation of the enzyme. J Biol Chem 275: 14642–14648. 10799551 10.1074/jbc.275.19.14642

[72] Bondeva T, Pirola L, Bulgarelli-Leva G, Rubio I, Wetzker R, et al (1998) Bifurcation of lipid and protein kinase signals of PI3Kgamma to the protein kinases PKB and MAPK. Science 282: 293–296. 9765155 10.1126/science.282.5387.293

[73] Dhand R, Hiles I, Panayotou G, Roche S, Fry MJ, et al (1994) PI 3-kinase is a dual specificity enzyme: autoregulation by an intrinsic protein-serine kinase activity. EMBO J 13: 522–533. 8313897 10.1002/j.1460-2075.1994.tb06290.x PMC394841

[74] Walker EH, Perisic O, Ried C, Stephens L, Williams RL (1999) Structural insights into phosphoinositide 3-kinase catalysis and signalling. Nature 402: 313–320. 10580505 10.1038/46319

[75] Lempiainen H, Halazonetis TD (2009) Emerging common themes in regulation of PIKKs and PI3Ks. EMBO J 28: 3067–3073. 19779456 10.1038/emboj.2009.281 PMC2752028

[76] Walker EH, Pacold ME, Perisic O, Stephens L, Hawkins PT, et al (2000) Structural determinants of phosphoinositide 3-kinase inhibition by wortmannin, LY294002, quercetin, myricetin, and staurosporine. Mol Cell 6: 909–919. 11090628 10.1016/s1097-2765(05)00089-4

[77] Hausser A, Storz P, Martens S, Link G, Toker A, et al (2005) Protein kinase D regulates vesicular transport by phosphorylating and activating phosphatidylinositol-4 kinase IIIbeta at the Golgi complex. Nat Cell Biol 7: 880–886. 16100512 10.1038/ncb1289 PMC1458033

[78] Ide Y, Tanimoto A, Sasaguri Y, Padmanabhan R (1997) Hepatitis C virus NS5A protein is phosphorylated in vitro by a stably bound protein kinase from HeLa cells and by cAMP-dependent protein kinase A-alpha catalytic subunit. Gene 201: 151–158. 9409782 10.1016/s0378-1119(97)00440-x

[79] Romero-Brey I, Merz A, Chiramel A, Lee JY, Chlanda P, et al (2012) Three-dimensional architecture and biogenesis of membrane structures associated with hepatitis C virus replication. PLoS Pathog 8: e1003056. 23236278 10.1371/journal.ppat.1003056 PMC3516559

[80] Tu H, Gao L, Shi ST, Taylor DR, Yang T, et al (1999) Hepatitis C virus RNA polymerase and NS5A complex with a SNARE-like protein. Virology 263: 30–41. 10544080 10.1006/viro.1999.9893

[81] Gao L, Aizaki H, He JW, Lai MM (2004) Interactions between viral nonstructural proteins and host protein hVAP-33 mediate the formation of hepatitis C virus RNA replication complex on lipid raft. J Virol 78: 3480–3488. 15016871 10.1128/JVI.78.7.3480-3488.2004 PMC371042

[82] Binder M, Kochs G, Bartenschlager R, Lohmann V (2007) Hepatitis C virus escape from the interferon regulatory factor 3 pathway by a passive and active evasion strategy. Hepatology 46: 1365–1374. 17668876 10.1002/hep.21829

[83] Backes P, Quinkert D, Reiss S, Binder M, Zayas M, et al (2010) Role of annexin A2 in the production of infectious hepatitis C virus particles. J Virol 84: 5775–5789. 20335258 10.1128/JVI.02343-09 PMC2876593

[84] Kaul A, Stauffer S, Berger C, Pertel T, Schmitt J, Kallis S, et al (2009) Essential role of cyclophilin A for hepatitis C virus replication and virus production and possible link to polyprotein cleavage kinetics. PLoS Pathog 5: e1000546. 19680534 10.1371/journal.ppat.1000546 PMC2718831

[85] Simister P, Schmitt M, Geitmann M, Wicht O, Danielson UH, et al (2009) Structural and functional analysis of hepatitis C virus strain JFH1 polymerase. J Virol 83: 11926–11939. 19740982 10.1128/JVI.01008-09 PMC2772714

[86] Binder M, Quinkert D, Bochkarova O, Klein R, Kezmic N, et al (2007) Identification of determinants involved in initiation of hepatitis C virus RNA synthesis by using intergenotypic replicase chimeras. J Virol 81: 5270–5283. 17344294 10.1128/JVI.00032-07 PMC1900214

[87] Kaul A, Woerz I, Meuleman P, Leroux-Roels G, Bartenschlager R (2007) Cell culture adaptation of hepatitis C virus and in vivo viability of an adapted variant. J Virol 81: 13168–13179. 17881454 10.1128/JVI.01362-07 PMC2169131

[88] Fuerst TR, Niles EG, Studier FW, Moss B (1986) Eukaryotic transient-expression system based on recombinant vaccinia virus that synthesizes bacteriophage T7 RNA polymerase. Proc Natl Acad Sci U S A 83: 8122–8126. 3095828 10.1073/pnas.83.21.8122 PMC386879

