# Supplementary figures and images for: The Lipid Kinase Phosphatidylinositol-4 Kinase III Alpha Regulates the Phosphorylation Status of Hepatitis C Virus NS5A
Source: PLoS Pathog. 2013 May 9;9(5):e1003359. doi: 10.1371/journal.ppat.1003359 (PMC3649985; doi:10.1371/journal.ppat.1003359)

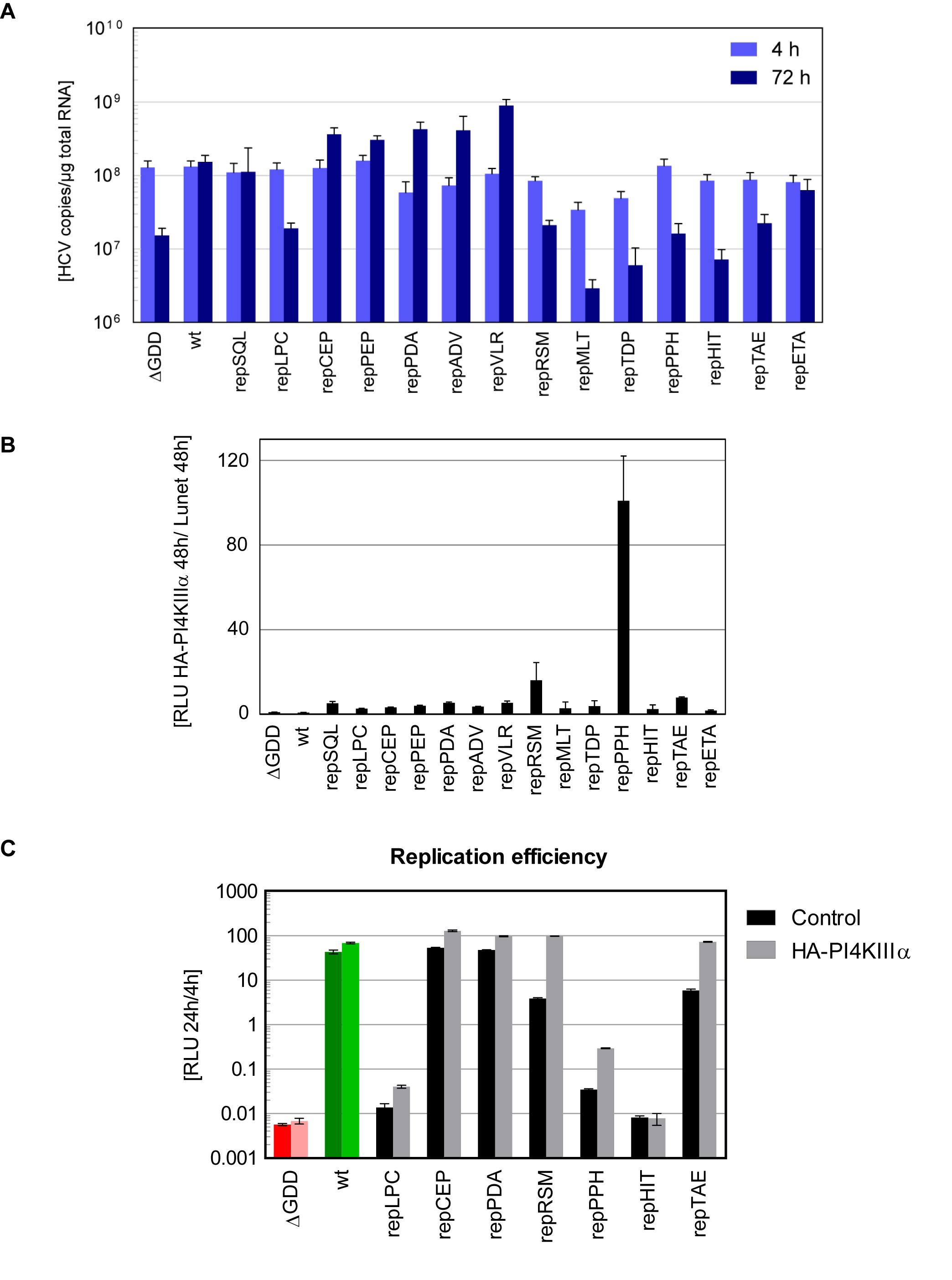

Supplement: Figure S1 — Impact of triple alanine mutations on HCV RNA replication. A: Huh7-Lunet cells were transfected with luciferase reporter replicons bearing the indicated triple alanine substitutions. JFH-1 wt replicons (wt) and a mutant harboring a deletion within NS5B (ΔGDD) served as positive and negative controls, respectively. Total cellular RNA was extracted 4 h (light blue) or 72 h (dark blue) after transfection. HCV RNA was quantified by quantitative RT-PCR and is depicted as non-normalized HCV RNA copies per µg of total cellular RNA at the respective time points. B: Naïve Huh7-Lunet cells (control) or Huh7-Lunet cells stably overexpressing HA-tagged PI4KIIIα (HA-PI4KIIIα) were transfected with luciferase reporter replicons as described in the legend of Fig. 3D. Depicted are the normalized ratios of luciferase activity 48 h after transfection in HA-PI4KIIIα overexpressing cells compared to naïve Huh7-Lunet cells. C: Replication efficiency at 24 h relative to 4 h after transfection of a subset of triple alanine mutants in naïve Huh7-Lunet cells (Control, black bars) or in Huh7-Lunet cells overexpressing HA-PI4KIIIα (grey bars) as shown in Fig. 3D. JFH-1 wt replicons (wt, green bars) and a mutant harboring a deletion within NS5B (ΔGDD, red bars) served as positive and negative controls, respectively. (TIF) [file ppat.1003359.s001.tif]

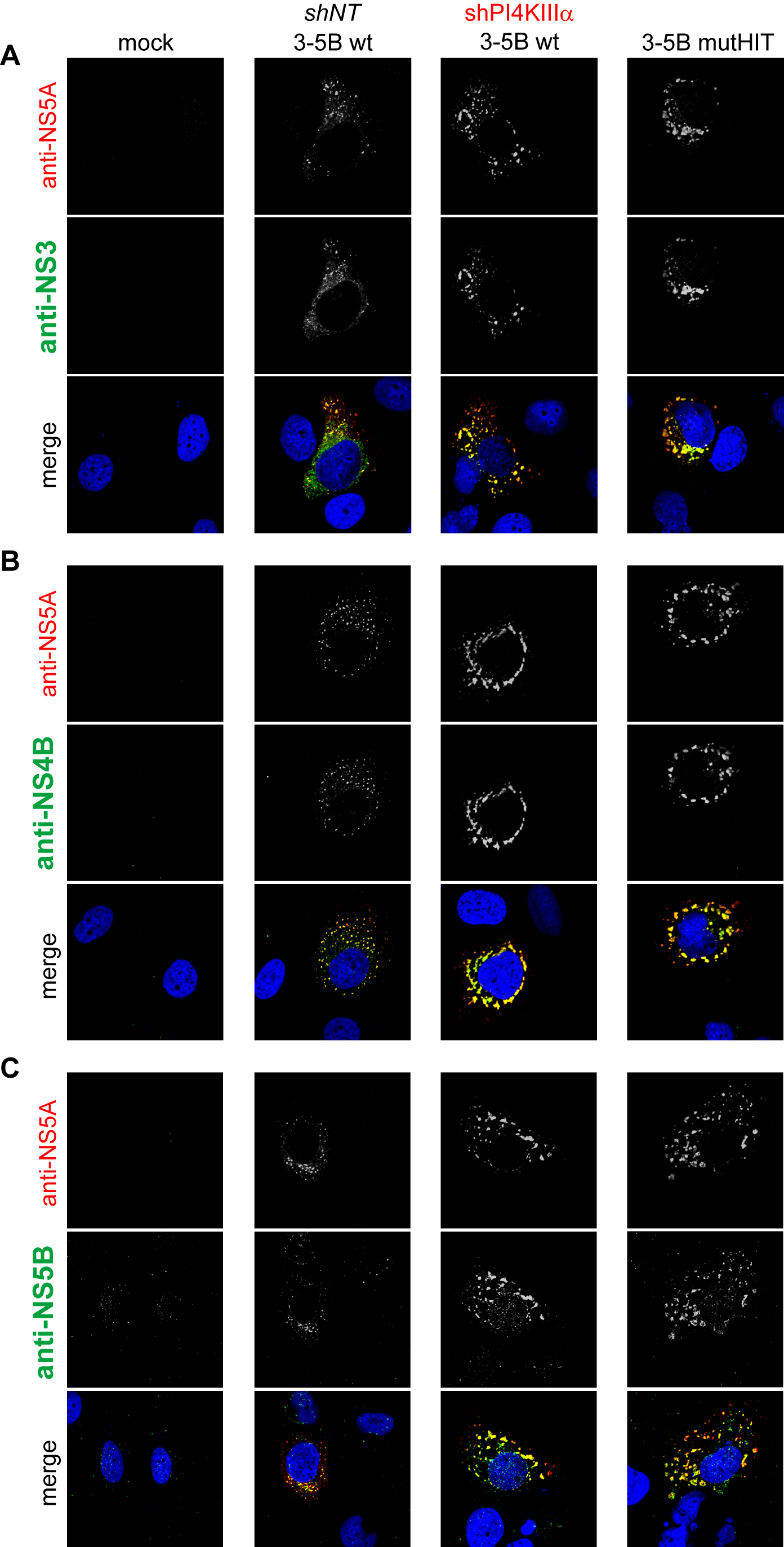

Supplement: Figure S2 — Subcellular localization of NS3, NS4B and NS5B relative to NS5A. Huh7-Lunet T7 cells with stable knockdown of PI4KIIIα (shPI4KIIIα) or control cells (shNT) were transfected with plasmids encoding the NS3 to NS5B polyprotein of genotype 2a (JFH-1) containing a wt sequence or the NS5A triple alanine mutant mutHIT or with empty plasmid (mock). 24 h post transfection NS5A (red) and an additional nonstructural protein (A: NS3, B: NS4B or C: NS5B, green), were detected with specific antibodies and nuclear DNA was stained with DAPI (blue). Note the punctuate staining pattern of NS3, NS4B, NS5A and NS5B in 3-5B wt transfected cells compared to formation of “clusters” in cells with stable knockdown of PI4KIIIα or expressing mutant polyproteins and the consistent colocalization of all nonstructural proteins for each experimental condition. (TIF) [file ppat.1003359.s002.tif]

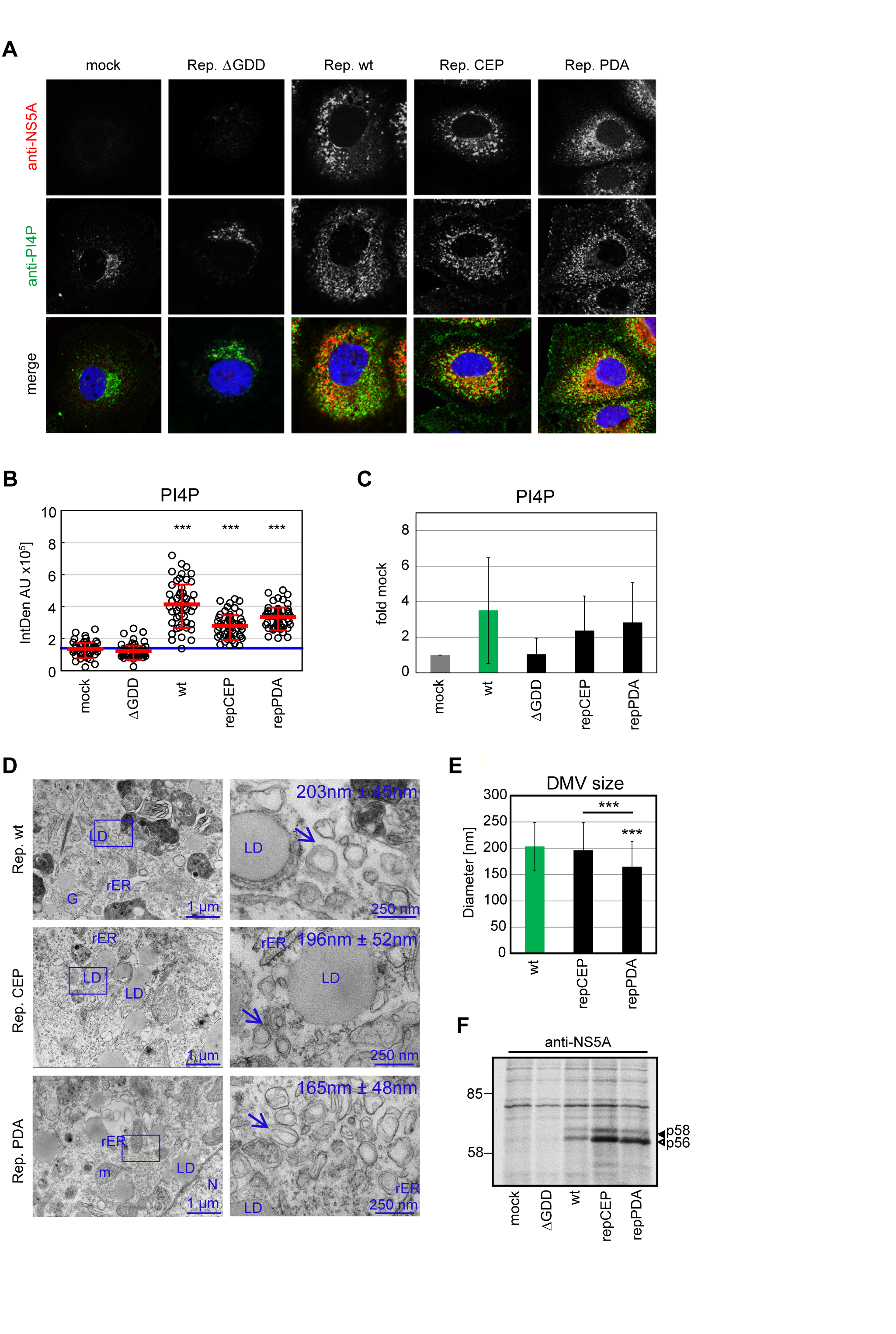

Supplement: Figure S3 — Characterization of NS5A mutants not impaired in RNA replication. Huh7-Lunet cells were transfected with subgenomic JFH-1 reporter replicons of either wt (wt), containing a deletion within NS5B (ΔGDD) or bearing indicated triple alanine substitutions. A: 48 h post transfection NS5A (red) or PI4P (green), respectively, was detected with specific antibodies and DAPI was used to stain nuclei (blue). B: Quantitative analysis of PI4P fluorescence intensity of cells as shown in panel A by ImageJ analysis (IntDen read-out). Error bars indicate the mean +/− SD of 50 NS5A positive cells analyzed per condition. Significance of increased PI4P levels relative to mock was measured by a paired t-test and is indicated. ***, p<0.001. The blue line points to the mean of PI4P IntDen values of untransfected cells (mock). C: Mean values and standard deviations of PI4P quantitation obtained from data of figure S3B, relative to mock transfected cells. D: Cells were fixed and prepared for EM analysis 48 h post transfection. Consecutive enlargements of the boxed areas are shown from left to right. For details see legend to fig. 4. E: Average diameter of DMVs detected in the indicated replicon cells 48 h after transfection of replicon RNA. Error bars indicate the mean +/− SD of seventy vesicles. Significance of differences in DMV sizes was measured by a paired t-test and is indicated ***, p<0.001. F: Proteins were radiolabeled 48 h after transfection and cell lysates were subjected to immunoprecipitation using NS5A-specific antibodies. Samples were analyzed by SDS-PAGE and autoradiography. (TIF) [file ppat.1003359.s003.tif]

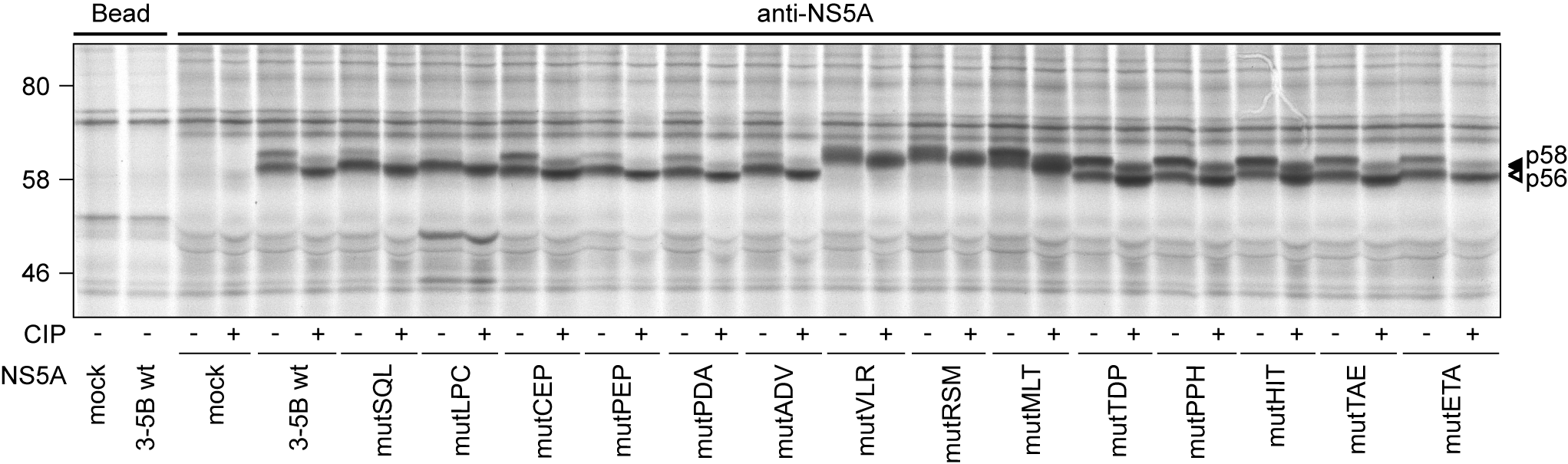

Supplement: Figure S4 — Phosphatase treatment of triple alanine mutants. Huh7-Lunet T7 cells were transfected with plasmids encoding the NS3 to NS5B polyprotein of genotype 2a (JFH-1) containing a wt sequence or triple alanine mutations as indicated or with empty plasmid (mock). Newly synthesized proteins were radiolabeled and cell lysates subjected to immunoprecipitations using NS5A-specific antibodies. After immunoprecipation samples were treated with calf intestine phosphatase (CIP) or mock treated as indicated and analyzed by SDS-PAGE and autoradiography. (TIF) [file ppat.1003359.s004.tif]

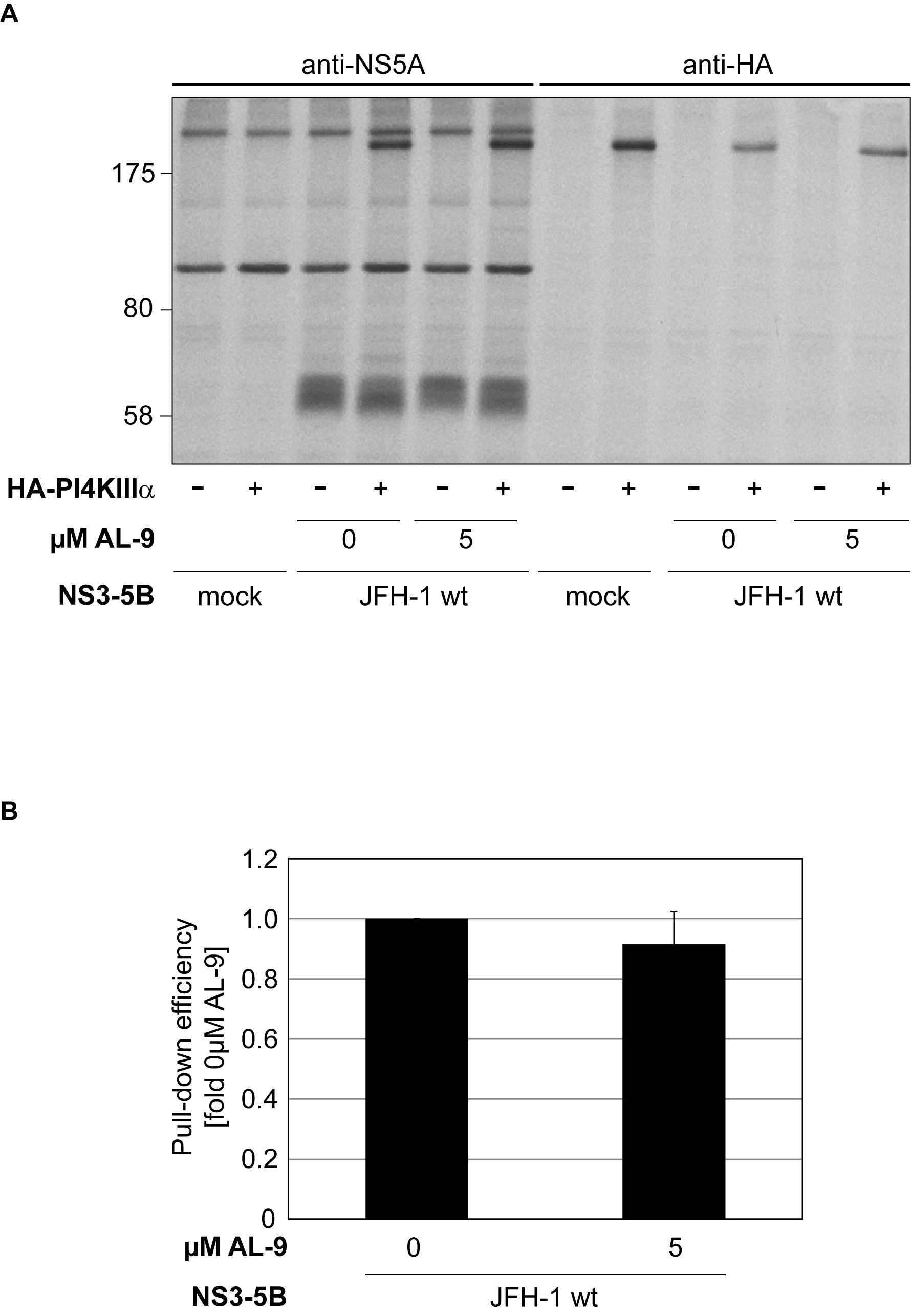

Supplement: Figure S5 — Specific inhibition of PI4KIIIα activity by AL-9 does not affect NS5A- PI4KIIIα binding. A: Huh7-Lunet T7 cells were transfected with plasmids encoding the NS3 to NS5B polyprotein of genotype 2a (JFH-1) and HA-tagged PI4KIIIα (PI4KIIIα). Starting at 7 h post transfection, cells were incubated with 5 µM of AL-9 or DMSO. Newly synthesized proteins were radiolabeled and cell lysates subjected to immunoprecipitation using NS5A or HA-specific antibodies. B: Quantitative analysis of PI4KIIIα pull-down efficiency. Experiments as shown in panel A were quantified by phosphoimaging. Coprecipitation efficiency was normalized to the total amounts of HA-PI4KIIIα and calculated relative to PI4KIIIα pull-down by NS5A. Error bars indicate mean values +/− SD of two independent experiments analyzed in duplicates. (TIF) [file ppat.1003359.s005.tif]

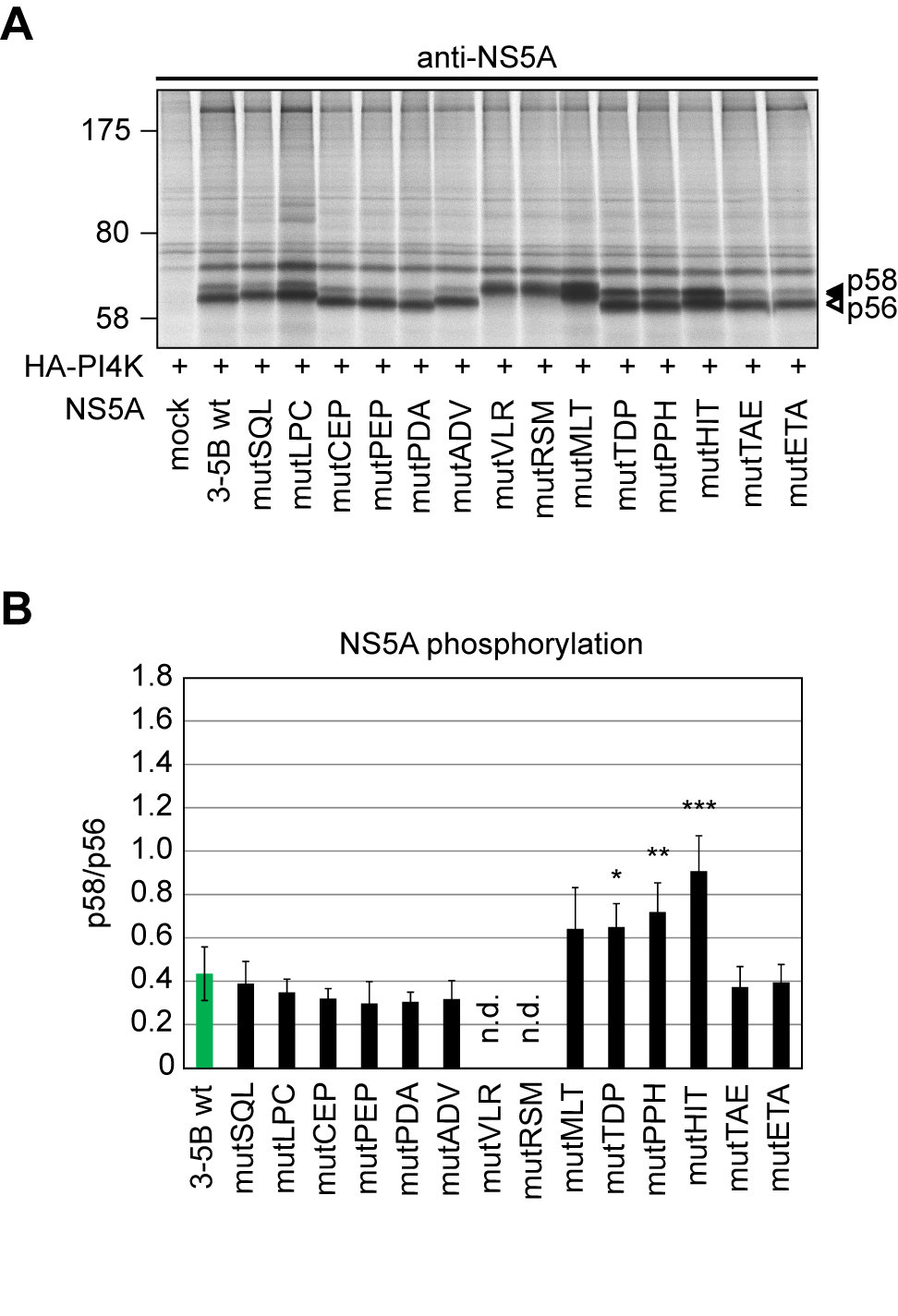

Supplement: Figure S6 — PI4KIIIα overexpression modulates phosphorylation of NS5A mutants. A: Huh7-Lunet T7 cells were cotransfected with plasmids encoding the NS3 to NS5B polyprotein of genotype 2a (JFH-1) containing triple alanine mutations in NS5A domain I as indicated and HA-tagged PI4KIIIα (HA-PI4K). Newly synthesized proteins were radiolabeled and cell lysates subjected to immunoprecipitations using NS5A-specific antibodies. Samples were analyzed by 10% SDS-PAGE and autoradiography. B: Quantitative analysis of the NS5A p58/p56 ratio. Bands corresponding to NS5A p58 and p56, respectively, as shown in panel A were individually quantified by phosphoimaging to obtain a p58/p56 ratio. Error bars indicate mean values +/− SD of two independent experiments analyzed in duplicates. Significances were compared to the wt polyprotein and calculated by paired t-tests.*, p<0.05; **, p<0.01; ***, p<0.001. n.d. not determined due to insufficient resolution of p56 and p58. (TIF) [file ppat.1003359.s006.tif]

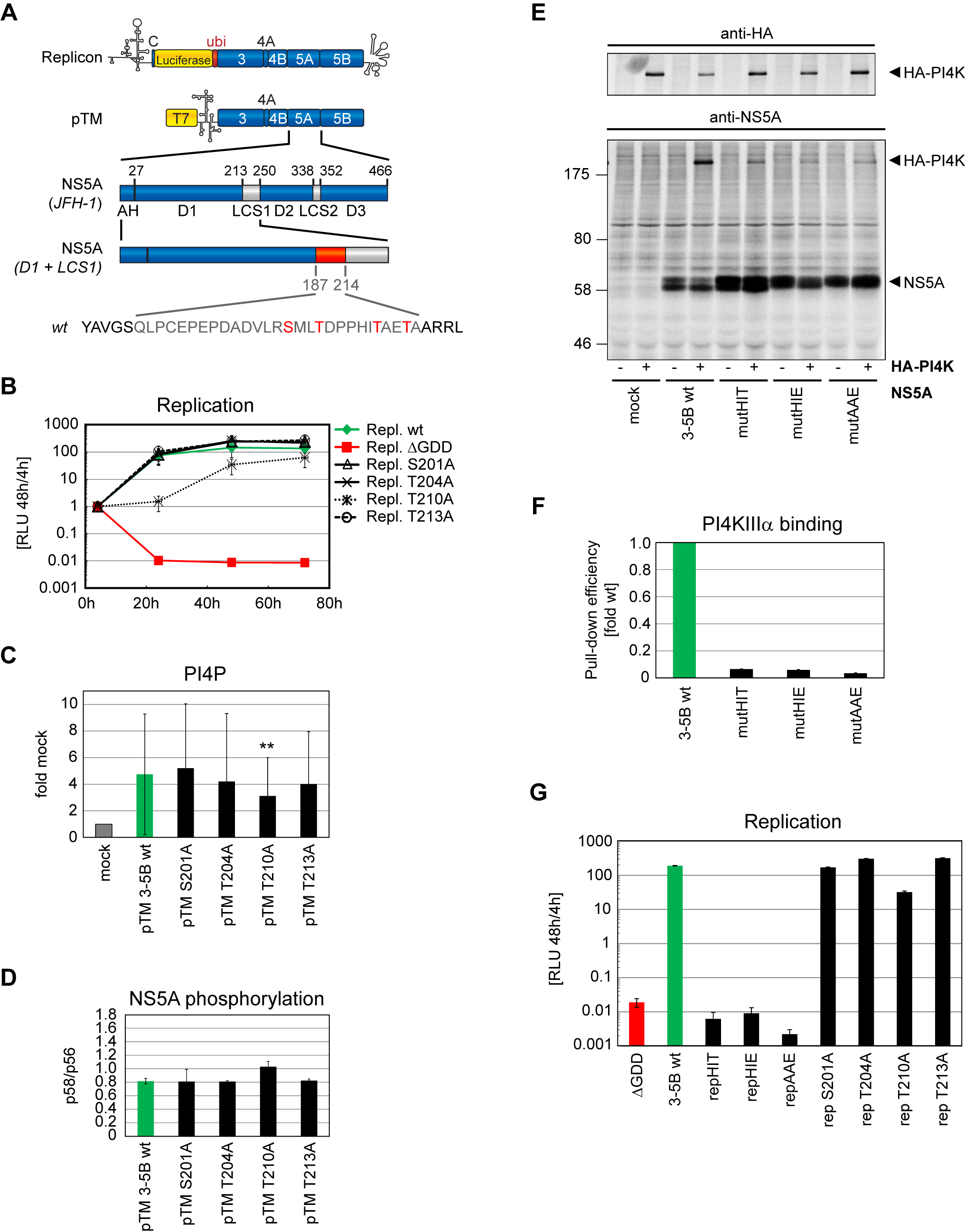

Supplement: Figure S7 — Analysis of potential NS5A phosphorylation sites within the PI4KIIIα binding motif. A: Scheme depicting possible NS5A phosphorylation sites within the PI4KIIIα binding motif that were individually mutated to alanine within the context of subgenomic replicons or NS3 to NS5B polyprotein expression plasmids (pTM). Numbers refer to amino acids of NS5A of the JFH-1 isolate. For details refer to figure 1A. B. Huh7-Lunet cells were transfected with luciferase reporter replicons containing wt or mutant sequences as indicated. RNA replication of replicons was determined by measuring luciferase activity in cell lysates at 24 h, 48 h and 72 h post transfection relative to 4 h to normalize for transfection efficiency. Diagrams show mean values +/− SD from three independent experiments. C. Huh7-Lunet T7 cells were transfected with plasmids encoding the NS3 to NS5B polyprotein of genotype 2a (JFH-1) containing a wt sequence or the indicated point mutants or with empty vector (mock). Cells were fixed 24 h post transfection and subjected to immunofluorescence analysis using PI4P and NS5A specific antibodies. PI4P fluorescence intensity was quantified in NS5A positive cells or randomly chosen cells (mock) by ImageJ analysis. PI4P levels were normalized to non-transfected cells (mock, grey bar). Data represent mean values +/− SEM of thirty analyzed cells per condition. **, p<0.01. D. Huh7-Lunet T7 cells were transfected with the same set of plasmids as in panel C. Newly synthesized proteins were radiolabeled and cell lysates subjected to immunoprecipitation using NS5A (lower panel) or HA-specific antibodies (upper panel). Samples were analyzed by SDS-PAGE and autoradiography. The ratio between p58 NS5A and p56 NS5A was obtained by quantitative analysis of scanned autoradiographs. Data represent mean +/− SD from two independent experiments. E: Huh7-Lunet T7 cells were cotransfected with plasmids encoding HA-tagged PI4KIIIα (HA-PI4K) and the NS3 to NS5B polyprotein of genotype 2a (JFH- [file ppat.1003359.s007.tif]

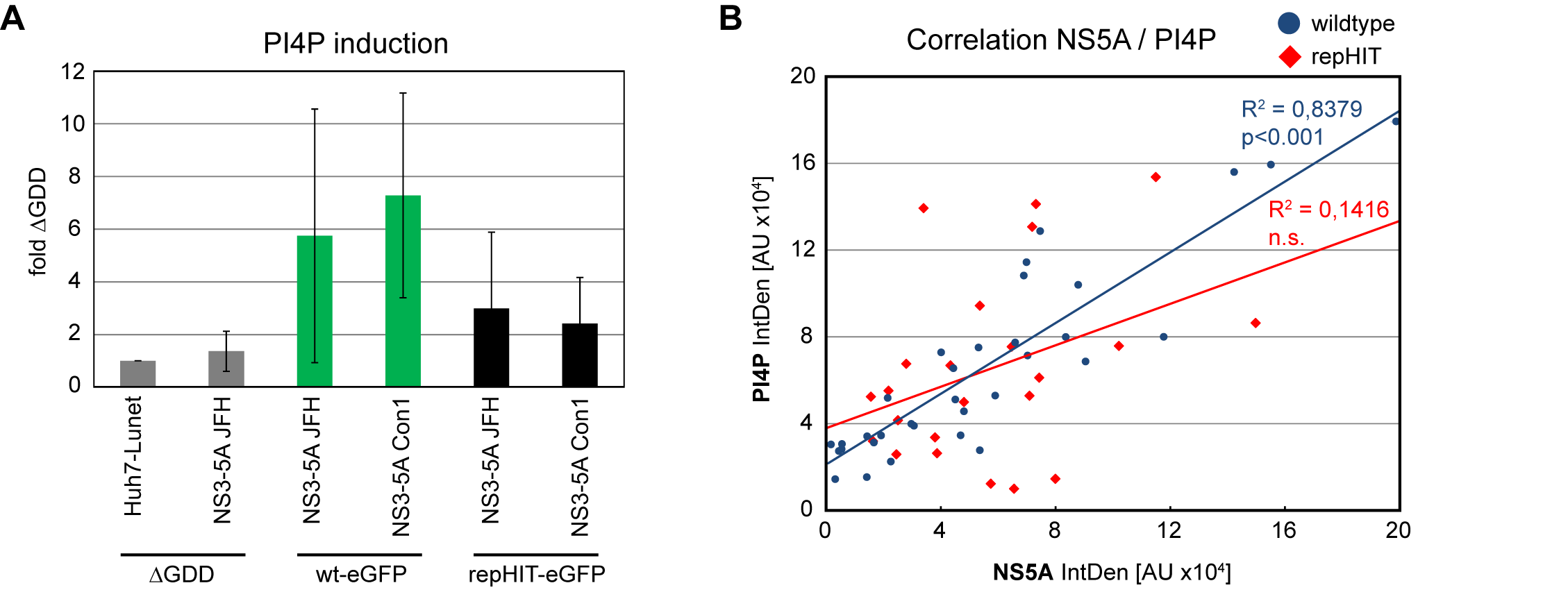

Supplement: Figure S8 — PI4P levels induced by rescued mutHIT replicon do not correlate with replication. A: Mean values and standard deviations of PI4P quantitation obtained from data of figure 8D, relative to Huh7-Lunet cells transfected with the negative control ΔGDD replicon. B: Correlation of the replication measured by the fluorescence intensity of eGFP tagged replicons and the quantitation of the PI4P fluorescence intensity of corresponding cells expressing NS3-5A of JFH-1 isolate. Quantitations of fluorescence intensities are obtained using ImageJ analysis (IntDen read-out) and indicated as arbitrary units (AU). Blue dots show the correlation of a wt eGFP replicon and red diamonds represent values of the rescued eGFP-tagged mutHIT replicon. Best fits are shown as blue and red lines, respectively. Pearson's correlation coefficient (R2) and corresponding p-value is given for each panel. n.s., not significant. (TIF) [file ppat.1003359.s008.tif]
