# Supplementary material for: The Lipid Kinase Phosphatidylinositol-4 Kinase III Alpha Regulates the Phosphorylation Status of Hepatitis C Virus NS5A
Source: PLoS Pathog. 2013 May 9;9(5):e1003359. doi: 10.1371/journal.ppat.1003359 (PMC3649985; doi:10.1371/journal.ppat.1003359)
Supplement: Table S1 — Primer sequences used for cloning. (DOC) [file ppat.1003359.s009.doc]

| **Primer** | **sequence (5’ 🡪 3’)** | **Construct** |
| --- | --- | --- |
| Forward F_MfeI | ccacgtcgcccctactcactacgtgacgga | **pTM NS3-5B/5AΔS1** |
| Reverse R_RsrII_JFH | caggggacgtcggaccgccggattcggc |  |
| F_del5AD1a | aattggctgacctcttacacggagggccag |  |
| R_del5AD1a | ctggccctccgtgtaagaggtcagccaatt |  |
| Forward F_MfeI | ccacgtcgcccctactcactacgtgacgga | **pTM NS3-5B/5AΔS2** |
| Reverse R_RsrII_JFH | caggggacgtcggaccgccggattcggc |  |
| F_del5AD1b | tttcctatcaattgctcctgggtggacggt |  |
| R_del5AD1b | accgtccacccaggagcaattgataggaaa |  |
| Forward F_BssHII | ggagggccagtgcgcgccgaaaccccccacgaa | **pTM NS3-5B/5AΔS3** |
| Reverse R_SacI | ggctcactgaggagctcgcctcagatggaggtg |  |
| F2_Del_alles_small | tctccagagtttttcgcgcggcgcttggca |  |
| R124_Del_alles | gaaaaactctggagaaggtagttggcaagg |  |
| Forward F_BssHII | ggagggccagtgcgcgccgaaaccccccacgaa | **pTM NS3-5B/5AΔS3x** |
| Reverse R_SacI | ggctcactgaggagctcgcctcagatggaggtg |  |
| F4_delLeft | tctccagagtttttccagcttccctgtgaa | *ΔS3A* |
| R124_Del_alles | gaaaaactctggagaaggtagttggcaagg |  |
| F_del10AA | tatgctgtcgggtccaggtccatgctaaca | *ΔS3B* |
| R_del10AA | ggacccgacagcataggaatta |  |
| F3_delRight | gacgcagacgtattggcggcgcggcgcttg | *ΔS3C* |
| R3_delRight | caatacgtctgcgtcgggctcagg |  |
| Forward F_BssHII | ggagggccagtgcgcgccgaaaccccccacgaa | **pTM NS3-5B/5AmutXXX** |
| Reverse R_SacI | ggctcactgaggagctcgcctcagatggaggtg |  |
| F_3erMut_-6 | ctatgctgtcggggcagccgcgccctgtgaacct | *mutSQL* |
| R_3erMut_-6 | aggttcacagggcgcggctgccccgacagcatag |  |
| F_3erMut_-5 | gtcgggtcccaggccgcagcggaacctgagcccg | *mutLPC* |
| R_3erMut_-5 | cgggctcaggttccgctgcggcctgggacccgac |  |
| F_3erMut_-4 | gtcccagcttcccgcggccgcggagcccgacgcag | *mutCEP* |
| R_3erMut_-4 | ctgcgtcgggctccgcggccgcgggaagctgggac |  |
| F_3erMut_-3 | cttccctgtgaagcggccgcagacgcagacgtat | *mutPEP* |
| R_3erMut_-3 | atacgtctgcgtctgcggccgcttcacagggaag |  |
| F_3erMut_-2 | tgtgaacctgaggcagcggcagacgtattg | *mutPDA* |
| R_3erMut_-2 | caatacgtctgccgctgcctcaggttcaca |  |
| F_3erMut_-1 | ctgagcccgacgcagctgcgttgaggtcca | *mutADV* |
| R_3erMut_-1 | tggacctcaacgcagctgcgtcgggctcag |  |
| F_3erMut_0 | cgacgcagacgctgcggcgtccatgctaa | *mutVLR* |
| R_3erMut_0 | ttagcatggacgccgcagcgtctgcgtcg |  |
| F_3erMut_1 | agacgtattggcggctgcgctaacagatc | *mutRSM* |
| R_3erMut_1 | gatctgttagcgcagccgccaatacgtct |  |
| F_3erMut_2 | attgaggtccgcggcagctgatccgcccc | *mutMLT* |
| R_3erMut_2 | ggggcggatcagctgccgcggacctcaat |  |
| F_3erMut_3 | gtccatgctagctgcggctccccacatca | *mutTDP* |
| R_3erMut_3 | tgatgtggggagccgcagctagcatggac |  |
| F_3erMut_4 | gctaacagatgctgcggccatcacggcgg | *mutPPH* |
| R_3erMut_4 | ccgccgtgatggccgcagcatctgttagc |  |
| F_3erMut_5 | agatccgcccgccgccgctgcggagactg | *mutHIT* |
| R_3erMut_5 | cagtctccgcagcggcggcgggcggatct |  |
| F_3erMut_6 | gccccacatcgctgcggctactgcggcgc | *mutTAE* |
| R_3erMut_6 | gcgccgcagtagccgcagcgatgtggggc |  |
| F_3erMut_7 | catcacggcggctgcggcggcgcggcgct | *mutETA* |
| R_3erMut_7 | agcgccgcgccgccgcagccgccgtgatg |  |
| F5_SzuA | cgcagacgtattgagggctatgctaacaga | mutS201A |
| R5_SzuA | tctgttagcatagccctcaatacgtctgcg |  |
| F6_TzuA | ttgaggtccatgctagctgatccgccccac | mutT204A |
| R6_TzuA | gtggggcggatcagctagcatggacctcaa |  |
| F8_TzuA | tccgccccacatcgctgcggagactgcggc | mutT210A |
| R8_TzuA | gccgcagtctccgcagcgatgtggggcgga |  |
| F9_TzuA | atcacggcggaggcggcggcgcggcgcttg | mutT213A |
| R9_TzuA | caagcgccgcgccgccgcctccgccgtgat |  |
| F_3-5B_Con_MutHIT | cgacccctccgggctgccgcggagacggc | pTM NS3-5B/5AmutHIT Con1 |
| R_3-5B_Con_MutHIT | gccgtctccgcagcagcggcggaggggtcg |  |
